# Meta-analysis of Genome-Wide Association Studies for Extraversion: Findings from the Genetics of Personality Consortium

**DOI:** 10.1007/s10519-015-9735-5

**Published:** 2015-09-11

**Authors:** Stéphanie M. van den Berg, Marleen H. M. de Moor, Karin J. H. Verweij, Robert F. Krueger, Michelle Luciano, Alejandro Arias Vasquez, Lindsay K. Matteson, Jaime Derringer, Tõnu Esko, Najaf Amin, Scott D. Gordon, Narelle K. Hansell, Amy B. Hart, Ilkka Seppälä, Jennifer E. Huffman, Bettina Konte, Jari Lahti, Minyoung Lee, Mike Miller, Teresa Nutile, Toshiko Tanaka, Alexander Teumer, Alexander Viktorin, Juho Wedenoja, Abdel Abdellaoui, Goncalo R. Abecasis, Daniel E. Adkins, Arpana Agrawal, Jüri Allik, Katja Appel, Timothy B. Bigdeli, Fabio Busonero, Harry Campbell, Paul T. Costa, George Davey Smith, Gail Davies, Harriet de Wit, Jun Ding, Barbara E. Engelhardt, Johan G. Eriksson, Iryna O. Fedko, Luigi Ferrucci, Barbara Franke, Ina Giegling, Richard Grucza, Annette M. Hartmann, Andrew C. Heath, Kati Heinonen, Anjali K. Henders, Georg Homuth, Jouke-Jan Hottenga, William G. Iacono, Joost Janzing, Markus Jokela, Robert Karlsson, John P. Kemp, Matthew G. Kirkpatrick, Antti Latvala, Terho Lehtimäki, David C. Liewald, Pamela A. F. Madden, Chiara Magri, Patrik K. E. Magnusson, Jonathan Marten, Andrea Maschio, Hamdi Mbarek, Sarah E. Medland, Evelin Mihailov, Yuri Milaneschi, Grant W. Montgomery, Matthias Nauck, Michel G. Nivard, Klaasjan G. Ouwens, Aarno Palotie, Erik Pettersson, Ozren Polasek, Yong Qian, Laura Pulkki-Råback, Olli T. Raitakari, Anu Realo, Richard J. Rose, Daniela Ruggiero, Carsten O. Schmidt, Wendy S. Slutske, Rossella Sorice, John M. Starr, Beate St Pourcain, Angelina R. Sutin, Nicholas J. Timpson, Holly Trochet, Sita Vermeulen, Eero Vuoksimaa, Elisabeth Widen, Jasper Wouda, Margaret J. Wright, Lina Zgaga, Generation Scotland, David Porteous, Alessandra Minelli, Abraham A. Palmer, Dan Rujescu, Marina Ciullo, Caroline Hayward, Igor Rudan, Andres Metspalu, Jaakko Kaprio, Ian J. Deary, Katri Räikkönen, James F. Wilson, Liisa Keltikangas-Järvinen, Laura J. Bierut, John M. Hettema, Hans J. Grabe, Brenda W. J. H. Penninx, Cornelia M. van Duijn, David M. Evans, David Schlessinger, Nancy L. Pedersen, Antonio Terracciano, Matt McGue, Nicholas G. Martin, Dorret I. Boomsma

**Affiliations:** Department of Research Methodology, Measurement and Data-Analysis (OMD), Faculty of Behavioural, Management, and Social Sciences, University of Twente, PO Box 217, 7500 AE Enschede, The Netherlands; Department of Biological Psychology, VU University Amsterdam, Amsterdam, The Netherlands; Department of Clinical Child and Family Studies, VU University Amsterdam, Amsterdam, The Netherlands; Department of Methods, VU University Amsterdam, Amsterdam, The Netherlands; QIMR Berghofer Medical Research Institute, Brisbane, Australia; Department of Developmental Psychology and EMGO Institute for Health and Care Research, VU University Amsterdam, Amsterdam, The Netherlands; Department of Psychology, University of Minnesota, Minneapolis, USA; Department of Psychology, University of Edinburgh, Edinburgh, UK; Centre for Cognitive Ageing and Cognitive Epidemiology, University of Edinburgh, Edinburgh, UK; Donders Institute for Cognitive Neuroscience, Radboud University Nijmegen, Nijmegen, The Netherlands; Department of Psychiatry, Radboud University Nijmegen Medical Center, Nijmegen, The Netherlands; Department of Human Genetics, Radboud University Nijmegen Medical Center, Nijmegen, The Netherlands; Department of Cognitive Neuroscience, Radboud University Nijmegen Medical Center, Nijmegen, The Netherlands; Department of Psychology, University of Illinois at Urbana-Champaign, Champaign, IL USA; Estonian Genome Center, University of Tartu, Tartu, Estonia; Department of Epidemiology, Erasmus University Medical Center, Rotterdam, The Netherlands; Department of Human Genetics, University of Chicago, Chicago, IL USA; Department of Clinical Chemistry, Fimlab Laboratories and School of Medicine, University of Tampere, Tampere, Finland; MRC Human Genetics Unit, MRC IGMM, Western General Hospital, University of Edinburgh, Edinburgh, UK; Department of Psychiatry, University of Halle, Halle, Germany; Institute of Behavioural Sciences, University of Helsinki, Helsinki, Finland; Folkhälsan Research Center, Helsinki, Finland; Department of Psychiatry, Virginia Institute for Psychiatric and Behavioral Genetics, Virginia Commonwealth University, Richmond, VA USA; Institute of Genetics and Biophysics “A. Buzzati-Traverso” – CNR, Naples, Italy; National Institute on Aging, NIH, Baltimore, MD USA; Institute for Community Medicine, University Medicine Greifswald, Greifswald, Germany; Department of Medical Epidemiology and Biostatistics, Karolinska Institutet, Stockholm, Sweden; Department of Public Health, University of Helsinki, Helsinki, Finland; Department of Biostatistics, Center for Statistical Genetics, University of Michigan School of Public Health, Ann Arbor, MI USA; Pharmacotherapy & Outcomes Science, Virginia Commonwealth University, Richmond, VA USA; Department of Psychiatry, Washington University School of Medicine, St. Louis, MO USA; Department of Psychology, University of Tartu, Tartu, Estonia; Estonian Academy of Sciences, Tallinn, Estonia; Department of Psychiatry and Psychotherapy, University Medicine Greifswald, Greifswald, Germany; Istituto di Ricerca Genetica e Biomedica (IRGB), CNR, Monserrato, Italy; Usher Institute for Population Health Sciences and Informatics, University of Edinburgh, Edinburgh, UK; Behavioral Medicine Research Center, Duke University School of Medicine, Durham, NC USA; Medical Research Council Integrative Epidemiology Unit, School of Social and Community Medicine, University of Bristol, Bristol, UK; Department of Psychiatry and Behavioral Neuroscience, University of Chicago, Chicago, USA; Department of Biostatistics and Bioinformatics, Duke University, Durham, NC USA; National Institute for Health and Welfare (THL), Helsinki, Finland; Department of General Practice and Primary Health Care, University of Helsinki, Helsinki, Finland; Unit of General Practice and Primary Health Care, University of Helsinki, Helsinki, Finland; Vasa Central Hospital, Vaasa, Finland; Interfaculty Institute for Genetics and Functional Genomics, University of Greifswald, Greifswald, Germany; Translational Research Institute, University of Queensland Diamantina Institute, Brisbane, Australia; Department of Molecular and Translational Medicine, University of Brescia, Brescia, Italy; Department of Biotechnology, University of Tartu, Tartu, Estonia; Department of Psychiatry, EMGO+ Institute, Neuroscience Campus Amsterdam, VU University Medical Center, Amsterdam, The Netherlands; Institute of Clinical Chemistry and Laboratory Medicine, University Medicine Greifswald, Greifswald, Germany; Wellcome Trust Sanger Institute, Wellcome Trust Genome Campus, Hinxton, Cambridge, UK; Institute for Molecular Medicine Finland (FIMM), University of Helsinki, Helsinki, Finland; Department of Public Health, Faculty of Medicine, University of Split, Split, Croatia; Department of Clinical Physiology and Nuclear Medicine, Turku University Hospital, Turku, Finland; Research Centre of Applied and Preventive Cardiovascular Medicine, University of Turku, Turku, Finland; Department of Psychological & Brain Sciences, Indiana University, Bloomington, IN USA; Department of Psychological Sciences and Missouri Alcoholism Research Center, University of Missouri, Columbia, MO USA; Alzheimer Scotland Dementia Research Centre, University of Edinburgh, Edinburgh, UK; School of Oral and Dental Sciences, University of Bristol, Bristol, UK; School of Experimental Psychology, University of Bristol, Bristol, UK; College of Medicine, Florida State University, Tallahassee, FL USA; Department for Health Evidence, Radboud University Medical Center, Nijmegen, The Netherlands; Department of Public Health and Primary Care, Trinity College Dublin, Dublin, Ireland; Scottish Family Health Study, A Collaboration Between the University Medical Schools and NHS, Aberdeen, Dundee, Edinburgh and Glasgow, UK; Medical Genetics Section, Centre for Genomics and Experimental Medicine, Institute of Genetics and Molecular Medicine, Western General Hospital, The University of Edinburgh, Edinburgh, UK; Department of Psychiatry and Psychotherapy, HELIOS Hospital Stralsund, Stralsund, Germany; Laboratory of Genetics, National Institute on Aging, National Institutes of Health, Baltimore, MD USA; Institute of Public Health, University of Southern Denmark, Odense, Denmark

**Keywords:** Personality, Phenotype harmonization, Common genetic variants, Imputation, Polygenic risk

## Abstract

Extraversion is a relatively stable and heritable personality trait associated with numerous psychosocial, lifestyle and health outcomes. Despite its substantial heritability, no genetic variants have been detected in previous genome-wide association (GWA) studies, which may be due to relatively small sample sizes of those studies. Here, we report on a large meta-analysis of GWA studies for extraversion in 63,030 subjects in 29 cohorts. Extraversion item data from multiple personality inventories were harmonized across inventories and cohorts. No genome-wide significant associations were found at the single nucleotide polymorphism (SNP) level but there was one significant hit at the gene level for a long non-coding RNA site (LOC101928162). Genome-wide complex trait analysis in two large cohorts showed that the additive variance explained by common SNPs was not significantly different from zero, but polygenic risk scores, weighted using linkage information, significantly predicted extraversion scores in an independent cohort. These results show that extraversion is a highly polygenic personality trait, with an architecture possibly different from other complex human traits, including other personality traits. Future studies are required to further determine which genetic variants, by what modes of gene action, constitute the heritable nature of extraversion.

**Electronic supplementary material:**

The online version of this article (doi:10.1007/s10519-015-9735-5) contains supplementary material, which is available to authorized users.

## Introduction

Extraversion is a personality trait characterized by the tendency to experience positive emotions, to be active and feel energetic, to be talkative and to enjoy social interactions. Extraversion is associated with numerous psychosocial, lifestyle and health outcomes, such as academic and job performance, well-being, obesity, substance use, physical activity, bipolar disorder, borderline personality disorder, Alzheimer’s disease, and longevity (De Moor et al. 2006, 2011; Distel et al. 2009a; Furnham et al. 2013; Judge et al. 2013; Middeldorp et al. 2011; Rhodes and Smith 2006; Sutin et al. 2011; Terracciano et al. 2008; Terracciano et al. 2014; Weiss et al. 2008).

Extraversion can be measured with multiple inventories that have been developed as part of different personality theories. For example, extraversion is one of the five personality domains as assessed with the Neuroticism–Extraversion–Openness to Experience (NEO) personality inventories (Costa and McCrae 1992). Extraversion is also included in Eysenck’s three-dimensional theory of personality (Eysenck and Eysenck 1964, 1975; Eysenck et al. 1985). In Cloninger’s theory on temperaments and characters (Cloninger 1987; Cloninger et al. 1993), Harm Avoidance, Novelty Seeking and Reward Dependence are related to extraversion (De Fruyt et al. 2000). Tellegen’s personality theory posits the higher order domain of Positive Emotionality (Patrick et al. 2002), which resembles and is highly correlated with extraversion (Church 1994).

We showed recently, by performing an Item Response Theory (IRT) analysis using test linking (Kolen and Brennan 2004), that item data on Extraversion, Reward dependence and Positive Emotionality can be harmonized to broadly assess the same underlying extraversion construct (van den Berg et al. 2014). This harmonization was performed in over 160,000 individuals from 23 cohorts participating in the Genetics of Personality Consortium (GPC). Briefly, harmonization was carried out in each cohort separately by first fitting an IRT model to data from individuals who had completed at least two different personality questionnaires. Next, based on calibrated item parameters, personality scores were estimated based on all available data for each individual, irrespective of what personality questionnaire was used. The harmonized extraversion phenotype was heritable. A broad-sense heritability of 49 % was estimated, based on a meta-analysis in six twin cohorts that are included in the GPC (29,501 twin pairs), of which 24 % was due to additive genetic variance and 25 % due to non-additive genetic variance. The broad-sense heritability estimate is similar to heritability estimates obtained for extraversion as assessed with single measurement instruments (Bouchard and Loehlin 2001; Distel et al. 2009b; Finkel and McGue 1997; Keller et al. 2005; Rettew et al. 2008; Yamagata et al. 2006). Some evidence for qualitative sex differences in the genetic influences on extraversion was suggested by a genetic correlation in opposite-sex twin pairs of 0.38 (van den Berg et al. 2014). Extraversion becomes more genetically stable during adolescence until it is almost perfectly genetically stable in adulthood (Briley and Tucker-Drob 2014; Kandler 2012), that is, the same genes are responsible for extraversion measured at different ages.

A handful of genome-wide association (GWA) studies for extraversion have been published, aimed at detecting specific single nucleotide polymorphisms (SNPs) that explain part of the heritability. The first GWA study for personality, which focused on the five NEO personality traits, was conducted in 3972 adults (Terracciano et al. 2010). No genome-wide significant SNP associations were found for extraversion, although some interesting associations with *P*-values <10^−5^ were seen with SNPs in two cadherin genes and the brain-derived neurotrophic factor (*BDNF*) gene. A subsequent meta-analysis of GWA results for the NEO personality traits, conducted in 17,375 subjects, also did not yield any genome-wide significant associations for extraversion (De Moor et al. 2012). Two other GWA studies reported a similar lack of genome-wide significance for Cloninger’s temperament scales (Service et al. 2012; Verweij et al. 2010). Interestingly, a study that performed a genetic complex trait analysis (GCTA; Yang et al. 2010) for neuroticism and extraversion in around 12,000 unrelated individuals reported that 12 % (SE = 3 %) of the variance in extraversion was explained by common SNPs of additive effect (Vinkhuyzen et al. 2012). Taken together, the results from twin and genome-wide studies suggest that common SNPs of additive effect are important, that genetic non-additivity may play a role, and that large sample sizes are likely to be required to identify specific variants.

In this paper, we report the results of the largest meta-analysis of GWA results for extraversion so far, carried out in 29 cohorts that participate in the GPC. A total of 63,030 subjects with harmonized extraversion and genome-wide genotype data were included in the meta-analysis. A 30th cohort was used for replication. In this consortium we reported earlier on a genome-wide significant hit for neuroticism (De Moor et al. 2015), indicating that we may begin to analyze data from sufficiently large samples, to obtain the first significant findings from GWA studies for personality. In addition to meta-analysis of GWA results, we computed weighted polygenic scores in an independent cohort and associated them with extraversion, and estimated variance explained by SNPs in two large cohorts.

## Materials and methods

### Cohorts

The full meta-analysis was performed on 63,030 subjects from 29 discovery cohorts. All samples were of European origin. Twenty-one cohorts were from Europe, six from the United States and two from Australia. Sample sizes of the individual cohorts ranged from 177 to 7210 subjects. Please note that some cohorts were also part of previously published GWA studies on extraversion. The Generation Scotland: Scottish Family Health Study (GS:SFHS) cohort was included as a replication sample (9,783 subjects). A brief overview of all cohorts is provided in Table 1. A description of each individual cohort is found in the Supplementary materials and methods (see also De Moor et al. 2015).

**Table 1:** Overview of 29 discovery cohorts and 1 replication cohort of the Genetics of Personality Consortium

	Cohort	# Subjects^a^	# SNPs^b^
1	ALSPAC	4705	6,454,153
2	BLSA	820	4,989,411
3	BRESCIA	177	3,549,919
4	CHICAGO	311	3,755,416
5	CILENTO	627	1,123,089
6	COGA	647	5,127,101
7	COGEND	1279	5,932,838
8	EGCUT	1184	5,574,695
9	ERF	2300	5,142,865
10	FTC EPI	567	4,870,096
11	FTC NEO	813	5,092,018
12	HBCS	1456	5,612,790
13	CROATIA-Korcula	808	5,094,034
14	LBC1921	437	4,363,611
15	LBC1936	952	5,168,754
16	MCTFR	7099	6,569,999
17	MGS	2101	5,900,898
18	NBS	1832	5,603,447
19	NESDA	2227	4,707,569
20	NTR	6416	5,339,798
21	ORCADES	1650	4,265,590
22	PAGES	476	4,547,293
23	QIMR adolescents	2842	5,957,064
24	QIMR adults	7210	6,343,920
25	SardiNIA	4332	6,291,135
26	SHIP	2213	5,913,428
27	STR	4903	6,519,094
28	CROATIA-Vis	909	5,327,671
29	YFS	1737	5,914,679
	Total	63,030	7,460,147
30	GS:SFHS	9783	74

*NA* Not Applicable for replication cohort because only top hits were sought to replicate

^a^Number of subjects with valid latent score for Extraversion and SNP data (after imputation and cleaning)

^b^Number of SNPs (after imputation and cleaning) with valid association results that entered the meta-analysis

### Phenotyping

A harmonized latent extraversion score was estimated for all participants in all 29 cohorts that were included in the GWA meta-analysis. This score was based on all available extraversion item data for each individual (for a detailed description see van den Berg et al. 2014). Extraversion item data came from the extraversion scales of the NEO Personality Inventory, the NEO Five Factor Inventory, the 50-item Big-Five version of the International Personality Item Pool inventory, the Eysenck Personality Questionnaire and the Eysenck Personality Inventory, from the Reward Dependence scale of the Cloninger’s Tridimensional Personality Questionnaire, and from the Positive Emotionality scale of the Multidimensional Personality Questionnaire (see van den Berg et al. 2014 and Supplementary materials and methods). In the GS:SFHS cohort that was included for replication of top signals, extraversion was based on the summed score of the extraversion scale of the EPQ Revised Short Form.

### Genotyping and imputation

Genotyping in all cohorts was carried out on Illumina or Affymetrix platforms, after which quality control (QC) was performed, followed by imputation of genotypes. QC of genotype data was performed in each cohort separately, with comparable but cohort specific criteria. Standard QC checks included tests of European ancestry, sex inconsistencies, Mendelian errors, and high genome-wide homozygosity. Checks for relatedness were conducted in those cohorts that aimed to include unrelated individuals only. Other checks of genotype data were based on minor allele frequencies (MAF), SNP call rate (% of subjects with missing genotypes per SNP), sample call rate (% of missing SNPs per subject) and Hardy–Weinberg Equilibrium (HWE). Genotype data were imputed using the 1000Genomes phase 1 version 3 (build37, hg19) reference panel with standard software packages such as IMPUTE, MACH, or Minimac, see Supplementary Table 1.

### Statistical analyses

#### GWA analysis per cohort

GWA analyses were conducted independently in each cohort. Since the cohorts used different research designs (case–control, population twin studies, extended pedigrees, etc.), GWA methods were optimized for each cohort. Extraversion scores were regressed on each SNP under an additive model, with sex and age included as covariates. Covariates such as ancestry Principal Components (PCs) were added if deemed necessary for a particular cohort. In all analyses, the uncertainty of the imputed genotypes was taken into account, either using dosage scores or mixtures of distributions. In those cohorts that included related individuals, the dependency among participants was accounted for using cohort-specific methods. Standard software packages for GWA analyses were used (see Supplementary Table 1).

#### Meta-analysis of GWA results across cohorts

A meta-analysis of the GWA results was conducted with the weighted inverse variance method in METAL (http://www.sph.umich.edu/csg/abecasis/metal/index.html). Excluded from meta-analysis were poorly imputed SNPs (*r*^2^ < 0.30 or proper_info < 0.40) and SNPs with low MAF (MAF < √(5/N), which corresponds to less than 5 estimated individuals in the least frequent genotype group, under the assumption of HWE). This resulted in a total number of 7,460,147 unique SNPs in the final meta-analysis (with 1.1–6.6 M SNPs across cohorts). For 2182 SNPs, SNP locations could not be matched with rs names. For an additional 516,362 SNPS, results were based on one cohort only and therefore left out of the analysis, so that the results are based on 6,941,603 SNPs. Genomic control inflation factors (lambda), Manhattan plots and quantile–quantile plots per cohort are provided in Supplementary Table 2 and Supplementary Figs. 1, 2. A *P* value of 5 × 10^−8^ was used as the threshold for genome-wide significance.

The meta-analysis results (*P*-values per SNP) were used as the input to compute *P*-values at the gene level. We performed these analyses in KGG (Li et al. 2012). A *P*-value of 2.87 × 10^−6^ was used as the threshold for genome-wide significance in these gene-wide analyses, based on controlling for the false-discovery rate (Benjamini and Hochberg 1995).

All GWAS SNP top hits with a *P*-value smaller than 1 × 10^−5^ were selected for replication in the GS:SFHS cohort.

#### Polygenic risk score analysis

Additional analyses were conducted to test whether extraversion could be predicted in an independent target cohort based on the GWA meta-analysis results. The target cohort was the Netherlands Twin Register (NTR) cohort (8648 subjects). Polygenic risk scores for this cohort were estimated using LDpred (Vilhjalmsson et al. 2015) that takes into account linkage disequilibrium among the SNPs. The estimation was based on a GWA meta-analysis in which the NTR and NESDA cohorts were excluded (further referred to as the discovery set). With the LD-corrected polygenic risk scores, generalized estimating equation (GEE) modeling was applied to test whether the polygenic risk scores predicted extraversion in the target cohort. The covariates age, sex and ten PCs were included as fixed effects in the model. The model also included a random intercept with family number as the cluster variable, to account for dependency among family members. Outliers on the PCs, including ethnic outliers, were excluded from the analysis.

#### Variance explained by SNPs

In the NTR cohort and the QIMR Berghofer Medical Research Institute (QIMR) adult cohort (see also Supplementary materials and methods), GCTA software (Visscher et al. 2010; Yang et al. 2010) was used to estimate the proportion of variance in extraversion that can be explained by common SNPs of additive effect. In the NTR, this analysis was carried out in a set of 3597 unrelated individuals and in the QIMR adult cohort this was done in 3369 unrelated individuals (in each cohort one member per family was selected with harmonized extraversion and genome-wide SNP data). GCTA analysis was based on best guess genotypes obtained in PLINK using a threshold of a maximum genotype probability >0.70, and additionally filtering on r-squared >0.80. Next, in estimating the GRM matrix in the GCTA software, SNPs with MAF <0.05 were excluded. The additive genetic relationship matrices (GRM) estimated based on SNPs for all individuals formed the basis to estimate the proportion of phenotypic variance explained by SNPs in the NTR and QIMR cohorts. In other words, it was determined to what extent phenotypic similarity between individuals corresponds to genetic similarity (at the SNP level). For both NTR and QIMR, sex, age and a set of population-specific PCs were included as covariates.

## Results

### Meta-analysis of GWA results

**Fig. 1:**
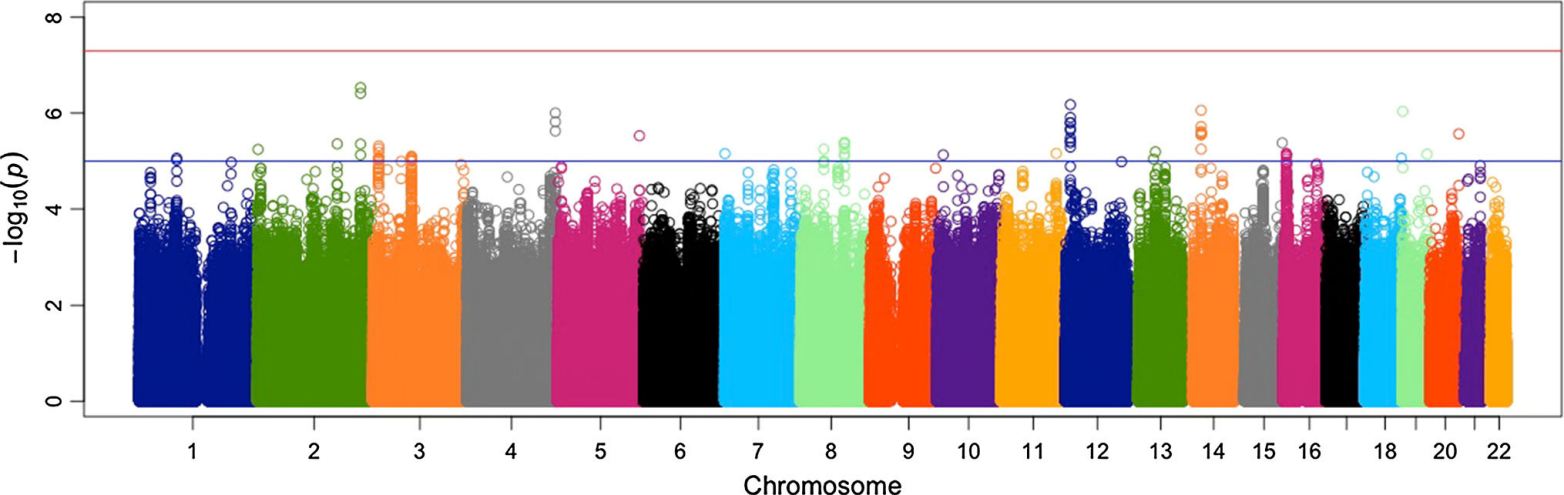
Manhattan plot for meta-analysis results of 29 discovery cohorts for extraversion in the Genetics of Personality Consortium

**Fig. 2:**
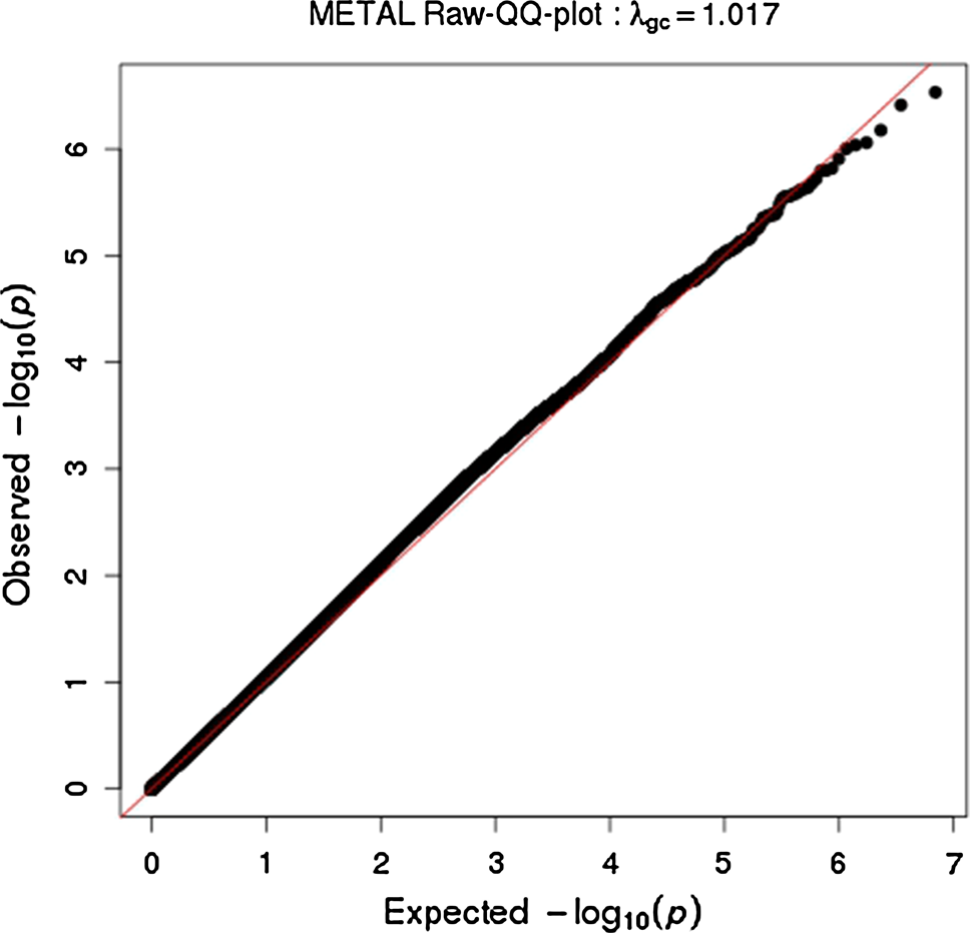
Quantile-Quantile plots for meta-analysis results of 29 discovery cohorts for extraversion in the Genetics of Personality Consortium

Meta-analysis of GWA results across the 29 discovery cohorts did not yield genome-wide significant SNPs associated with extraversion. The lowest *P*-value observed was 2.9 × 10^−7^ for a SNP located on chromosome 2. There were 74 SNPs with *P*-values <1 × 10^−5^. The Manhattan and quantile–quantile plots are provided in Figs. 1 and 2. A list with the top five SNPs is given in Table 2. A list with all SNPs that reached the level of suggestive genome-wide significance (*P* < 1 × 10^−5^) is found in Supplementary Table 3. The results of all SNPs can be downloaded from www.tweelingenregister.org/GPC. A gene-based test showed one significant hit for *LOC101928162*, a long non-coding RNA site, *P* = 2.87 × 10^−6^. A list with the top five genes from the gene-based analysis is provided in Table 3. Supplementary Table 4 provides the top 30 genes. Among the top 30 genes was Brain-Derived Neurotrophic Factor (*BDNF*, *P* = 0.0003), a gene also implicated, though not genome-wide significant, in Terracciano et al. (2010), as was the BDNF anti-sense RNA gene (*P* = 0.0001).

**Table 2:** Top SNPs from the meta-analysis of GWA results in 29 discovery cohorts for extraversion, and their replication in the GS:SFHS cohort, in the Genetics of Personality Consortium

SNP	Chr_BP	Alleles	Closest gene	Discovery results			Replication results		
				Effect	SE	*P*-value	Effect	SE	*P*-value
rs2024488	2_217662968	A/G	*LOC101928250*	−0.0303	0.0059	2.939 x 10^−7^	0.0285	0.0164	0.08244
rs2712162	2_217661788	T/C	*LOC101928250*	−0.0300	0.0059	3.872 x 10^−7^	0.0278	0.0164	0.08947
rs797182	12_10900487	A/G	<NA>	−0.0277	0.0056	6.673 x 10^−7^	−0.0135	0.0153	0.37721
rs8010306	14_37150160	A/G	*SLC25A21*	0.0629	0.0128	8.730 x 10^−7^	0.0180	0.0314	0.56650
rs117292860	19_2227621	A/C	*DOT1L*	0.0553	0.0113	9.191 x 10^−7^	−0.0350	0.0239	0.14368

**Table 3:** Top genes from the meta-analysis of GWA results in 29 discovery cohorts for Extraversion in the Genetics of Personality Consortium

Gene	Full gene name	Pathways	*P*-value
*LOC101928162*	[Long non-coding RNA]	Unknown	0.00000287
*LOC729506*	[Long non-coding RNA]	Unknown	0.00000893
*PLEKHJ1*	Pleckstrin Homology Domain Containing, Family J Member 1	Phospholipid binding, circadian clock functioning	0.0000132
*POU2F3*	POU Class 2 Homeobox 3	Influenza A	0.0000179
*CRTAC1*	Cartilage Acidic Protein 1	Unknown	0.0000297

Results of the follow-up analysis of the top five SNPs in the GS:SFHS cohort can be found in Table 2. Of the top five SNPs, none showed a significant effect. For an overview of the replication results of all top SNPs with *P*-value <1 × 10^−5^ see Supplementary Table 3. Of the 74 SNPs tested in the replication cohort, three SNPs showed nominal evidence of association (*P* < 0.05), which is less than the number expected based on chance alone (0.05 × 74 = 3.7).

### Polygenic risk score analysis

There were 8201 persons individuals with polygenic scores for prediction of extraversion. The LDpred-based genetic risk scores significantly predicted extraversion in the target cohort, *B* = 0.059, *X*^2^(1) = 27.30, *P* < 0.001.

### Variance explained by SNPs

In the NTR cohort, an estimated 5.0 % (SE = 7.2) of the variance in extraversion was explained by all SNPs, but this estimate was not significantly different from zero (*P* = 0.24). In the QIMR cohort, 0.0001 % (SE = 15) of the variance was explained by SNPs (*P* = 0.46).

## Discussion

This study assessed the influence of common genetic variants on extraversion in 63,030 individuals from 29 cohorts in the GPC. First, a meta-analysis of GWA analyses across 29 discovery cohorts showed no genome-wide significant SNPs. Top SNPs detected in the meta-analysis of GWA results in the discovery phase were not replicated in the GS:SFHS cohort. The SNPs with lowest *P*-values have no previously reported relationship with personality, psychopathology or brain functioning. Polygenic risk scores based on the meta-analysis results predicted extraversion in an independent data set. SNP-based heritabilities for extraversion were not significantly different from zero in two large cohorts of the GPC.

Although there were no genome-wide significant results for individual SNPs, in the gene-based analysis, there was a significant hit for one locus, *LOC101928162*. This is long noncoding RNA site whose function remains elusive. Interestingly, among the top 30 genes were genes previously implicated in extraversion or in psychiatric disorders associated with extraversion. The low *P*-value for *CRTAC1* (*P* = 2.97 x 10^−5^), harks back to an interesting extraversion SNP (rs7088779) in a previous GWAS on personality (Amin et al. 2013) that is located between *CRTAC1* and *C10orf28*. *RELN* (*P* = 5.69 x 10^−5^) has been reported to increase the risk for schizophrenia and bipolar disorder (Kuang et al. 2011; Ovadia and Shifman 2011), while *ADAM12* (7.65 x 10^−5^) was previously found to be involved in schizophrenia (Farkas et al. 2010), and bipolar disorder treatment (Nadri et al. 2007). The *BDNF* gene was also implicated in a previous extraversion GWAS (Terracciano et al. 2010), though not genome-wide significant. Liu et al. (2005) reported a trend towards association of *BDNF* variants with substance abuse, Jiao et al. (2011) reported an association with obesity, and Lang et al. (2007) and Beuten et al. (2005) reported associations with smoking behavior. As extraversion is known to be associated with lifestyle, obesity and substance abuse, we deem *BDNF* to be an interesting candidate gene for extraversion in future studies, along with *CRTAC*, *ADAM12* and *RELN*.

With the current meta-analysis we more than tripled the sample size as compared to the largest previously published meta-analysis for extraversion (De Moor et al. 2012). In contrast to neuroticism, no genome-wide significant SNPs were found. Some have argued (Turkheimer et al. 2014) that the heritability of personality traits represents nonspecific genetic background, which is composed of so many genetic variants with extremely small effect sizes that individually these have no causal biological interpretation. It may be that extraversion differs in this respect from neuroticism. One other difference was indicated from the analyses of the IRT-based extraversion and neuroticism scores: whereas for neuroticism no evidence for genotype x sex interaction was seen (van den Berg et al. 2014), for extraversion there was significant evidence for sex limitation. It also is interesting to note that despite the fact that for extraversion no genome-wide significant findings emerged for single SNPs, we were able to predict extraversion in an independent dataset, based on the polygenic risk cohorts from the discovery set. This indicates that some true signal is entailed in the meta-analysis results.

The results of the polygenic risk score analysis are in contrast with the results from the GCTA analysis, in which no significant proportion of variance explained by SNPs was detected in two large cohorts of the GPC. Our study on neuroticism reported a SNP-based heritability of 15 % (De Moor et al. 2015). The current extraversion GCTA findings are also somewhat at odds with two previous GCTA studies for personality traits. One study focused on neuroticism and extraversion as measured with different instruments in four cohorts, and found on average 12 % explained variance for extraversion, although across cohorts these estimates varied widely (0–27 %) (Vinkhuyzen et al. 2012). Estimates for neuroticism also varied, but were generally lower than for extraversion in this study, with an average of 6 % explained variance. In another study, between 4.2 and 9.9 % of explained variances were found for the four Cloninger temperaments in a combined sample of four cohorts (Verweij et al. 2012). The proportions of variances for Harm Avoidance, Novelty Seeking and Persistence were significant at *P* < 0.05, whereas interestingly the proportion of variance for Reward Dependence was not. It should be noted that both these studies included the QIMR cohort in their analyses, so there is some overlap in subjects across studies. The difference is that in the earlier studies extraversion and reward dependence were based on single personality inventories, while in our study extraversion scores harmonized among different personality inventories were analyzed. What our results and the results in the previous studies have in common though, is that the estimates are considerably smaller than the heritability estimates based on twin studies. Given that about half of the heritability of extraversion consists of non-additive genetic variance (van den Berg et al. 2014), it is not unlikely that this discrepancy is caused by the influence of common variants that interact within loci (dominance) or across loci (epistasis). In addition, the influence of rare variants may be implicated. The relatively limited influence of common additive genetic variation, as well as a previously reported finding that higher levels of inbreeding are associated with less socially desirable personality trait levels, has led to the idea that the genetic variation in personality traits may have been maintained by mutation–selection balance (Verweij et al. 2012), and our results are consistent with this idea.

This study comes with some limitations. Genotyping, QC, and imputation were carried out separately in each cohort. Any difference in procedures may have caused some loss of statistical power to detect SNPs in the meta-analysis. Similarly, extraversion item data were harmonized as much as possible (van den Berg et al. 2014), but the Reward Dependence item data from the TCI were least successfully linked to the extraversion data from the other inventories. This may also have caused some loss in power. Importantly however, it should be noted that by combining genotype and phenotype data across cohorts as performed in this study, a substantial increase in sample size was obtained. It is nontrivial that the gain in power associated with this increase in sample size largely outweighs any potential loss in power due to any remaining genotyping or phenotyping differences across cohorts.

In conclusion, extraversion is a heritable, highly polygenic personality trait with a genetic background that may be qualitatively different from that of other complex behavioral traits. Future studies are required to increase our knowledge of which types of genetic variants, by which modes of gene action, constitute the heritable nature of extraversion. Ultimately, this knowledge can be used to increase our understanding of how extraversion is related to various important psychosocial and health outcomes.

## Electronic supplementary material

Supplementary material 1 (DOCX 1358 kb)

## References

[CR300] Amin N, Hottenga JJ, Hansell NK, Janssens ACJW, de Moor MHM, Madden PAF, Zorkoltseva IV, Penninx BW, Terracciano A, Uda M, Tanaka T, Esko T, Realo A, Ferrucci L, Luciano M, Davies G, Metspalu A, Abecasis GR, Deary IJ, Raikkonen K, Bierut LJ, Costa PT, Saviouk V, Zhu G, Kirichenko AV, Isaacs A, Aulchenko YS, Willemsen G, Heath AC, Pergadia ML, Medland SE, Axenovich TI, de Geus E, Montgomery GW, Wright MJ, Oostra BA, Martin NG, Boomsma DI, van Duijn CM (2013) Refining genome-wide linkage intervals using a meta-analysis of genome-wide association studies identifies loci influencing personality dimensions. Eur J Hum Genet 21(8):876–882 10.1038/ejhg.2012.263 PMC3722675 23211697

[CR1] Benjamini Y, Hochberg Y (1995). Controlling the false discovery rate—a practical and powerful approach to multiple testing. J R Stat Soc Series B-Methodol.

[CR900] Beuten J, Ma JZ, Payne TJ, Dupont RT, Quezada P, Huang WH, Crews KA, Li MD (2005) Significant association of BDNF haplotypes in European-American male smokers but not in European-American female or African-American smokers. Am J Med Genet B 139b(1):73–80 10.1002/ajmg.b.30231 16152573

[CR2] Bouchard TJ, Loehlin JC (2001). Genes, evolution, and personality. Behav Genet.

[CR3] Briley DA, Tucker-Drob EM (2014). Genetic and environmental continuity in personality development: a meta-analysis. Psychol Bull.

[CR4] Church AT (1994). Relating the tellegen and 5-factor models of personality structure. J Pers Soc Psychol.

[CR5] Cloninger CR (1987). A systematic method for clinical description and classification of personality variants—a proposal. Arch Gen Psychiatry.

[CR6] Cloninger CR, Svrakic DM, Przybeck TR (1993). A psychobiological model of temperament and character. Arch Gen Psychiatry.

[CR7] Costa PT, McCrae RR (1992). Professional manual: revised NEO personality inventory (NEO-PI-R) and NEO five-factor-inventory (NEO-FFI).

[CR8] De Fruyt F, Van De Wiele L, Van Heeringen C (2000). Cloninger’s psychobiological model of temperament and character and the five-factor model of personality. Pers Indiv Differ.

[CR9] De Moor MHM, Beem AL, Stubbe JH, Boomsma DI, de Geus EJC (2006). Regular exercise, anxiety, depression and personality: a population-based study. Prev Med.

[CR10] De Moor MHM, Vink JM, van Beek JHDA, Geels LM, Bartels M, de Geus EJC, Willemsen AHM, Boomsma DI (2011) Heritability of problem drinking and the genetic overlap with personality in a general population sample. Front Behav Psychiatr Genet 2:76 10.3389/fgene.2011.00076 PMC3268629 22303371

[CR11] De Moor MH, Costa PT, Terracciano A, Krueger RF, de Geus EJ, Toshiko T, Penninx BW, Esko T, Madden PA, Derringer J, Amin N, Willemsen G, Hottenga JJ, Distel MA, Uda M, Sanna S, Spinhoven P, Hartman CA, Sullivan P, Realo A, Allik J, Heath AC, Pergadia ML, Agrawal A, Lin P, Grucza R, Nutile T, Ciullo M, Rujescu D, Giegling I, Konte B, Widen E, Cousminer DL, Eriksson JG, Palotie A, Peltonen L, Luciano M, Tenesa A, Davies G, Lopez LM, Hansell NK, Medland SE, Ferrucci L, Schlessinger D, Montgomery GW, Wright MJ, Aulchenko YS, Janssens AC, Oostra BA, Metspalu A, Abecasis GR, Deary IJ, Raikkonen K, Bierut LJ, Martin NG, van Duijn CM, Boomsma DI (2012). Meta-analysis of genome-wide association studies for personality. Mol Psychiatry.

[CR12] De Moor MHM, van den Berg SM, Consortium GoP, Boomsma DI (2015). Meta-analysis of genome-wide association studies for neuroticism, and the polygenic association with major depressive disorder. JAMA Psychiatry.

[CR13] Distel MA, De Moor MHM, Boomsma DI (2009). Dutch translation of the Personality Assessment Inventory-Borderline features scale (PAI-BOR): norms, factor structure and reliability. Psychol Gezondh.

[CR14] Distel MA, Trull TJ, Willemsen G, Vink JM, Derom CA, Lynskey MT, Martin NG, Boomsma DI (2009). The Five Factor Model of personality and borderline personality disorder: a genetic analysis of comorbidity. Biol Psychiatry.

[CR15] Eysenck HJ, Eysenck SBG (1964). Eysenck personality inventory.

[CR16] Eysenck HJ, Eysenck SBG (1975). Manual of the eysenck personality questionnaire.

[CR17] Eysenck SBG, Eysenck HJ, Barrett P (1985). A revised version of the psychoticism scale. Pers Indiv Differ.

[CR600] Farkas N, Lendeckel U, Dobrowolny H, Funke S, Steiner J, Keilhoff G, Schmitt A, Bogerts B, Bernstein HG (2010) Reduced density of ADAM 12-immunoreactive oligodendrocytes in the anterior cingulate white matter of patients with schizophrenia. World J Biol Psychiatry 11(3):556–566 10.3109/15622970903497936 20218926

[CR18] Finkel D, McGue M (1997). Sex differences and nonadditivity in heritability of the multidimensional personality questionnaire scales. J Pers Soc Psychol.

[CR19] Furnham A, Nuygards S, Chamorro-Premuzic T (2013). Personality, assessment methods and academic performance. Instr Sci.

[CR200] Jiao H, Arner P, Hoffstedt J, Brodin D, Dubern B, Czernichow S, van’t Hooft F, Axelsson T, Pedersen O, Hansen T, Sorensen TIA, Hebebrand J, Kere J, Dahlman-Wright K, Hamsten A, Clement K, Dahlman I (2011) Genome wide association study identifies KCNMA1 contributing to human obesity. BMC Med Genomics 4:51 10.1186/1755-8794-4-51 PMC3148553 21708048

[CR20] Judge TA, Rodell JB, Klinger RL, Simon LS, Crawford ER (2013). Hierarchical representations of the five-factor model of personality in predicting job performance: integrating three organizing frameworks with two theoretical perspectives. J Appl Psychol.

[CR21] Kandler C (2012). Nature and nurture in personality development: the case of neuroticism and extraversion. Curr Dir Psychol Sci.

[CR22] Keller MC, Coventry WL, Heath AC, Martin NG (2005). Widespread evidence for non-additive genetic variation in Cloninger’s and Eysenck’s personality dimensions using a twin plus sibling design. Behav Genet.

[CR23] Kolen MJ, Brennan RL (2004). Test equating, scaling, and linking: methods and practices.

[CR400] Kuang WJ, Sun RF, Zhu YS, Li SB (2011) A new single-nucleotide mutation (rs362719) of the reelin (RELN) gene associated with schizophrenia in female Chinese Han. Genet Mol Res 10(3):1650–1658 10.4238/vol10-3gmr1343 21863557

[CR800] Lang UE, Sander T, Lohoff FW, Hellweg R, Bajbouj M, Winterer G, Gallinat J (2007) Association of the met66 allele of brain-derived neurotrophic factor (BDNF) with smoking. Psychopharmacology (Berl) 190(4):433–439 10.1007/s00213-006-0647-1 17186223

[CR24] Li MX, Kwan JS, Sham PC (2012). HYST: a hybrid set-based test for genome-wide association studies, with application to protein-protein interaction-based association analysis. Am J Hum Genet.

[CR100] Liu Q-R, Walther D, Drgon T, Polesskaya O, Lesnick TG, Strain KJ, de Andrade M, Bower JH, Maraganore DM, Uhl GR (2005) Human brain derived neurotrophic factor (BDNF) genes, splicing patterns, and assessments of associations with substance abuse and Parkinson’s Disease. Am J Med Genet B Neuropsychiatr Genet 134B(1):93–103 10.1002/ajmg.b.30109 15666411

[CR25] Middeldorp CM, de Moor MH, McGrath LM, Gordon SD, Blackwood DH, Costa PT, Terracciano A, Krueger RF, de Geus EJ, Nyholt, Tanaka T, Esko T, Madden PA, Derringer J, Amin N, Willemsen G, Hottenga JJ, Distel MA, Uda M, Sanna S, Spinhoven P, Hartman CA, Ripke S, Sullivan PF, Realo A, Allik J, Heath AC, Pergadia ML, Agrawal A, Lin P, Grucza RA, Widen E, Cousminer DL, Eriksson JG, Palotie A, Barnett JH, Lee PH, Luciano M, Tenesa A, Davies G, Lopez LM, Hansell NK, Medland SE, Ferrucci L, Schlessinger D, Montgomery GW, Wright MJ, Aulchenko YS, Janssens AC, Oostra BA, Metspalu A, Abecasis GR, Deary IJ, Raikkonen K, Bierut LJ, Martin NG, Wray NR, van Duijn CM, Smoller JW, Penninx BW, Boomsma DI (2011). The genetic association between personality and major depression or bipolar disorder. A polygenic score analysis using genome-wide association data. Transl Psychiatry.

[CR700] Nadri C, Bersudsky Y, Belmaker RH, Agam G (2007) Elevated urinary ADAM12 protein levels in lithium-treated bipolar patients. J Neural Transm 114(4):473–477 10.1007/s00702-006-0586-3 17066252

[CR500] Ovadia G, Shifman S (2011) The genetic variation of RELN expression in schizophrenia and bipolar disorder. PLoS One 6(5):e19955 10.1371/journal.pone.0019955 PMC3095646 21603580

[CR26] Patrick CJ, Curtin JJ, Tellegen A (2002). Development and validation of a brief form of the multidimensional personality questionnaire. Psychol Assess.

[CR27] Rettew DC, Rebollo-Mesa I, Hudziak JJ, Willemsen G, Boomsma DI (2008). Non-additive and additive genetic effects on extraversion in 3314 Dutch adolescent twins and their parents. Behav Genet.

[CR28] Rhodes RE, Smith NEI (2006). Personality correlates of physical activity: a review and meta-analysis. Br J Sports Med.

[CR29] Service SK, Verweij KJ, Lahti J, Congdon E, Ekelund J, Hintsanen M, Raikkonen K, Lehtimaki T, Kahonen M, Widen E, Taanila A, Veijola J, Heath AC, Madden PA, Montgomery GW, Sabatti C, Jarvelin MR, Palotie A, Raitakari O, Viikari J, Martin NG, Eriksson JG, Keltikangas-Jarvinen L, Wray NR, Freimer NB (2012). A genome-wide meta-analysis of association studies of Cloninger’s Temperament Scales. Transl Psychiatry.

[CR30] Sutin AR, Ferrucci L, Zonderman AB, Terracciano A (2011). Personality and obesity across the adult life span. J Pers Soc Psychol.

[CR31] Terracciano A, Lockenhoff CE, Zonderman AB, Ferrucci L, Costa PT (2008). Personality predictors of longevity: activity, emotional stability, and conscientiousness. Psychosom Med.

[CR32] Terracciano A, Sanna S, Uda M, Deiana B, Usala G, Busonero F, Maschio A, Scally M, Patriciu N, Chen WM, Distel MA, Slagboom EP, Boomsma DI, Villafuerte S, Sliwerska E, Burmeister M, Amin N, Janssens ACJW, van Duijn CM, Schlessinger D, Abecasis GR, Costa PT (2010). Genome-wide association scan for five major dimensions of personality. Mol Psychiatry.

[CR33] Terracciano A, Sutin AR, An Y, O’Brien RJ, Ferrucci L, Zonderman AB, Resnick SM (2014). Personality and risk of Alzheimer’s disease: new data and meta-analysis. Alzheimer’s Dement J Alzheimer’s Assoc.

[CR34] Turkheimer E, Pettersson E, Horn EE (2014). A phenotypic null hypothesis for the genetics of personality. Annu Rev Psychol.

[CR35] van den Berg SM, de Moor MH, McGue M, Pettersson E, Terracciano A, Verweij KJ, Amin N, Derringer J, Esko T, van Grootheest G, Hansell NK, Huffman J, Konte B, Lahti J, Luciano M, Matteson LK, Viktorin A, Wouda J, Agrawal A, Allik J, Bierut L, Broms U, Campbell H, Smith GD, Eriksson JG, Ferrucci L, Franke B, Fox JP, de Geus EJ, Giegling I, Gow AJ, Grucza R, Hartmann AM, Heath AC, Heikkila K, Iacono WG, Janzing J, Jokela M, Kiemeney L, Lehtimaki T, Madden PA, Magnusson PK, Northstone K, Nutile T, Ouwens KG, Palotie A, Pattie A, Pesonen AK, Polasek O, Pulkkinen L, Pulkki-Raback L, Raitakari OT, Realo A, Rose RJ, Ruggiero D, Seppala I, Slutske WS, Smyth DC, Sorice R, Starr JM, Sutin AR, Tanaka T, Verhagen J, Vermeulen S, Vuoksimaa E, Widen E, Willemsen G, Wright MJ, Zgaga L, Rujescu D, Metspalu A, Wilson JF, Ciullo M, Hayward C, Rudan I, Deary IJ, Raikkonen K, Arias Vasquez A, Costa PT, Keltikangas-Jarvinen L, van Duijn CM, Penninx BW, Krueger RF, Evans DM, Kaprio J, Pedersen NL, Martin NG, Boomsma DI (2014). Harmonization of Neuroticism and Extraversion phenotypes across inventories and cohorts in the Genetics of Personality Consortium: an application of Item Response Theory. Behav Genet.

[CR36] Verweij KJ, Zietsch BP, Medland SE, Gordon SD, Benyamin B, Nyholt DR, McEvoy BP, Sullivan PF, Heath AC, Madden PA, Henders AK, Montgomery GW, Martin NG, Wray NR (2010). A genome-wide association study of Cloninger’s temperament scales: implications for the evolutionary genetics of personality. Biol Psychol.

[CR37] Verweij KJ, Yang J, Lahti J, Veijola J, Hintsanen M, Pulkki-Raback L, Heinonen K, Pouta A, Pesonen AK, Widen E, Taanila A, Isohanni M, Miettunen J, Palotie A, Penke L, Service SK, Heath AC, Montgomery GW, Raitakari O, Kahonen M, Viikari J, Raikkonen K, Eriksson JG, Keltikangas-Jarvinen L, Lehtimaki T, Martin NG, Jarvelin, Visscher PM, Keller MC, Zietsch BP (2012). Maintenance of genetic variation in human personality: testing evolutionary models by estimating heritability due to common causal variants and investigating the effect of distant inbreeding. Evolution.

[CR38] Vilhjalmsson B, Yang J, Finucane HK, Gusev A, Lindstrom S, Ripke S, Genovese G, Loh P-R, Bhatia G, Do R, Hayeck T, Won H-H, Genomics Consortium SWGotP, Variants in Breast Cancer study tDB, Risk of I, Kathiresan S, Pato M, Pato C, Tamimi R, Stahl E, Zaitlen N, Pasaniuc B, Schierup M, De Jager P, Patsopoulos N, McCarroll SA, Daly M, Purcell S, Chasman D, Neale B, Goddard M, Visscher PM, Kraft P, Patterson NJ, Price AL (2015) Modeling Linkage Disequilibrium Increases Accuracy of Polygenic Risk Scores. bioRxiv. doi:10.1101/015859

[CR39] Vinkhuyzen AA, Pedersen NL, Yang J, Lee SH, Magnusson PK, Iacono WG, McGue M, Madden PA, Heath AC, Luciano M, Payton A, Horan M, Ollier W, Pendleton N, Deary IJ, Montgomery GW, Martin NG, Visscher PM, Wray NR (2012). Common SNPs explain some of the variation in the personality dimensions of neuroticism and extraversion. Transl Psychiatry.

[CR40] Visscher PM, Yang J, Goddard ME (2010). A commentary on ‘Common SNPs explain a large proportion of the heritability for human height’ by Yang et al. (2010). Twin Res Human Genet.

[CR41] Weiss A, Bates TC, Luciano M (2008). Happiness is a personal(ity) thing—the genetics of personality and well-being in a representative sample. Psychol Sci.

[CR42] Yamagata S, Suzuki A, Ando J, Ono Y, Kijima N, Yoshimura K, Ostendorf F, Angleitner A, Riemann R, Spinath FM, Livesley WJ, Jang KL (2006). Is the genetic structure of human personality universal? A cross-cultural twin study from North America, Europe, and Asia. J Pers Soc Psychol.

[CR43] Yang J, Benyamin B, McEvoy BP, Gordon S, Henders AK, Nyholt DR, Madden PA, Heath AC, Martin NG, Montgomery GW, Goddard ME, Visscher PM (2010). Common SNPs explain a large proportion of the heritability for human height. Nat Genet.

