# Supplementary material for: Meta-analysis of Genome-Wide Association Studies for Extraversion: Findings from the Genetics of Personality Consortium
Source: Behav Genet. 2015 Sep 11;46:170–82. doi: 10.1007/s10519-015-9735-5 (PMC4751159; doi:10.1007/s10519-015-9735-5)
Supplement: Supplementary file 1 — Supplementary material 1 (DOCX 1358 kb) [file 10519_2015_9735_MOESM1_ESM.docx]

**Supplementary Materials Online, belonging to**

**Van den Berg et al. *Meta-analysis of genome-wide association studies for extraversion: Findings from the Genetics of Personality Consortium.* Submitted for publication.**

Table of contents

Supplementary materials and methods p.1

Cohorts p.1

Personality assessment p.15

Supplementary Table 1 p.16

Supplementary Table 2 p.22

Supplementary Table 3 p.23

Supplementary Table 4 p.28

Supplementary Figure 1 p.30

Supplementary Figure 2 p.45

**Supplementary materials and methods**

**Cohorts**

Approval by local institutional review boards was obtained in all studies and informed consent was obtained from all participants.

*1. ALSPAC*[*^1^*](#_ENREF_1) *— United Kingdom.* The Avon Longitudinal Study of Parents and their Children (ALSPAC) is a longitudinal population-based birth cohort that recruited pregnant women residing in Avon, UK, with an expected delivery date between 1st April 1991 and 31st December 1992. 14,541 pregnant women were initially enrolled with 14,062 children born. Biological samples including DNA have been collected for 10,121 of the children from this cohort. Ethical approval was obtained from the ALSPAC Law and Ethics committee and relevant local ethics committees, and all parents provided written informed consent. IPIP data was available for 6,076 children (3,099 females; 51.0%). Mean age was 13.8 years (SD=0.21). The data were collected between 2005 and 2006. 4,705 children had both valid genotype and extraversion data. The study website contains details of all the data that is available through a fully searchable data dictionary (http://www.bris.ac.uk/alspac/researchers/data-access/data-dictionary).

*2. BLSA*[*^2^*](#_ENREF_2) *— United States of America.* The Baltimore Longitudinal Study of Aging (BLSA) is an ongoing multidisciplinary study of community-dwelling volunteers. For this study, we examined data from 1,917 participants (952 women) of European descent that completed the NEO-PI-R questionnaire. In this sample, mean age was 58.3 years (SD=16.6). The mean age of the males was 56 years (SD=16.7) and of the females 60.7 years (SD=16.3). The data were collected between 1991 and 2010.

*3. BRESCIA – Italy.* The Brescia cohort consisted of 177 unrelated healthy volunteers (89 female and screened for DSM-IV Axis I disorders by expert psychologists using the Mini-International Neuropsychiatric Interview (M.I.N.I.). Only healthy volunteers without a history of drug or alcohol abuse or dependence and without a personal or first-degree family history of psychiatric disorders were enrolled in the study. Subjects who obtained a score lower than 27/30 in the Mini Mental State Examination (M.M.S.E.) were excluded as well. Furthermore, only participants with Italian descent (all four grandparents Italian) could participate. The personality traits were assessed by the Italian version of the TCI, a 240-item, true-false self-report questionnaire. The mean age of the sample was 47.58 years (SD=16.30; women M=47.08, SD=17.31, men M=48.09, SD=15.29). The data were collected between 2007 and 2011.

*4. CHICAGO*[*^3^*](#_ENREF_3) *– United States of America.*

The Chicago cohort comes from a genetic study in which 311 healthy Caucasian adults, aged 18-35 years, were tested for response to an acute dose of *d*-amphetamine. They were recruited from the community and underwent medical and psychiatric screening before the study. Participants completed the Multiphasic Personality Inventory- Brief Form during screening, and they were genotyped using the Affymetrix 6.0 array. The sample consisted of 166 males (mean age 23.6, SD=3.7) and 145 females (mean age 22.6 years, SD=3.2). The data were collected between 2002 and 2010.

*5. CILENTO*[*^4^*](#_ENREF_4)*^,^* [*^5^*](#_ENREF_5) *—Italy.* The Cilento study is a population-based study that includes 2,137 individuals from three isolated populations of South Italy. Data from the NEO-PI-R questionnaire were available for 800 participants representing the final sample. Of this sample, 64.4% were women. The mean age of all participants was 54.6 years (SD=19), of the males 54.6 years (SD=19.2) and of the females 54.6 years (SD=19.5). The data were collected between 2009 and 2011.

*6. EGCUT*[*^6^*](#_ENREF_6) *— Estonia*. The Estonian cohort comes from the population-based biobank of the Estonian Genome Project of University of Tartu (EGCUT). The project is conducted according to the Estonian Gene Research Act and all participants have signed the broad informed consent (www.biobank.ee). In total, 52,000 individuals aged 18 years or older participated in this cohort (33% men, 67% women). General practitioners (GP) and physicians in the hospitals randomly recruited the participants. A Computer-Assisted Personal interview was conducted during 1–2 h at doctors’ offices. Data on demographics, genealogy, educational and occupational history, lifestyle and anthropometric and physiological data were assessed. The personality profile was assayed using NEO-PI-3 questionnaire and was administered to 1,730 participants. In this sample, the age range was 18–88 years (M=42.8 years, SD=16.5). The sample consisted of 740 men (mean age 42 years, SD=16.3) and 991 women (mean age 43.4 years, SD=16.6). The genotyping was carried out in two waves for a random subsamples: 1) 1,183 samples using Illumina Human370CNV array and 2) 120 samples using Illumina OmniExpress array, resulting with an effective sample of 1,303. The data were collected between 2009 and 2012.

*7. ERF*[*^7^*](#_ENREF_7) *— The Netherlands.* The Erasmus Rucphen Family (ERF) study is a family-based study including over 3,000 individuals from an isolated population in the Southwest region of the Netherlands. There were 2,400 individuals for whom both NEO personality and GWA data were available. The personality traits were assessed with the NEO-FFI. The mean age of all participants was 49.3 years (SD=14.9) and women constituted 55.8% of the total sample (M=49.0, SD=15.1, versus in men M=49.6, SD=14.7).

*8+9. FINNISH TWINS EPI AND NEO ^9, 10^ — Finland*. The Finnish twin cohort (FTC) consisted of 1381 genotyped respondents with personality data from two separate twin cohorts: the older Finnish twin cohort consisted of like-sexed twins born before 1958, and FinnTwin12 consisted of twins born in 1983-1987. A total of 567 individuals with available genotyping data (mean age 25.5, SD 4.8; 32.9% women) from the older Finnish twin cohort completed the Eysenck Personality Inventory (EPI; an alternative version of the EPQ) at least once. EPI was assessed in 1975-1976 and for the second time in 1981-1983. Some of the twins from the older Finnish twin cohort were assessed with the NEO-FFI between 2003-2009 as a part of the Nicotine Addiction Genetics (NAG) - Finland study. In addition, NEO-FFI was assessed also in the fourth wave of the FinnTwin12 study in 2005-2009. Combining participants from the NAG and FinnTwin12 studies, the total number of genotyped individuals with the NEO-FFI data was 813 (mean age 30.5, SD 15.3; 49.5% were women). All subjects were genotyped at the Welcome Trust Sanger Institute using the Illumina 670 Custom chip.

*10. HBCS*[*^8-10^*](#_ENREF_8) *— Finland.* The Helsinki Birth Cohort Study (HBCS) is composed of 8,760 individuals born between the years 1934 and 1944 in one of the two main maternity hospitals in Helsinki, Finland. Between 2001 and 2003, a randomly selected sample of 928 men and 1,075 women participated in a clinical follow-up study with a focus on cardiovascular, metabolic and reproductive health, cognitive function and depressive symptoms. In 2004, various psychological phenotypes were assessed, including the NEO and TCI personality dimensions. There were 1 698 participants that completed either the NEO and/or the TCI (55.9% women). The mean age of the subjects was 63.4 years (SD=2.9). The mean age of the men was 63.3 years (SD=2.7) and of the women was 63.5 years (SD=3).

*11. CROATIA-Korcula*[*^11^*](#_ENREF_11) *— Croatia.* This study was performed in the eastern part of the island of Korčula, Croatia between March and December 2007. Healthy volunteers aged 18 and over from the town of Korčula and villages Lumbarda, Žrnovo, and Račišće were invited to the study. There was a total of 969 participants included who had a number of quantitative phenotypic traits measured. The EPQ-R was successfully administered to 810 participants (511 female; 63.1%). The mean age was 55.4 years (SD=13.3; female M=54.5, SD=12.8, male M=56.9, SD=14). The data were collected in 2007.

*12. LBC1921*[*^12^*](#_ENREF_12) *— United Kingdom.* The Lothian Birth Cohort 1921 (LBC1921) study includes 550 individuals born in 1921, most of whom had taken part in the Scottish Mental Survey of 1932. The majority of participants lived independently in the Lothian region (Edinburgh city and surrounding area) of Scotland. Of these, 498 participants were approached to complete the IPIP personality inventory, and 478 participants (283 women; 59.2%) successfully did so. The mean age of both female and male participants was 81.2 (SD=0.3). IPIP data were collected twice: in 2002 and between 2007 and 2008. Measurements from the first IPIP assessment were used for 472 individuals, with second assessment measurements used for six individuals. DNA was extracted from blood samples and genotyped using the Illumina610-Quadv1 conducted by the Genetics Core Laboratory at the Wellcome Trust Clinical Research Facility, Western General Hospital, Scotland. Genotype and phenotype data were available for 437 individuals.

*13. LBC1936*[*^12-14^*](#_ENREF_12) *— United Kingdom.* The Lothian Birth Cohort 1936 (LBC1936) study comprises 1,091 individuals born in 1936, most of whom had taken part in the Scottish Mental Survey of 1947. The majority of participants lived independently in the Lothian region (Edinburgh city and surrounding area) of Scotland. Participants completed NEO-FFI and IPIP personality questionnaires at a mean age of 69.6 years (SD=.82; women M=69.6, SD=.82, men M=69.6, SD=.82); at 72.5 years (SD=.71; women M=72.5, SD=.72, men M=72.4, SD=.82) the IPIP was re-administered. IPIP data collection occurred between 2004 and 2007, and between 2007 and 2010. First wave measurements were used for 963 individuals, and second wave measurements for 69 individuals. DNA was extracted from blood samples and genotyped using the Illumina610-Quadv1 conducted by the Genetics Core Laboratory at the Wellcome Trust Clinical Research Facility, Western General Hospital, Scotland. Genotype and phenotype data were available for 952 individuals.

*14. MCTFR*[*^15^*](#_ENREF_15)*^,^* [*^16^*](#_ENREF_16) *— United States of America.* Data from the Minnesota Center for Twin and Family Research (MCTFR) were collected as part of two different longitudinal studies, the Minnesota Twin Family Study (MTFS) and the Sibling Interaction and Behavior Study (SIBS). The MTFS is a study of reared-together, same-sex twins and their parents, and the SIBS is a study of families of different types (some include adopted offspring). Both parents and offspring completed the Multidimensional Personality Questionnaire (MPQ) at baseline, and only offspring completed it at subsequent follow-ups of approximately 3-year intervals. There were data available for up to 5 follow-ups for offspring in the MTFS and up to 3 for offspring in the SIBS. We selected data from the first assessment with complete data for each individual. The total sample with MPQ data included 9,071 participants (53% female), and of those participants, 7,186 had both IRT-based extraversion scores and usable SNP data. The final sample included 3,346 males (M age = 31.93, SD = 13.96) and 3840 females (mean age = 31.19, SD = 12.97).

*15. MGS – United States of America.* Data derive from the “control” sample of a large schizophrenia study (Molecular Genetics of Schizophrenia (MGS): PI and Collaboration Coordinator, P.V. Gejman). The available sample consisted of unrelated subjects selected by random digit dialing from approximately 60,000 US households. They were screened for psychotic and bipolar disorders but were not excluded for other common psychiatric disorders seen in the general population. The European American sample (53% female) were adults with a mean age of 50 (SD=16.4) at assessment (Sanders, 2010). Subjects completed an on-line psychiatric screening interview that included the short form of the Eysenck Personality Questionnaire (EPQ-SF). The data were obtained with permission from dbGaP (Database of Genotypes and Phenotypes, http://www.ncbi.nlm.nih.gov/gap, Study Accessions: phs000021.v3.p2 (“GAIN”) and phs000167.v1.p1 “nonGAIN”). Data from the GAIN and nonGAIN European American subjects were combined for the current analyses (N=2,806).

*16. NBS*[*^17^*](#_ENREF_17) *— The Netherlands.* In 2000 a study was initiated among the inhabitants of the municipality of Nijmegen by different departments of the Radboud University Nijmegen Medical Centre to research the question what the prevalence of certain risk factors, chronic diseases and genetic variations in the general population are. As a part of this study, the EPQ-R was administered to 1,832 participants. From this sample, 1,823 participants (921 female; 50.5%) completed the test. The mean age of these participants was 61.5 (SD=10.3; female M=56.7, SD=10.8, male M=66.3, SD=7).

*17. NESDA*[*^18^*](#_ENREF_18) *— The Netherlands.* The NESDA data for the present study were drawn from the Netherlands Study of Depression and Anxiety[^18^](#_ENREF_18), an ongoing longitudinal cohort study aimed at examining the long-term course of depressive and anxiety disorders in different health care settings and phases of illness. A total of 2,981 respondents were recruited from primary care (n=1,610), specialized mental health care (n=807) and the community (n=564), including healthy controls, respondents with subthreshold symptoms and those with an anxiety and/or depressive disorder. The NEO-FFI was successfully administered to 2,961 participants (1,979 female; 66.8%). The mean age was 41.9 years (SD=13.1; female M=41.1, SD=13.1, male M=43.4, SD=12.9). Baseline data were collected between 2004 and 2007. The NEO-FFI was administered twice, at baseline and two years later. For the NESDA sample, contrary to the other studies with repeated measure data of personality, we first selected the least recent item data. For NESDA, this strategy was deemed most suitable because the first measurement represented the baseline measurement for NESDA which had an overpresentation of MDD cases. So, we choose to measure personality at the time point at which MDD was least prevalent.

*18. NTR*[*^19^*](#_ENREF_19)*^,^* [*^20^*](#_ENREF_20) *— The Netherlands*. Data on personality in the Netherlands Twin Register (NTR) were collected as part of a longitudinal study on health, personality and lifestyle in adolescent and adult twins and their relatives (i.e., their non-twin siblings, parents, spouses and children). Eight waves of data collection have been completed (in 1991, 1993, 1995, 1997, 2000, 2002, 2004 and 2009). Twins were invited to participate at all time points, while the parents and siblings could participate on a maximum of 6 time points, spouses on 4 time points and adult children of twins and siblings on 2 time points. The ABV was administered five times in 1991, 1993, 1997, 2000 and 2002, and the NEO-FFI was assessed twice in 2004 and 2009. Of the 31,694 individuals who participated at least once in one of these seven waves, there were 31,259 individuals (58.7% female) with valid personality data (at least one Extraversion item was available on at least one time point). For the analysis in this study, we selected for each individual the ABV item data of the latest time point and the NEO item data of the earliest time point. This ensured that for each individual with data on both the ABV and NEO, the times of measurement were as close as possible. For 21,146 individuals there were NEO data available (of which from 14,880 individuals data came from the 2004 survey and from 6 266 individuals data came from the 2009 survey). For the ABV, data of 6,778 individuals came from survey 2002, 1,803 from 2000, 5,088 from the 1997, 2 208 from 1993, and 2,939 from 1991 (in total 18,816 individuals with ABV data). The mean age of the participants was 37.2 years (SD=15.3) across assessments. For the GWAS analysis we selected all participants with GWAS data, allowing first-degree relatives (e.g. sibling pairs and parent-offspring pairs), because a genetic relatedness of 0.5 can be accounted for in the GWAS analysis in PLINK by using the –family option. This led to the inclusion of 6,416 participants for the GWAS analysis. For the GCTA analysis, unrelated participants are required. Hence, we randomly selected one individual per family, yielding 3,597 individuals for the GCTA analysis. In the polygenic risk score analysis where we predicted Extraversion in the NTR cohort, we were able to deal with any degree of genetic relatedness by applying linear mixed modeling. Therefore, we included all subjects with personality and GWAS data, yielding 8,648 individuals in the analysis.

*19. ORCADES*[*^21^*](#_ENREF_21) *— United Kingdom.* The Orkney Complex Disease Study (ORCADES) is a genetic epidemiology study based in an isolated population in the north of Scotland. It aims to discover the genes and variants in them that influence the risk of common, complex diseases such as diabetes, osteoporosis, stroke, heart disease, myopia, glaucoma, chronic kidney and lung disease. As a part of this study, the EPQ-R was administered to 1650 participants and all participants completed the test. The mean age of these participants was 56.8 (SD=13.8; women M=56.5, SD=13.9, men M=57, SD=13.8). The data were collected between 2007 and 2011.

*20. PAGES — Germany.* In this German cohort, healthy control participants were randomly selected from the general population of Munich, Germany, and contacted by mail. Several screenings were conducted before the volunteers were enrolled in the study. These included screening of medical and psychiatric disorders (in particular psychotic disorders) in the participants and their first-degree relatives by phone and interview and screening for central nervous system and cognitive impairment by neurological examination and cognitive testing. Furthermore, only participants with German descent (all four grandparents German) could participate. In the resulting sample, a large battery of personality questionnaires was administered as well as data on life events and traumatic events. Data on the NEO-PI-R and TCI were analyzed for the current study. There were 476 individuals (55.7% women) with valid personality data. The mean age of the sample was 45.9 years (SD=15,4; women M=43.4, SD=15,3, men M=49, SD=15,3). The data were collected between 1998 and 2006.

*21. QIMR adolescents — Australia* Personality data collected between 1992 and 2011 were available for 4,100 adolescents and young adults (51.5% female), of which 2,842 (48.6% female) were genotyped. Participants included in the current study (with both phenotypic and genotypic data) ranged in age from 9 to 25 years (M=14.4, SD=2.3). The sample comprised twin pairs (and their siblings) recruited as adolescents as part of the overarching Brisbane Adolescent Twin Study[^22^](#_ENREF_22) conducted at the QIMR Berghofer Medical Research Institute (QIMR). Recruitment was mostly through primary and secondary schools in south-east Queensland for studies of melanocytic naevi (moles).[^23^](#_ENREF_23) JEPQ and/or NEO personality data (NEO-PI-R or NEO-FFI) were collected as part of the melanocytic naevi study (1992-ongoing), the cognition study (in-person testing, 1996-2012)[^22^](#_ENREF_22), a health and well-being study (a mail/phone study, 2002-2003)[^22^](#_ENREF_22), and a study of borderline personality disorder (online/paper survey, 2003–2006)[^24^](#_ENREF_24). JEPQ data were available at 3 time points, NEO-PI-R data at 1 time point, and NEO-FFI data at 2 time points. Data from the first time point (majority of sample) was retained for both JEPQ and NEO, with the NEO-PI-R given preference over the shorter NEO-FFI. DNA samples were collected in accordance with standard protocols and were genotyped on the Illumina 610 quad.[^25^](#_ENREF_25)

*22. QIMR adults — Australia* Personality data were available for a large Australian adult sample comprising 26,680 individuals (57.1% female), of which 7,201 (38.3% female) were genotyped. Participants included in the current study (with both phenotypic and genotypic data) ranged in age from 17 to 85 years (M=38.1, SD=12.6). Data were collected between 1980 and 2007 in various twin family studies conducted at QIMR.

NEO personality data (NEO-PI-R or NEO-FFI) were collected from a series of studies conducted collaboratively by Nick Martin and Andrew Heath between 2001 and 2006[^26^](#_ENREF_26)^,^ [^27^](#_ENREF_27)^,^ [^24^](#_ENREF_24). The EPQ data were obtained from the following sources: (a) The Canberra study (1980-1981)[^28^](#_ENREF_28): twins drawn from the Australian Twin Registry and born prior to 1964 (‘Cohort 1’); (b) Two twin studies (1988-1991) in which Health and Lifestyle Questionnaires were sent to the members of Cohort 1 and an additional group born from 1964 to 1971 (‘Cohort 2’)^30^; with similar questionnaires also sent to immediate family members of the twins; (c) The Anxiety and Depression study (assessed twice, once by questionnaire and once by telephone interview)^33^ drawn from Cohort 1 and Cohort 2 but selected to include mainly individuals with extreme high or low neuroticism scores from the studies in (b) and members of their immediate families. The TCI data were obtained from two twin studies (1988-1991) from Cohort 1 and 2, and the MPQ data as part of the Gambling Study (cohort 2).[^29^](#_ENREF_29)

Altogether, the EPQ was administered four times, the NEO-FFI twice, and the TCI, NEO-PI-R and MPQ once. Data from one time point per inventory per participant was retained, with time points chosen to minimize time difference. We first selected EPQ item data at first assessment, because the items were available for the majority of the subjects. TCI data was obtained at the same time point and the MPQ assessment was close in time. When available, the NEO-PI-R was chosen over the shorter NEO-FFI. The genotypic data used in the current study are derived from multiple waves of genotyping. DNA samples were collected in accordance with standard protocols and submitted to different genotype centres using different Illumina SNP platforms (317 single, 370 single, 370 duo, 670 quad, 610 quad).[^25^](#_ENREF_25) Phenotypic and genotypic data collections were approved by the QIMR Human Research Ethics Committee and informed consent was obtained from all participants.

*23. SAGE-COGA34, 35 — United States of America.* The Collaborative Study on the Genetics of Alcoholism (COGA) is a multi-site study funded by the National Institute on Alcohol Abuse and Alcoholism (NIAAA) and National Institute on Drug Abuse (NIDA) that aims to characterize the familial transmission of alcoholism and related phenotypes and identify susceptibility genes. A total of 649 participants drawn from COGA completed the TCI, 647 of whom had corresponding genome-wide genotyping successfully performed as part of The Study of Addiction: Genetics and Environment (SAGE). SAGE is part of the Gene Environment Association Studies initiative funded by the National Human Genome Research Institute (NHGRI). The mean age of COGA participants with both SAGE genotypes and TCI phenotypes was 40.8 years (SD=10.8) and women constituted 45.6% of the total sample (M=40.9, SD=10.4, versus in men M=40.8, SD=11.1).

*24. SAGE-COGEND5, 6 — United States of America*. The Collaborative Genetic Study of Nicotine Dependence (COGEND) was initiated as a three-part program project grant funded through the National Cancer Institute (NCI; PI: Laura Bierut). The three projects included a study of the familial transmission of nicotine dependence, a genetic study of nicotine dependence, and a study of the relationship of nicotine dependence with nicotine metabolism. The primary goal is to detect, localize, and characterize genes that predispose or protect an individual with respect to heavy tobacco consumption, nicotine dependence, and related phenotypes and to integrate these findings with the family transmission and nicotine metabolism findings. As a part of this study, item-level NEO-FFI data were available from 2,712 participants, 1,279 of whom had corresponding genome-wide genotyping successfully performed as part of The Study of Addiction: Genetics and Environment (SAGE). SAGE is part of the Gene Environment Association Studies initiative funded by the National Human Genome Research Institute (NHGRI). The mean age of COGEND participants with both SAGE genotypes and NEO-FFI phenotypes was 36.5 years (SD=5.6) and women constituted 65.1% of the total sample (M=36.6, SD=5.6, versus in men M=36.4, SD=5.5).

*25. SardiNIA – Italy.* The SardiNIA study includes 6,148 related individuals from four towns in the Ogliastra province of Sardinia, Italy.^21^ These individuals represent 62% of the population in these towns. Valid personality data were available for 5,669 individuals, of which 3,972 were genotyped (56.7% women). The mean age of all participants was 42.8 years (SD=17). The mean age of the men was 43.0 years (SD=18), and of the women 42.4 years (SD=17). The sample has been described in more detail by Terracciano and co-authors.^31^

*26. SHIP – Germany.* The Study of Health in Pomerania (SHIP) is a cross-sectional survey in West Pomerania, the north-east area of Germany[^30^](#_ENREF_30)^,^ [^31^](#_ENREF_31). A sample from the population aged 20 to 79 years was drawn from population registries. First, the three cities of the region (with 17,076 to 65,977 inhabitants) and the 12 towns (with 1,516 to 3,044 inhabitants) were selected, and then 17 out of 97 smaller towns (with less than 1,500 inhabitants), were drawn at random. Second, from each of the selected communities, subjects were drawn at random, proportional to the population size of each community and stratified by age and gender. Only individuals with German citizenship and main residency in the study area were included. Finally, 7,008 subjects were sampled, with 292 persons of each gender in each of the twelve five-year age strata. In order to minimize drop-outs by migration or death, subjects were selected in two waves. The net sample (without migrated or deceased persons) comprised 6,267 eligible subjects. Selected persons received a maximum of three written invitations. In case of non-response, letters were followed by a phone call or by home visits if contact by phone was not possible. The SHIP population finally comprised 4,308 participants (corresponding to a final response of 68.8%). From 2007 to 2010, the “Life-Events and Gene-Environment Interaction in Depression” (LEGEND) study was conducted.[^31^](#_ENREF_31) Until the beginning of LEGEND, 639 participants from the baseline sample SHIP-0 were either deceased (n=383) or refused further participation (n=256). Thus, 3,669 participants were invited to take part in the LEGEND study. During the conduction of LEGEND 92 participants were deceased and 1,011 subjects refused participation in the LEGEND study. 132 subjects did not respond to repeated efforts of contact (at least three written invitations, 10 telephone calls and five home visits). 35 subjects agreed to participate but missed all appointments. Among the 2,400 subjects who participated in the LEGEND study, we excluded 134 subjects from the analyses because of unreliable information or inconsistencies in the interview according to the judgment of the interviewer and the supervisor. Full data sets (GWAS & extraversion) were available for 2,213 subjects.

*27. STR*[*^32^*](#_ENREF_32) *— Sweden.* For the Swedish Twin Registry (STR) a cohort of twins born in 1926–67 was compiled in 1970, by use of nationalized birth registrations. A register consisting all 50,000 twin births was established. Members of like-sexed pairs from the cohort born in 1926–58 were sent a questionnaire in 1972–73. Responses were received from 36,535 individuals including 14,000 twin pairs. The EPI was included to assess personality and completed by 30,276 individuals (52.3% female). Among these individuals, 22,390 subjects were invited to the TwinGene study conducted between 2004 and 2008. In total, 12,591 (56%) individuals participated by donating blood to the study, and by answering questionnaires about life style and health. DNA from 9,896 individual subjects was sent to Uppsala, Sweden for genome wide genotyping with Illumina OmniExpress bead chip (all available dizygous twins + one twin from each available MZ twin pair). Genotyping results for 9,836 subjects and 731,442 autosomal SNPs passed the initial lab-based quality control (QC). In further QC, SNPs with more than 3% missing information (GENO > 0.03) (n=3,922), a minor allele frequency below 1% (n=79,893), or a Hardy-Weinberg equilibrium (HWE) test p-value <= 1e-07 (n=3,071), were excluded. Individuals with low genotyping success (MIND > 0.03) (n=10), heterozygosity of X-chromosomes in males (n=36), deviations in heterozygosity of more than 5 standard deviations (SD) from the population mean (n=49), or detection of unknown (cryptic) relatedness (n=124), were excluded. After the QC there were 9,617 individuals and 644,556 autosomal SNPs remaining.

*28. CROATIA-Vis*[*^33^*](#_ENREF_33) *— Croatia.* Adult participants living in the villages of Komiža and Vis on the Croatian island of Vis were recruited in May 2003 and May 2004 for a large genetic study. Croatia has 15 Adriatic Sea islands with populations greater than 1,000. The villages on the islands have unique population histories and have preserved their isolation from other villages and the outside world through many centuries. Informed consents, procedures and questionnaires were reviewed and approved by relevant ethics committees in Scotland and Croatia. All individuals over 18 years old and resident on the Island of Vis were invited to participate in this study. More information about this sample can be found in [^33^](#_ENREF_33). As a part of the interview participants also completed the Eysenck Personality Questionnaire-Revised (short-form; EPQ-R). Seventy percent of the villages’ adult population took part in the study, a total of 918 individuals (531 female; 57.8%), 9 of whom have all missing data. The mean age was 56.4 years (SD=15.5; female M=56.7, SD=16, male M=55.9, SD=14.9). The data were collected between 2003 and 2004.

*29. YOUNG FINNS*[*^34^*](#_ENREF_34) *— Finland.* The Young Finns study (YFS) is an ongoing, population-based prospective cohort study started in 1980 with a baseline sample of 3,596 children and adolescents derived from six birth cohorts aged 3, 6, 9, 12, 15, and 18 years at baseline (<http://youngfinnsstudy.utu.fi/>). The participants were recruited from five areas according to locations of university cities with a medical school (Helsinki, Kuopio, Oulu, Tampere and Turku). In each location, individuals from urban and surrounding rural areas were selected at random from the population registry (based on their unique personal social security number from) to be invited in the study. Subsequent data collection waves have been carried out in 1983, 1986, 1989, 1992, 1997, 2001, 2007 and 2012. Personality data for the NEO-FFI were collected with mailed questionnaires in 2007 from 2,058 participants of whom 2,057 were included in the study with NEO-FFI data (one participant had all missing data). The mean age of participants was 37.6 years (SD=5) for women and men at the time of personality assessment, and 1,212 participants (58.9%) were female.

*30. GENERATION SCOTLAND: SCOTTISH FAMILY HEALTH STUDY*[*^35^*](#_ENREF_35) *– Scotland (United Kingdom)*. The Generation Scotland: Scottish Family Health Study (GS:SFHS) is a collaboration between the Scottish Universities and the NHS, funded by the Chief Scientist Office of the Scottish Government. GS:SFHS is a family-based genetic epidemiology cohort with DNA, other biological samples (serum, urine and cryopreserved whole blood) and socio-demographic and clinical data from ~24,000 volunteers, aged 18-98 years, in ~7,000 family groups. Participants were recruited across Scotland, with some family members from further afield, from 2006 - 2011. Most (87%) participants were born in Scotland and 96% in the UK or Ireland. GS:SFHS operates under appropriate ethical approvals, and all participants gave written informed consent. From this cohort, 9,860 individuals were successfully genotyped using the Illumina OmniExpress + Exome genotyping array. 1000 genomes SNP dosages were imputed using SHAPEIT_v2 and IMPUTE2 in conjunction with the Phase I integrated variant set release (v3) all ethnicities reference panel (MAR 2012). Extraversion was measured by the Eysenck Personality Questionnaire Revised Short Form, a self-report questionnaire requiring a yes/no response on 24 items. Extraversion questionnaire data were obtained for 9,783 individuals who also had 1000G imputed genotype data available. Summed questionnaire scores were used for association analysis of the selected replication SNPs using ProbABEL from the GenABEL suite of programs (www.genabel.org).[^36^](#_ENREF_36)

**Personality assessment**

Item data from the following personality inventories were included: the Neuroticism Extraversion Openness to Experience personality inventory revised (NEO-PI-R; 240 items, 48 items for Extraversion), the abbreviated NEO Five Factor Inventory (NEO-FFI; 60 items, 12 Extraversion items), the 30-item short version of the NEO-Five-Factor Model (NEO-FFI-30), the Eysenck Personality Questionnaire Revised (EPQ-R; 48 items, 12 items for Extraversion), the Junior EPQ (JEPQ; 81 items, 24 Extraversion items), the Eysenck Personality Inventory (EPI; 20 items, 9 for Extraversion), the EPI-based Amsterdamse Biografische Vragenlijst (ABV; 21 Extraversion items), the Big Five item set of the International Personality Item Pool (IPIP; 50 items, 10 items for Extraversion), the Temperament and Character Inventory version 9 (TCI; 240 items, of which 7-13 items were selected for Extraversion) and the Multidimensional Personality Questionnaire (MPQ; 198 items, of which 72 items were selected for Extraversion). The number of items included per cohort varied slightly; for the complete overview, see Van den Berg et al. (2014)[^37^](#_ENREF_37).

**Supplementary Table 1.** Genotyping and imputation in the 29 discovery cohorts and 1 replication cohort of the Genetics of Personality Consortium

|  | **Cohort** | **Genotyping platforms(s)** | **HWE *P*-value *** | **SNP call rate *** | **Sample call rate *** | **Imputation reference set** | **Imputation software** | **Association software** |
| --- | --- | --- | --- | --- | --- | --- | --- | --- |
| 1 | ALSPAC | Illumina 550K | >5 x 10-7 | >0.95 | >0.97 | 1000G | MACH | MACH2QTL |
| 2 | BLSA | Illumina 550K | ≥1 x 10-5 | ≥0.99 | ≥0.985 | 1000G | MACH | Merlin-offline |
| 3 | BRESCIA | Affymetrix 6.0 | >1 x 10-6 | >0.95 | >0.96 | 1000G | IMPUTE | SVS 7 GoldenHelix |
| 4 | CHICAGO | Affymetrix 6.0 | >1 x 10-4 | >0.95 | >0.90 | 1000G | IMPUTE | SNPTEST |
| 5 | CILENTO | Illumina 370 K Illumina OmniExpress 700K | NA | >0.95 | >0.90 | 1000G | MACH /minimac | R, GenABEL /ProbABEL |
| 6 | EGCUT | Illumina Human370CNV & OmniExpress770k | >1 x 10-6 | >0.95 | >.095 | 1000G | IMPUTE | SNPTEST |
| 7 | ERF | Illumina 318K, 370K, 610K | >1 x 10-6 | ≥0.95 | ≥0.95 | 1000G | minimac | ProbABEL |
| 8 | FTC EPI | Illumina 670K | >1 x 10-6 | >0.95 | > 0.95 | 1000G | IMPUTE | SNPTEST |
| 9 | FTC NEO | Illumina 670K | >1 x 10-6 | >0.95 | >0.95 | 1000G | IMPUTE | SNPTEST |
| 10 | HBCS | Modified Illumina 610 k | >1 x 10-6 | >0.95 | >0.95 | 1000G | MACH | Plink, ProbABEL |
| 11 | CROATIA-Korcula | Illumina HumanHap 370 CNV Duochip | >1 x 10-6 | >0.98 | >0.97 | 1000G | ShapeIT / IMPUTE | ProbABEL |
| 12 | LBC1921 | Illumina610-Quadv1 | >1 x 10-3 | ≥0.98 | ≥0.95 | 1000G | Minimac | MACH2QTL |
| 13 | LBC1936 | Illumina610-Quadv1 | >1 x 10-3 | ≥0.98 | ≥0.95 | 1000G | Minimac | MACH2QTL |
| 14 | MCTFR | Illumina Human660W-Quad Array | >1 x 10-7 | >0.99 | removed samples with: non-calls in more than 5000 markers; GenCall_10 < .75; GenCall_50 < .9009 | 1000G | Beagle / Minimac | RFGLS package for R |
| 15 | MGS | Affymetrix 6.0 | >1 x 10-6 | >0.98 | No exclusions; all were > 0.99 | 1000G | SHAPEIT / IMPUTE | ProbABEL |
| 16 | NBS | Illumina 317K | >1 x 10-6 | >0.95 | 0.9 | 1000G | MACH/minimac | mach2qtl |
| 17 | NESDA | Affymetrics 5.0-Perlegen  Affymetrix 6.0  Affymetrix 6 907k | >1 x 10-6 | >0.95 | >0.9 | 1000G | IMPUTE | PLINK |
| 18 | NTR | Affymetrics 5.0-Perlegen  Illumina 370  Illumina 660 Affymetrix 6.0  Affymetrix 6 907k  Illumina Omni Express 1M | >1 x 10-6 | 0.95 | 0.9 | 1000G | IMPUTE | PLINK |
| 19 | ORCADES | Illumina HAP300 854 subjs Illumina OmniX 1067 Subjs Illumina Omni1 301 Subjs | >1 x 10-6 | >0.97 | >0.98 | 1000G | ShapeIT / IMPUTE | R, GenABEL/ProbABEL |
| 20 | PAGES | Illumina Human Hap 300 | >1 x 10-6 | >0.99 | >0.96 | 1000G | MACH / minimac | SNPTEST |
| 21 | QIMR adolescents | Illumina 610 quad | >1 x 10-6 | >0.95 | >0.95 | 1000G (less SNPs with <2 copies of minor allele in reference populations) | MACH / minimac | Merlin-offline |
| 22 | QIMR adults | Illumina 610 quad  Illumina 670 quad  Illumina 370 duo Illumina 370 single Illumina 317 single | >1 x 10-6 | >0.95 | >0.95 | 1000G (less SNPs with <2 copies of minor allele in reference populations) | MACH / minimac | Merlin-offline |
| 23 | SAGE-COGA | Illumina 1M | >1 x 10-6 | >0.95 | >0.95 | 1000G | IMPUTE | SNPTEST |
| 24 | SAGE COGEND | Illumina 1M | >1 x 10-6 | >0.95 | >0.95 | 1000G | IMPUTE | SNPTEST |
| 25 | SardiNIA | Affymetrix 10k, 500k, 1M | ≥1 x 10-7 | ≥0.95 | ≥0.95 | 1000G | MACH | EPACTS Q.EMMAX |
| 26 | SHIP | Affymetrix 6.0 | >1 x 10-4 | >0.8 | >0.92 | 1000G | IMPUTE | SNPTEST |
| 27 | STR | Illumina OmniExpress | >1 x 10-6 | >0.97 | >0.97 | 1000G | IMPUTE | PLINK |
| 28 | CROATIA-Vis | Illlumina Infinium HumanHap 300 Bead chip | >1 x 10-6 | >0.98 | >0.97 | 1000G | ShapeIT / IMPUTE | ProbABEL |
| 29 | YFS | Custom Illumina Human 670k BeadChip | >1 x 10-6 | ≥0.95 | ≥0.95 | 1000G | IMPUTE | SNPTEST |
| 30 | GS:SFHS | Illumina OmniExpress + Exome genotyping array | NA | NA | NA | 1000G | ShapeIT / IMPUTE | ProbABEL |

* Quality control of genotyped SNPs prior to imputation (Inclusion criteria)

**Supplementary Table 2.** Genomic inflation factors **(**Lambda’s) for Extraversion results in each discovery cohort participating in the Genetics of Personality Consortium

| **Cohort** | **Lambda** | **Cohort** | **Lambda** |
| --- | --- | --- | --- |
| ALSPAC | 0.96 | NBS | 0.97 |
| BLSA | 0.98 | NESDA | 0.97 |
| BRESCIA | 0.98 | NTR | 0.99 |
| CHICAGO | 0.98 | ORCADES | 0.97 |
| CILENTO | 0.96 | PAGES | 0.96 |
| EGCUT | 0.99 | QIMR adolescents | 0.98 |
| ERF | 0.97 | QIMR adults | 0.97 |
| FTC EPI | 0.96 | SAGE-COGA | 0.90 |
| FTC NEO | 0.98 | SAGE-COGEND | 0.90 |
| HBCS | 0.96 | SardiNIA | 0.99 |
| CROATIA-Korcula | 0.97 | SHIP | 0.98 |
| LBC1921 | 0.98 | STR | 0.99 |
| LBC1936 | 0.98 | CROATIA-Vis | 0.97 |
| MCTFR | 0.99 | YFS | 0.96 |
| MGS | 0.96 | ***Meta-analysis*** | 1.02 |

**Supplementary Table 3.** Top 74 SNPs from meta-analysis of GWA results for Extraversion in the Genetics of Personality Consortium (*P* < 1*10^-5^)

|  |  |  |  |  | **Results meta-analysis discovery cohorts** | | | | **Results replication cohort** | | |
| --- | --- | --- | --- | --- | --- | --- | --- | --- | --- | --- | --- |
| **SNP** | **Chr_bp** | **A1** | **A2** | **MAF** | **Number of cohorts** | **Effect** | **SE** | **P-value** | **Effect** | **SE** | **P-value** |
| rs2024488 | 2_217662968 | a | g | 0.30153 | 28 | -0.0303 | 0.0059 | 2.939E-07 | .0285 | .0164342 | .082442 |
| rs2712162 | 2_217661788 | t | c | 0.30246 | 28 | -0.0300 | 0.0059 | 3.872E-07 | .0278 | .0163777 | .089472 |
| rs797182 | 12_10900487 | a | g | 0.36128 | 28 | -0.0277 | 0.0056 | 6.673E-07 | -.0135 | .0152612 | .377213 |
| rs8010306 | 14_37150160 | a | g | 0.05989 | 16 | 0.0629 | 0.0128 | 8.730E-07 | .0180 | .0313783 | .566502 |
| rs117292860 | 19_2227621 | a | c | 0.17926 | 20 | 0.0553 | 0.0113 | 9.191E-07 | -.0350 | .0239138 | .143687 |
| rs79870297 | 4_186614953 | t | c | 0.23932 | 28 | -0.0323 | 0.0066 | 9.921E-07 | -.0203 | .0173826 | .243252 |
| rs1088236 | 12_10899740 | t | g | 0.35622 | 28 | -0.0270 | 0.0056 | 0.000001235 | -.0146 | .0152161 | .338657 |
| rs79953023 | 4_186603023 | t | g | 0.25198 | 28 | 0.0315 | 0.0065 | 0.000001517 | .0091 | .0171432 | .594888 |
| rs811615 | 12_10900541 | t | c | 0.35554 | 28 | -0.0268 | 0.0056 | 0.000001591 | -.0147 | .0152693 | .334701 |
| rs797183 | 12_10900554 | a | c | 0.35562 | 28 | 0.0268 | 0.0056 | 0.000001595 | .0147 | .0152676 | .336788 |
| rs8012165 | 14_37150410 | a | t | 0.05947 | 16 | -0.0597 | 0.0125 | 0.000001907 | -.0235 | .0316400 | .456751 |
| rs11053926 | 12_10901028 | t | c | 0.35173 | 28 | 0.0265 | 0.0056 | 0.000002094 | .0148 | .0153021 | .334706 |
| rs797184 | 12_10900925 | a | g | 0.35076 | 28 | 0.0264 | 0.0056 | 0.000002300 | .0153 | .0152901 | .318429 |
| rs79427853 | 4_186611819 | a | g | 0.25172 | 28 | 0.0302 | 0.0064 | 0.000002369 | .0130 | .0169092 | .441163 |
| rs2415358 | 14_37151869 | a | g | 0.05871 | 16 | 0.0591 | 0.0125 | 0.000002402 | .0207 | .0316925 | .512795 |
| rs4513000 | 14_37151542 | t | c | 0.05837 | 16 | -0.0590 | 0.0125 | 0.000002573 | -.0215 | .0316673 | .497696 |
| rs28713249 | 14_37151057 | t | g | 0.05837 | 16 | 0.0589 | 0.0125 | 0.000002616 | .0224 | .0316611 | .479992 |
| rs932570 | 20_61028986 | t | c | 0.45229 | 28 | -0.0282 | 0.0060 | 0.000002711 | -.0262 | .0158993 | .098807 |
| rs4477602 | 14_37151655 | a | g | 0.05839 | 15 | -0.0598 | 0.0128 | 0.000002778 | -.0207 | .0317595 | .515553 |
| rs10136792 | 14_37151212 | a | g | 0.05833 | 16 | -0.0588 | 0.0125 | 0.000002780 | -.0221 | .0316638 | .485261 |
| rs4474609 | 14_37151117 | a | g | 0.05833 | 16 | -0.0587 | 0.0125 | 0.000002836 | -.0223 | .0316622 | .482056 |
| rs12109897 | 5_172628038 | a | g | 0.07341 | 21 | -0.0490 | 0.0105 | 0.000002965 | .0141 | .0257128 | .583425 |
| rs797177 | 12_10894916 | a | g | 0.34982 | 28 | 0.0258 | 0.0055 | 0.000003297 | .0138 | .0150477 | .360741 |
| rs797176 | 12_10894685 | a | t | 0.34964 | 28 | 0.0256 | 0.0055 | 0.000003828 | .0138 | .0150350 | .359432 |
| rs7002652 | 8_93282549 | a | c | 0.46037 | 27 | -0.0250 | 0.0054 | 0.000004093 | -.0256 | .0148972 | .085817 |
| rs13282281 | 8_93297037 | a | c | 0.42965 | 27 | -0.0251 | 0.0055 | 0.000004124 | -.0255 | .0149594 | .088092 |
| rs7300727 | 12_10902579 | a | g | 0.32812 | 28 | 0.0262 | 0.0057 | 0.000004149 | .0195 | .0157664 | .216274 |
| rs140417730 | 15_100587525 | a | g | 0.07567 | 7 | -0.1226 | 0.0266 | 0.000004173 | .0186 | .0420539 | .658813 |
| rs2339583 | 8_93274702 | c | g | 0.44775 | 27 | -0.0250 | 0.0054 | 0.000004254 | -.0234 | .0148983 | .117016 |
| rs13410278 | 2_168641637 | a | g | 0.04252 | 10 | -0.0685 | 0.0149 | 0.000004382 | -.0266 | .0378979 | .482464 |
| rs13280948 | 8_93269692 | a | g | 0.44223 | 27 | -0.0250 | 0.0054 | 0.000004402 | -.0238 | .0149265 | .111291 |
| rs1030064 | 2_217665966 | t | g | 0.2976 | 28 | -0.0273 | 0.0060 | 0.000004424 | .0284 | .0164976 | .085092 |
| rs6442336 | 3_12870886 | t | c | 0.24878 | 29 | 0.0304 | 0.0066 | 0.000004841 | .0178 | .0177543 | .316834 |
| rs797179 | 12_10895357 | a | g | 0.19118 | 28 | 0.0321 | 0.0070 | 0.000005113 | -.0041 | .0183290 | .823724 |
| rs12681205 | 8_93298074 | a | c | 0.42747 | 27 | -0.0249 | 0.0055 | 0.000005453 | -.0255 | .0149543 | .088361 |
| rs3732679 | 3_12867485 | a | c | 0.32128 | 29 | 0.0278 | 0.0061 | 0.000005530 | .0154 | .0162908 | .344030 |
| rs7144276 | 14_37144607 | a | t | 0.01194 | 19 | 0.0527 | 0.0116 | 0.000005632 | .0262 | .0320563 | .413166 |
| rs76669452 | 8_49756520 | a | g | 0.07563 | 20 | -0.0451 | 0.0099 | 0.000005645 | -.0490 | .0255501 | .054907 |
| rs75245863 | 2_1960391 | t | c | 0.12107 | 25 | 0.0400 | 0.0088 | 0.000005714 | .0043 | .0233747 | .854378 |
| rs56095028 | 13_56060991 | a | g | 0.06138 | 24 | 0.0485 | 0.0107 | 0.000006418 | -.0060 | .0237105 | .799023 |
| rs3732680 | 3_12873265 | a | g | 0.23563 | 29 | -0.0296 | 0.0066 | 0.000006539 | -.0213 | .0178185 | .232522 |
| rs42385 | 16_6536933 | a | c | 0.31747 | 28 | -0.0266 | 0.0059 | 0.000006916 | .0177 | .0157367 | .259704 |
| rs10891937 | 11_116126804 | a | g | 0.40532 | 29 | -0.0246 | 0.0055 | 0.000006940 | -.0077 | .0150088 | .608712 |
| rs12668816 | 7_996398 | t | c | 0.24545 | 20 | -0.0484 | 0.0108 | 0.000007000 | .0122 | .0202365 | .547203 |
| rs1444504 | 8_93279552 | t | c | 0.46102 | 27 | -0.0244 | 0.0054 | 0.000007039 | -.0247 | .0148723 | .096628 |
| rs78961361 | 19_53267419 | t | g | 0.06948 | 12 | -0.0906 | 0.0202 | 0.000007198 | -.0043 | .0309257 | .888678 |
| rs9940817 | 16_6535942 | t | c | 0.31698 | 28 | 0.0266 | 0.0059 | 0.000007215 | -.0186 | .0157347 | .236609 |
| rs35682092 | 16_6536593 | a | g | 0.31724 | 28 | 0.0266 | 0.0059 | 0.000007325 | -.0180 | .0157413 | .252854 |
| rs3095263 | 16_6536110 | a | g | 0.31706 | 28 | -0.0266 | 0.0059 | 0.000007361 | .0186 | .0157377 | .238096 |
| rs118072569 | 10_13746323 | a | c | 0.0306 | 5 | 0.1149 | 0.0256 | 0.000007418 | .1249 | .0446190 | .005124 |
| rs1030065 | 2_217665415 | a | g | 0.29752 | 28 | -0.0267 | 0.0060 | 0.000007438 | .0280 | .0165086 | .081040 |
| rs3849571 | 3_81777716 | a | c | 0.35137 | 28 | -0.0253 | 0.0057 | 0.000007918 | -.0238 | .0155870 | .127118 |
| rs4392874 | 8_93271095 | a | g | 0.44681 | 27 | 0.0243 | 0.0054 | 0.000007925 | .0238 | .0148728 | .109583 |
| rs4786873 | 16_6539512 | t | g | 0.3085 | 28 | -0.0265 | 0.0059 | 0.000008048 | .0061 | .0159255 | .702294 |
| rs7620240 | 3_81762787 | t | g | 0.33795 | 28 | 0.0250 | 0.0056 | 0.000008248 | .0234 | .0155269 | .131664 |
| rs4684887 | 3_12865617 | t | c | 0.34277 | 29 | -0.0272 | 0.0061 | 0.000008441 | -.0179 | .0161063 | .266651 |
| rs13071278 | 3_82020103 | t | g | 0.32278 | 28 | -0.0260 | 0.0058 | 0.000008441 | -.0268 | .0159709 | .093089 |
| rs4684115 | 3_12865329 | a | g | 0.33409 | 29 | -0.0272 | 0.0061 | 0.000008479 | -.0177 | .0161028 | .272795 |
| rs668013 | 18_77407516 | a | g | 0.47749 | 19 | -0.0365 | 0.0082 | 0.000008684 | .0070 | .0161784 | .665188 |
| rs2029785 | 1_79960085 | a | g | 0.23686 | 27 | -0.0286 | 0.0064 | 0.000008707 | -.0499 | .0178715 | .005277 |
| rs9869182 | 3_12893668 | t | c | 0.27393 | 29 | -0.0292 | 0.0066 | 0.000008805 | -.0294 | .0177514 | .097773 |
| rs3772896 | 3_81751968 | t | c | 0.33958 | 28 | 0.0249 | 0.0056 | 0.000008927 | .0230 | .0155311 | .138186 |
| rs917647 | 16_6538699 | t | c | 0.31746 | 28 | -0.0261 | 0.0059 | 0.000009042 | .0015 | .0158747 | .924204 |
| rs6442333 | 3_12864386 | t | c | 0.30788 | 29 | -0.0269 | 0.0061 | 0.000009107 | -.0116 | .0160892 | .472272 |
| rs17790751 | 13_51875153 | a | g | 0.05135 | 18 | -0.0594 | 0.0134 | 0.000009113 | .0113 | .0314520 | .718641 |
| rs3894895 | 3_12888976 | t | c | 0.23674 | 29 | -0.0292 | 0.0066 | 0.000009138 | -.0218 | .0180256 | .227330 |
| rs4684886 | 3_12865282 | c | g | 0.31607 | 29 | 0.0270 | 0.0061 | 0.000009154 | .0137 | .0161144 | .396798 |
| rs4429609 | 3_12862561 | t | c | 0.28902 | 29 | -0.0277 | 0.0063 | 0.000009499 | -.0120 | .0159632 | .453836 |
| rs7621968 | 3_81951460 | a | g | 0.36606 | 28 | -0.0250 | 0.0056 | 0.000009523 | -.0138 | .0153777 | .370517 |
| rs114675436 | 1_80088978 | a | g | 0.19177 | 27 | -0.0355 | 0.008 | 0.000009533 | -.0549 | .0216094 | .011088 |
| rs3821548 | 3_81758728 | c | g | 0.33971 | 28 | 0.0248 | 0.0056 | 0.000009629 | .0232 | .0155295 | .135226 |
| rs11783791 | 8_49724699 | t | c | 0.09083 | 20 | -0.0423 | 0.0096 | 0.000009932 | -.0304 | .0238159 | .201441 |
| rs6789964 | 3_12864949 | t | c | 0.32412 | 29 | -0.0269 | 0.0061 | 0.000009950 | -.0152 | .0160204 | .341443 |
| rs13340130 | 3_81790970 | a | t | 0.34957 | 28 | -0.0254 | 0.0058 | 0.000009985 | -.0259 | .0156925 | .098580 |

**Supplementary Table 4.** Top 30 genes from meta-analysis of GWA results for Extraversion in the Genetics of Personality Consortium (*P* < 1*10^-5^)

| Gene | Nominal p-value | Chromosome |
| --- | --- | --- |
| *LOC101928162* | 0.00000287 | 12 |
| *LOC729506* | 0.00000893 | 5 |
| *PLEKHJ1* | 0.0000132 | 19 |
| *POU2F3* | 0.0000179 | 11 |
| *CRTAC1* | 0.0000297 | 10 |
| *RELN* | 0.0000569 | 7 |
| *PAX9* | 0.0000715 | 14 |
| *ADAM12* | 0.0000765 | 10 |
| *CYP2W1* | 0.0000916 | 7 |
| *BDNF-AS* | 0.0001 | 11 |
| *DNAH9* | 0.00012 | 17 |
| *PDE3A* | 0.00012 | 12 |
| *SORBS2* | 0.00012 | 4 |
| *LINC00434* | 0.00014 | 13 |
| *NDUFA4* | 0.00014 | 7 |
| *SNORA7A* | 0.00015 | 3 |
| *CAND2* | 0.00016 | 3 |
| *MIR6814* | 0.00018 | 21 |
| *NCMAP* | 0.00019 | 1 |
| *ADAM29* | 0.00019 | 4 |
| *IL34* | 0.0002 | 16 |
| *CHTF8* | 0.00021 | 16 |
| *NKX6-1* | 0.00022 | 4 |
| *LOC100131655* | 0.00023 | 18 |
| *PAPD4* | 0.00023 | 5 |
| *MIR4321* | 0.00024 | 19 |
| *TP63* | 0.00029 | 3 |
| *BDNF* | 0.0003 | 11 |
| *COX19* | 0.0003 | 7 |
| *MTMR9* | 0.00031 | 8 |

**Supplementary Figure 1.** Manhattan plots for Extraversion results in each cohort participating in the Genetic of Personality Consortium

ALSPAC


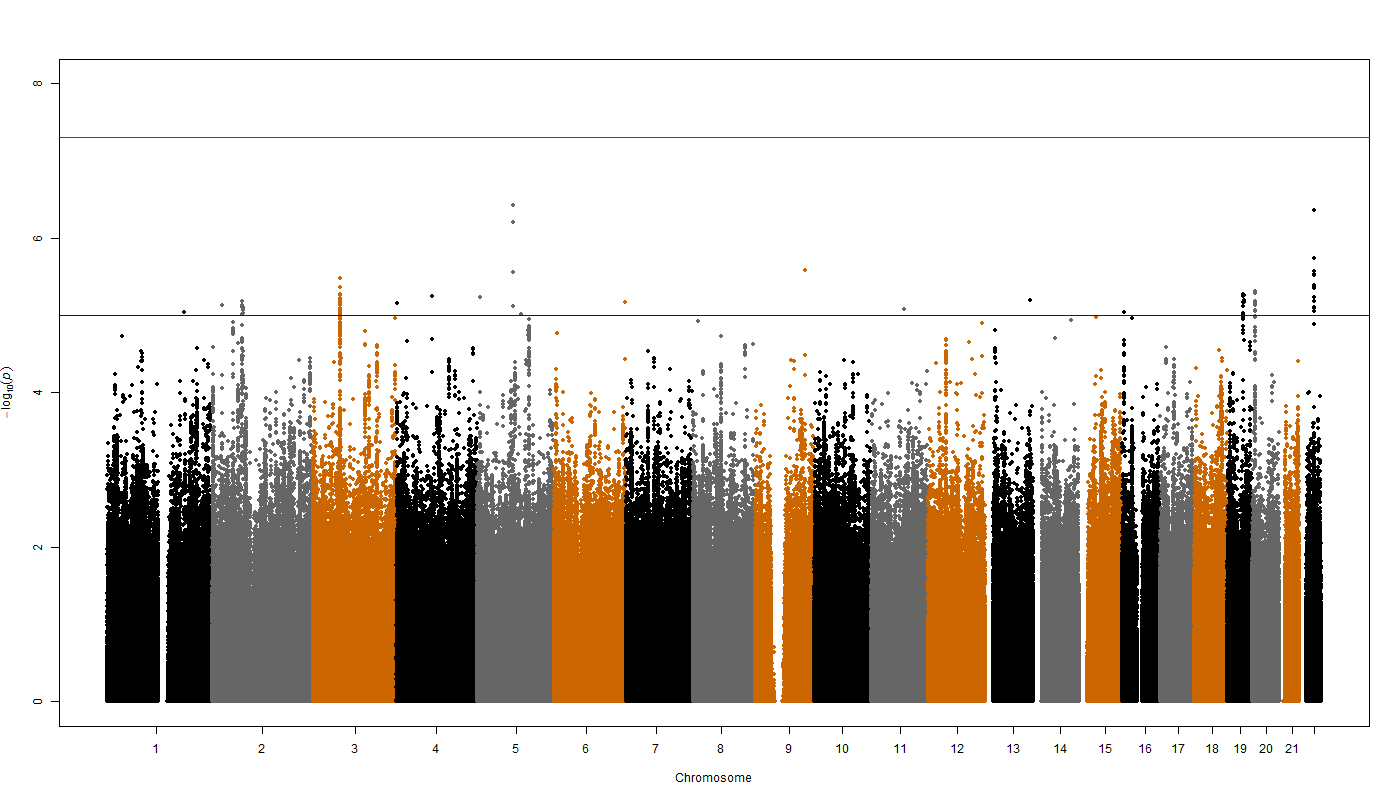


BLSA


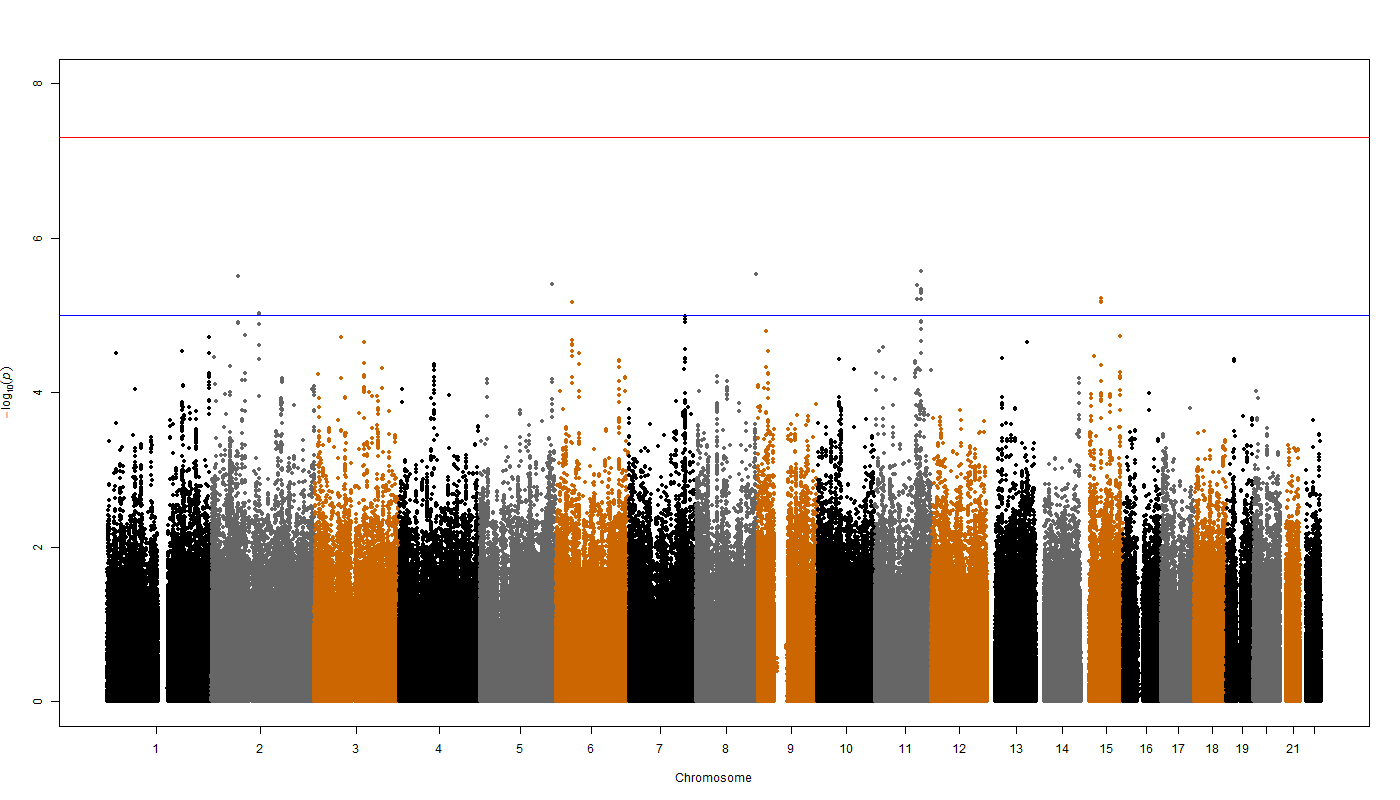


BRESCIA


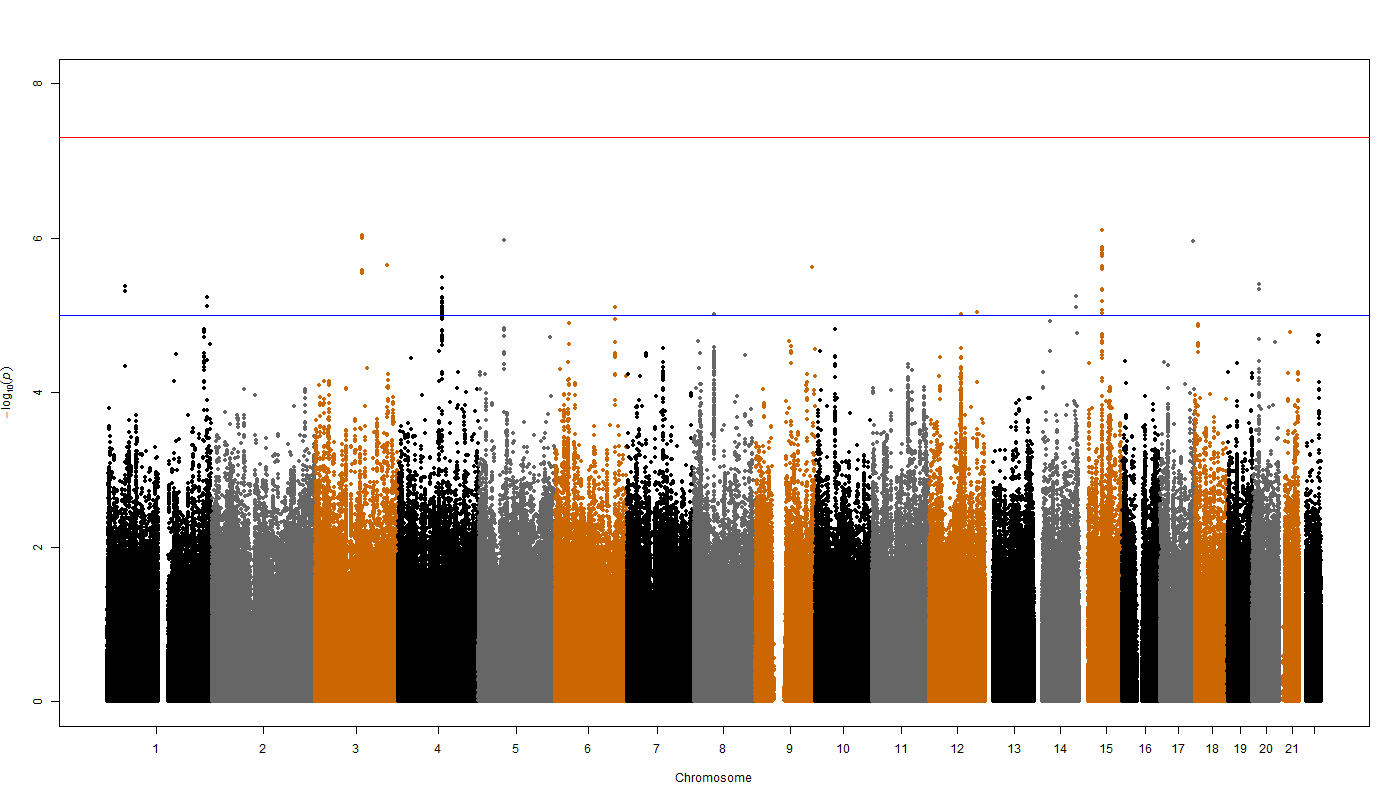


CHICAGO


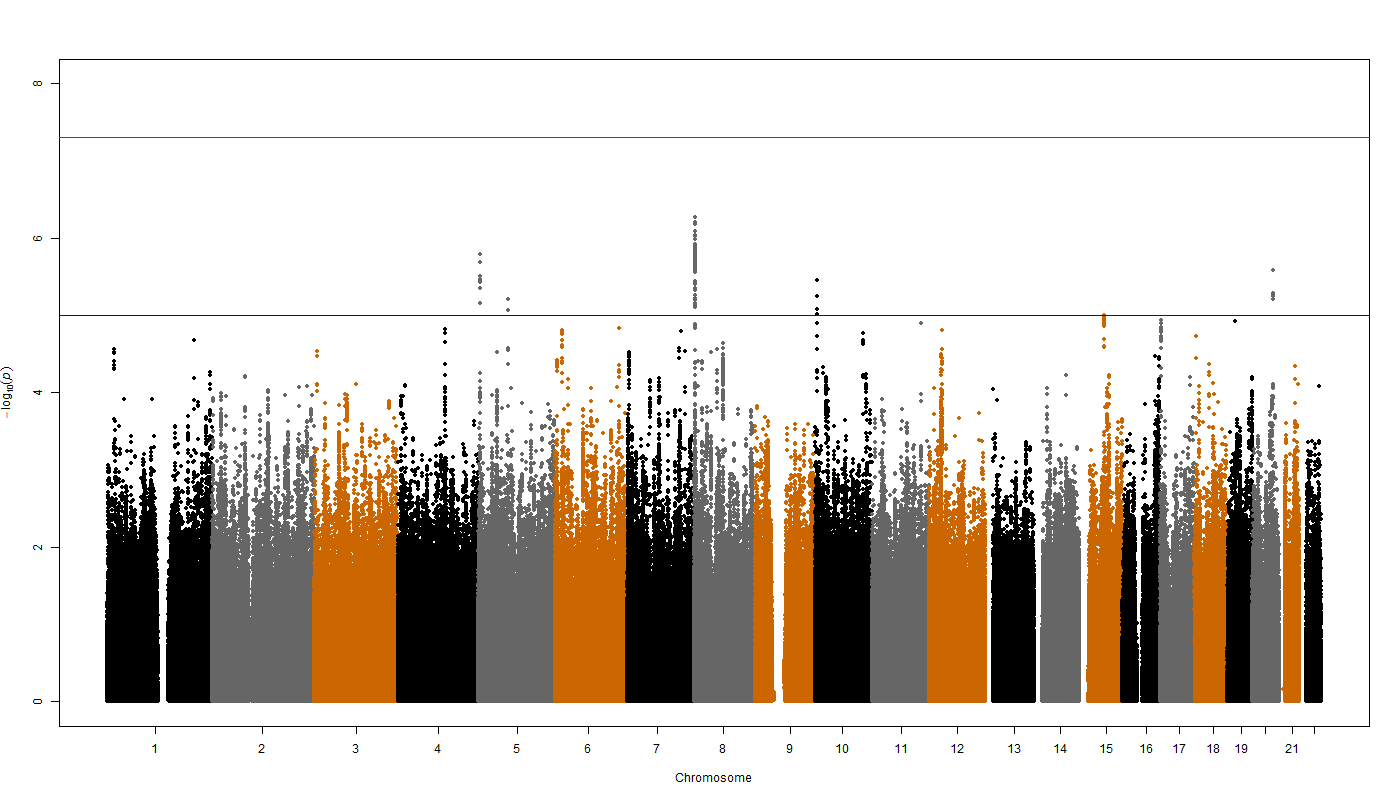


CILENTO


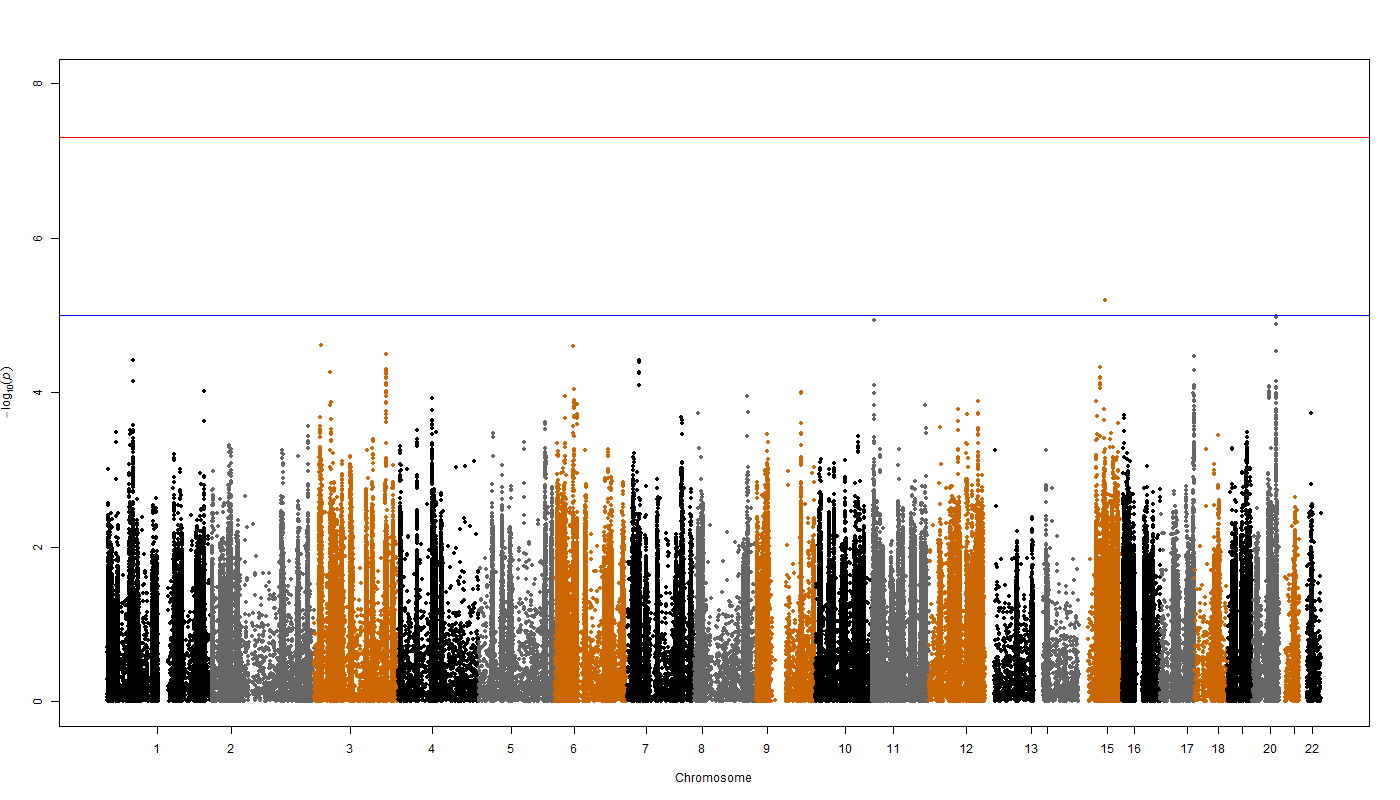


EGCUT


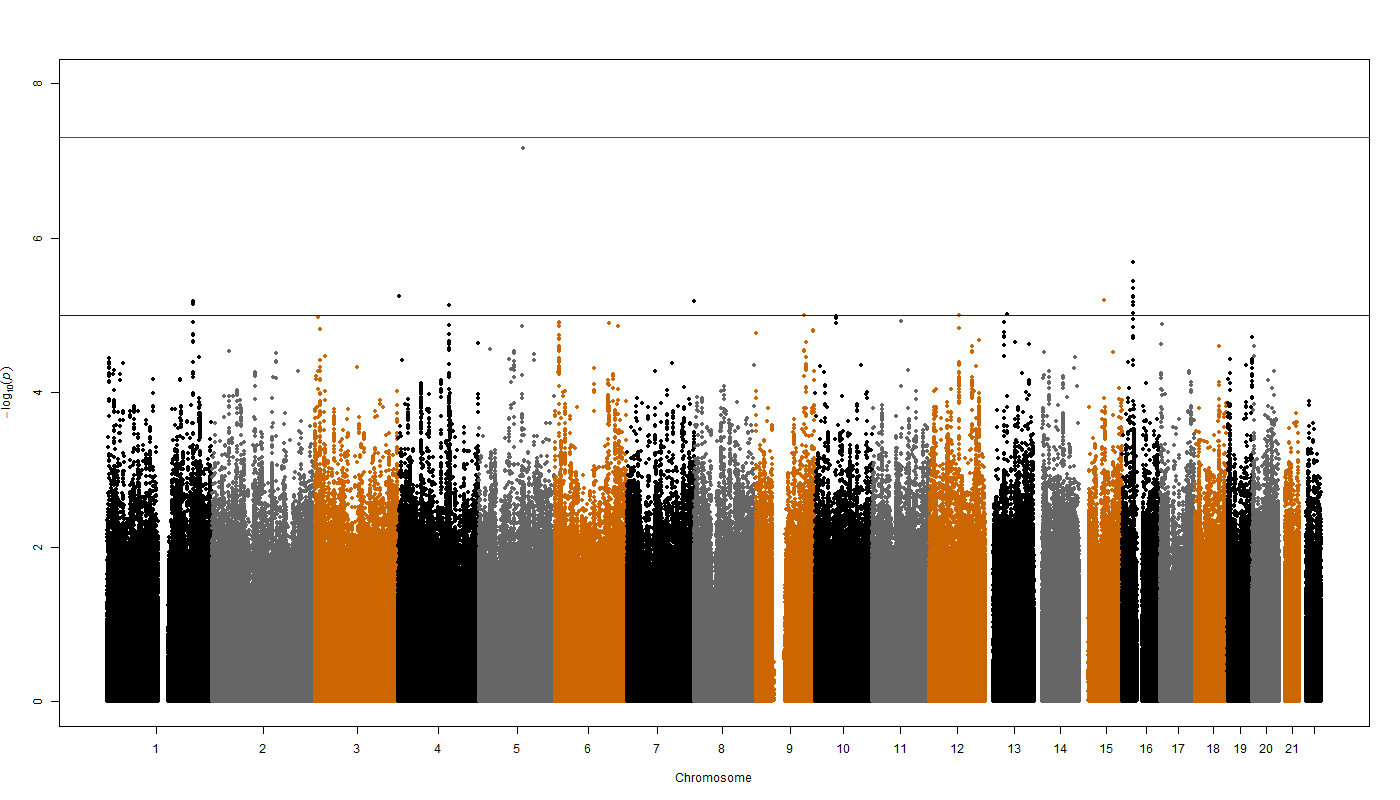


ERF


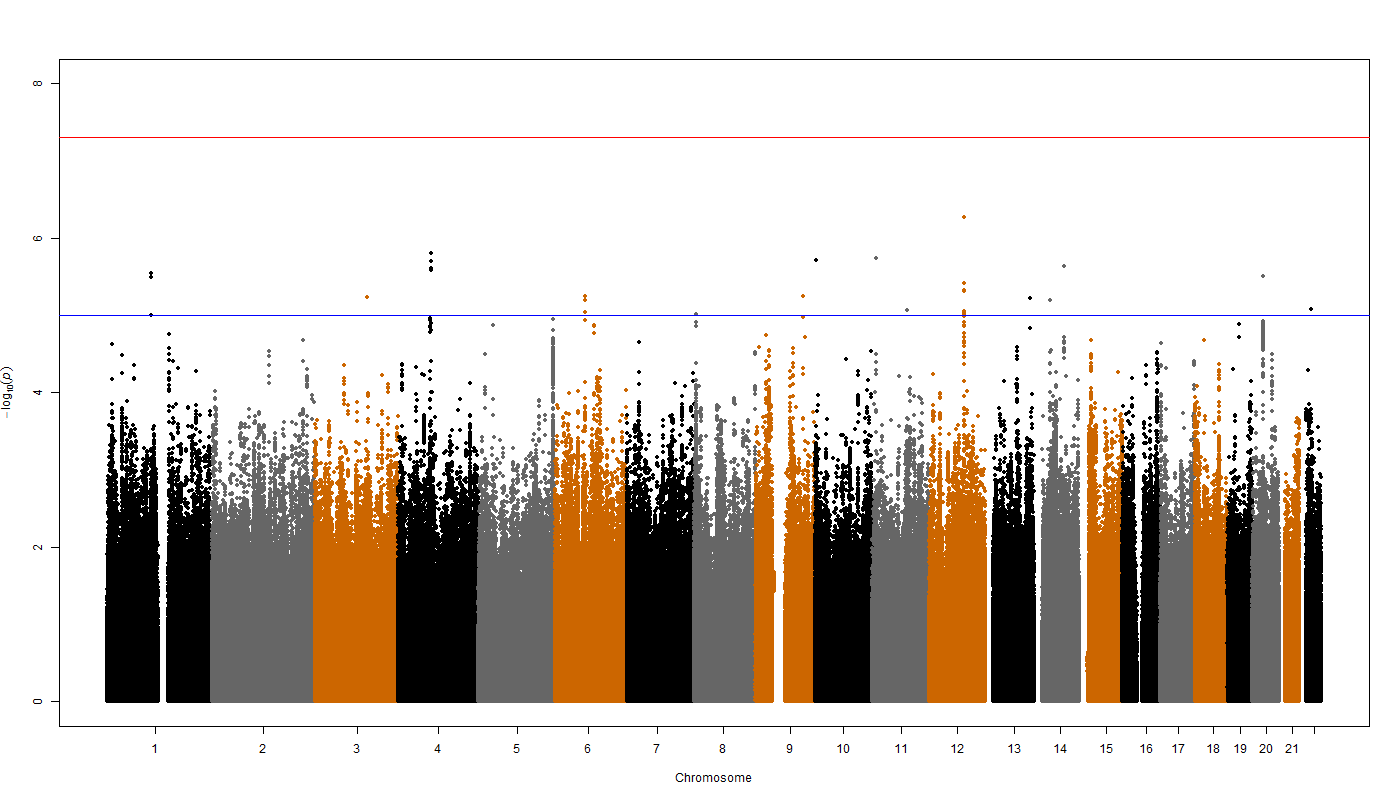


FTC EPI


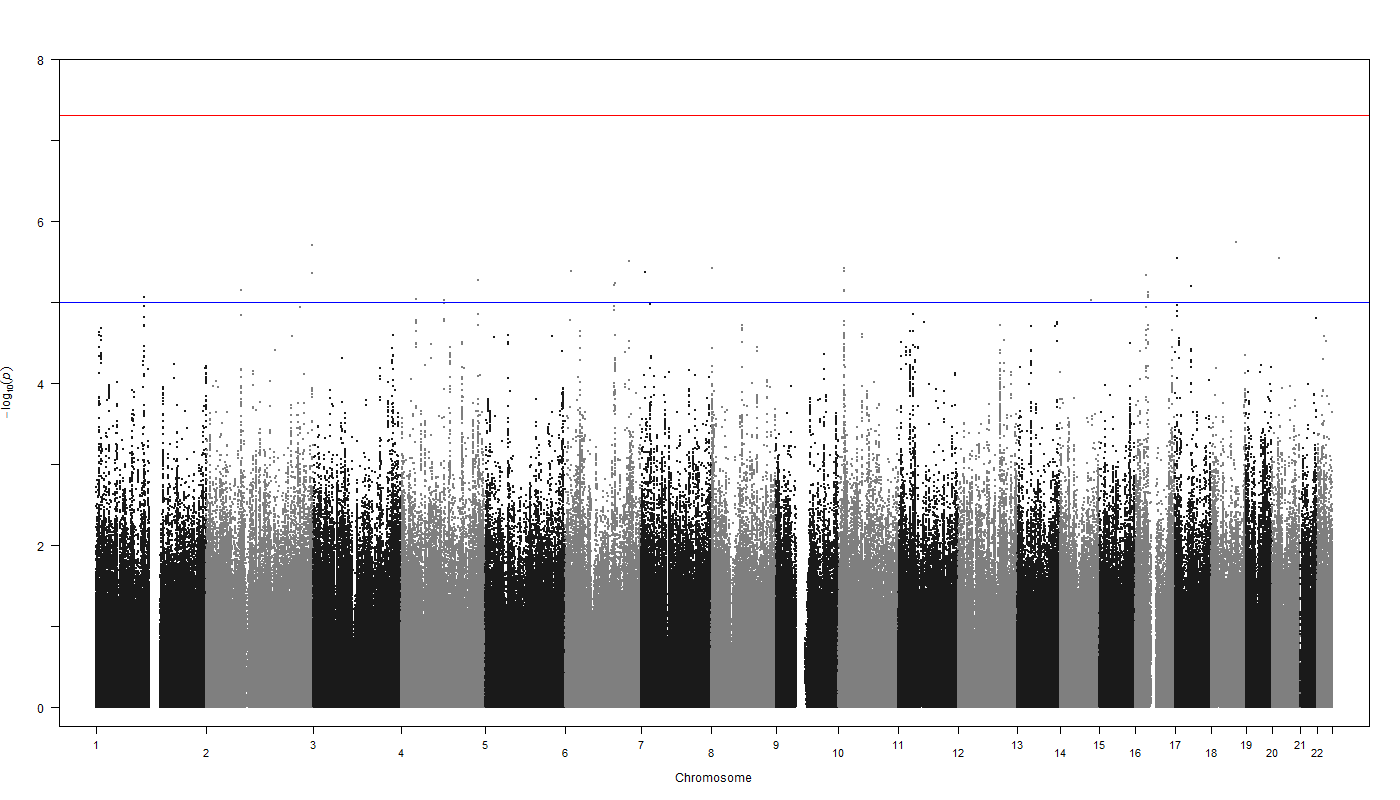


FTC NEO


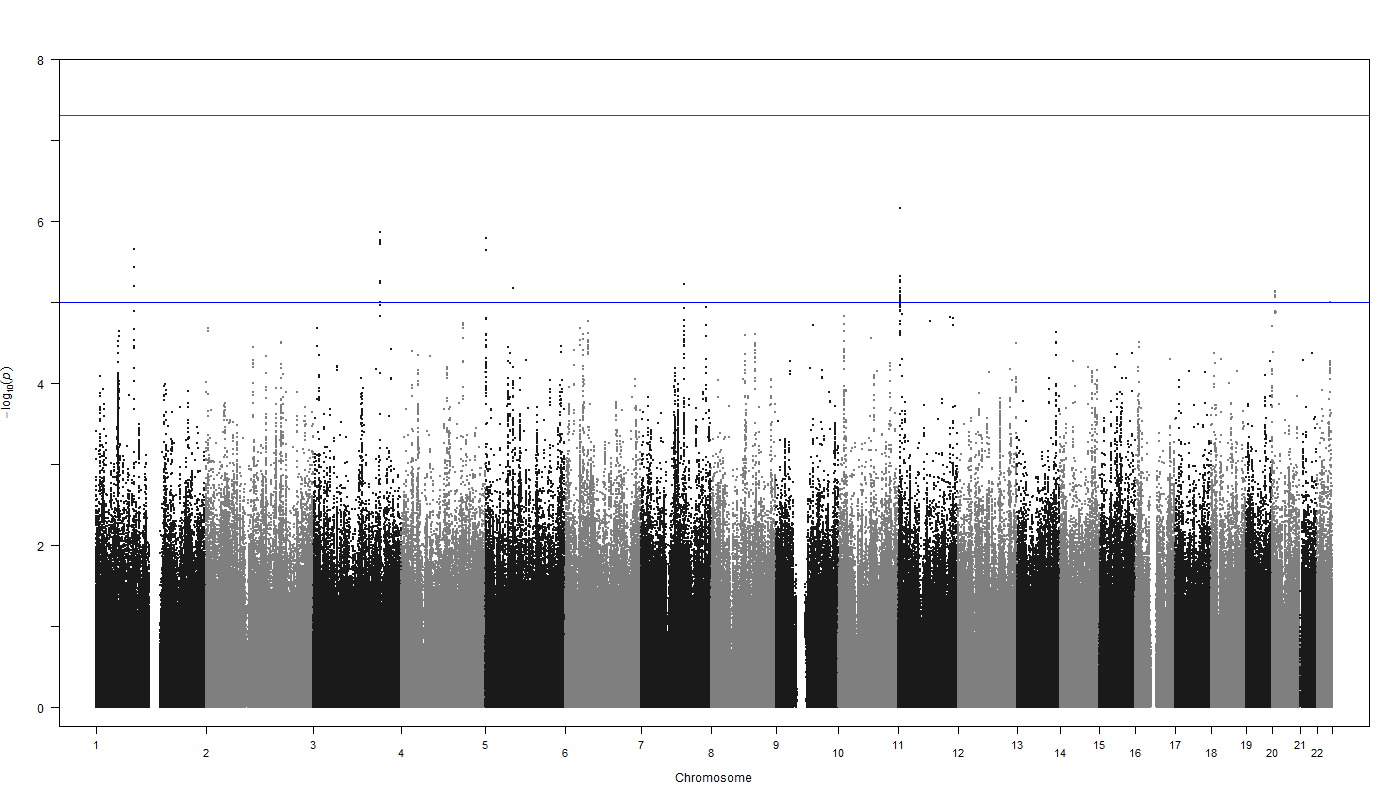


HBCS


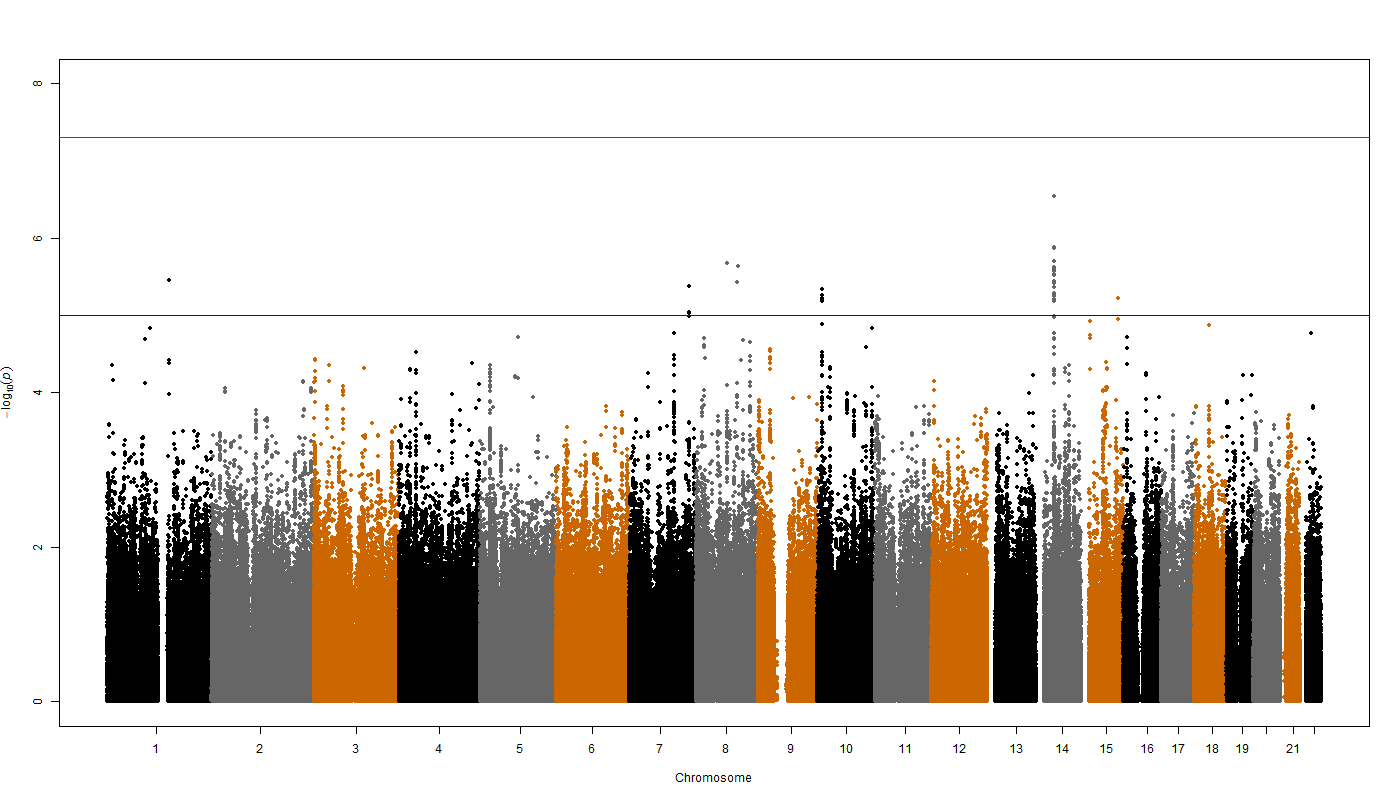


CROATIA-Korcula


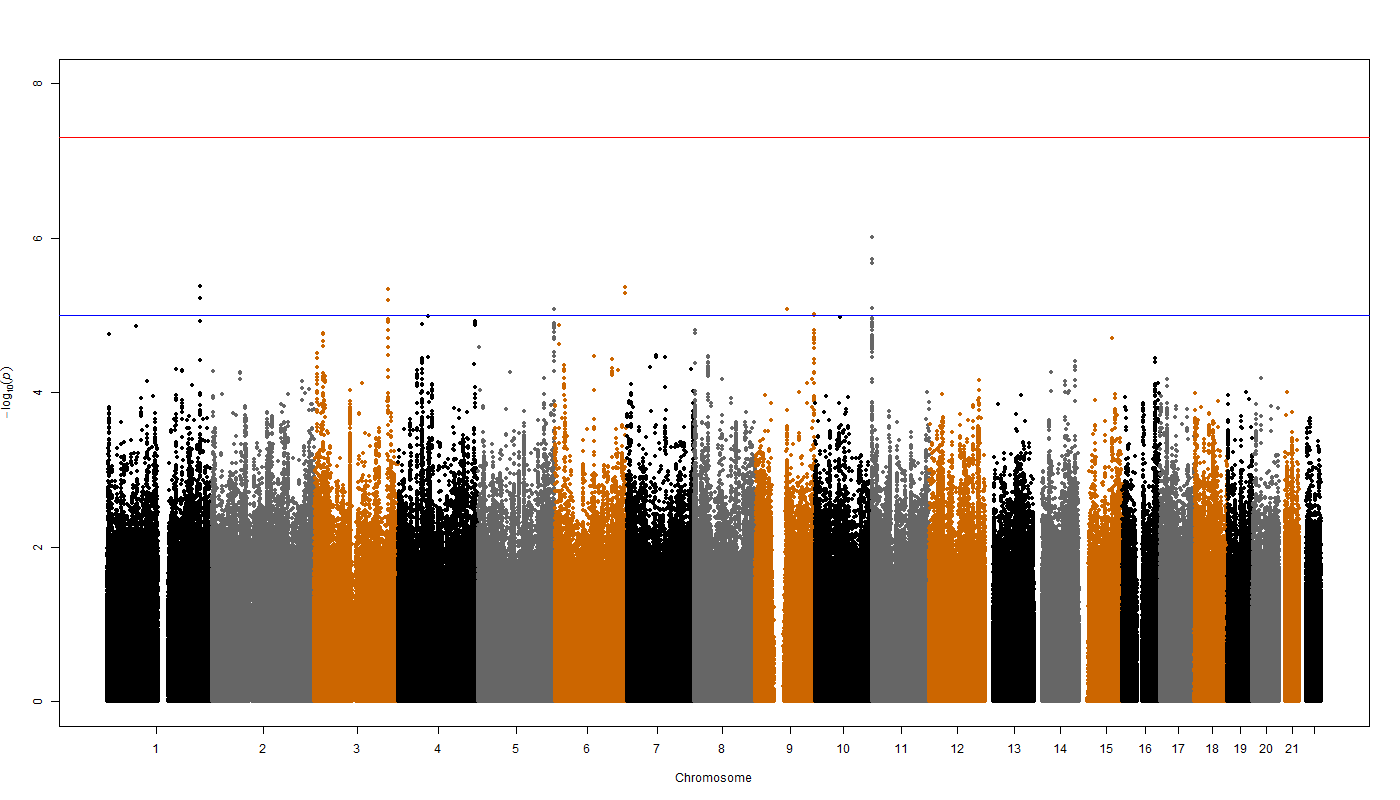


LBC1921


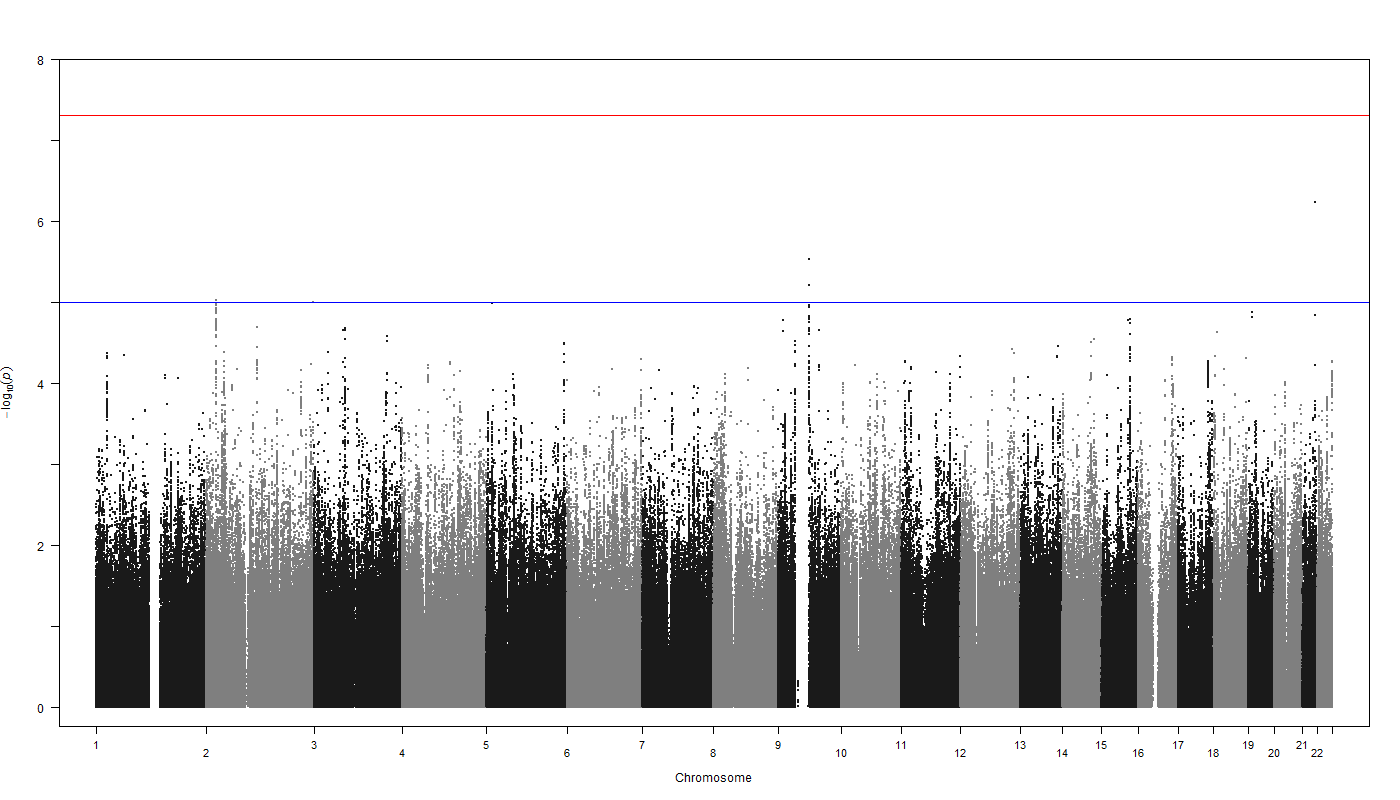


LBC1936


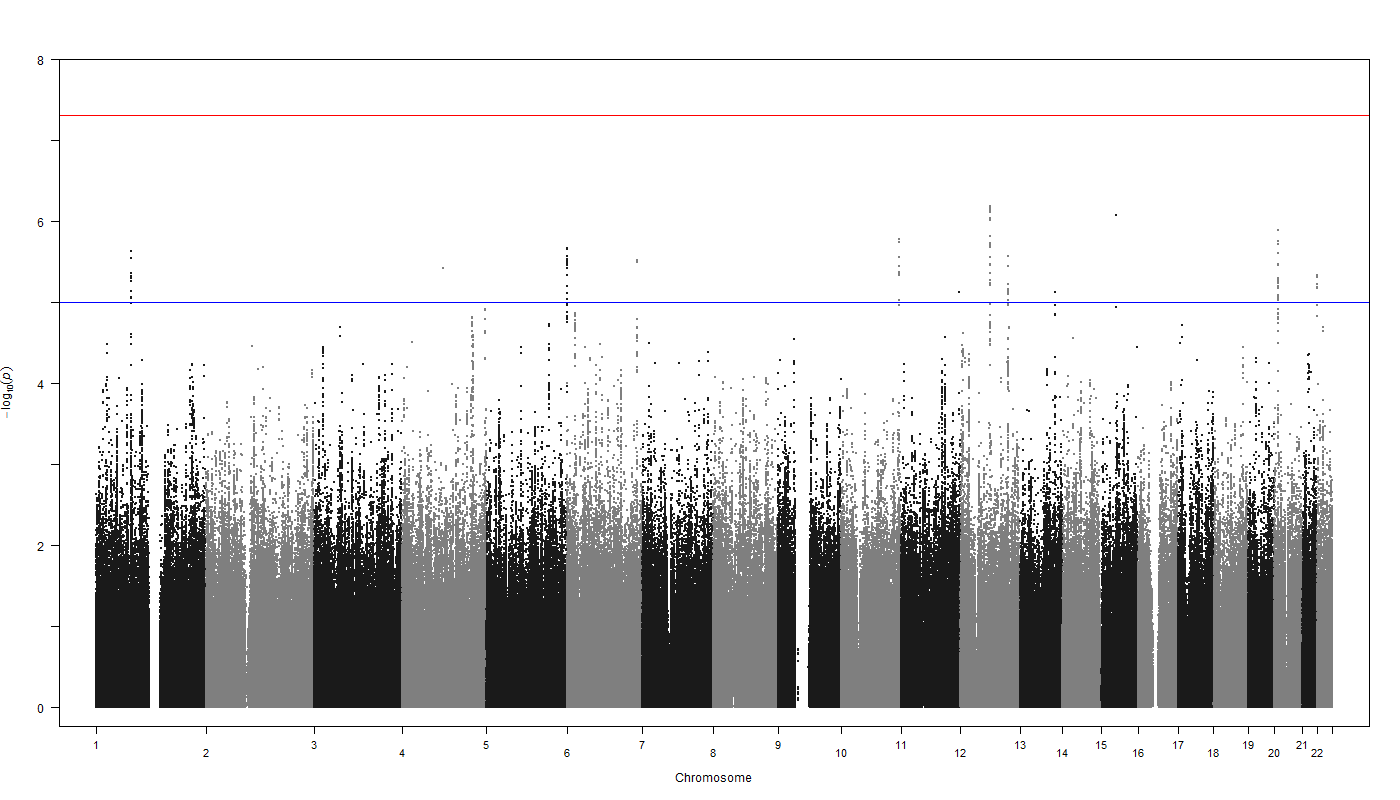


MCTFR


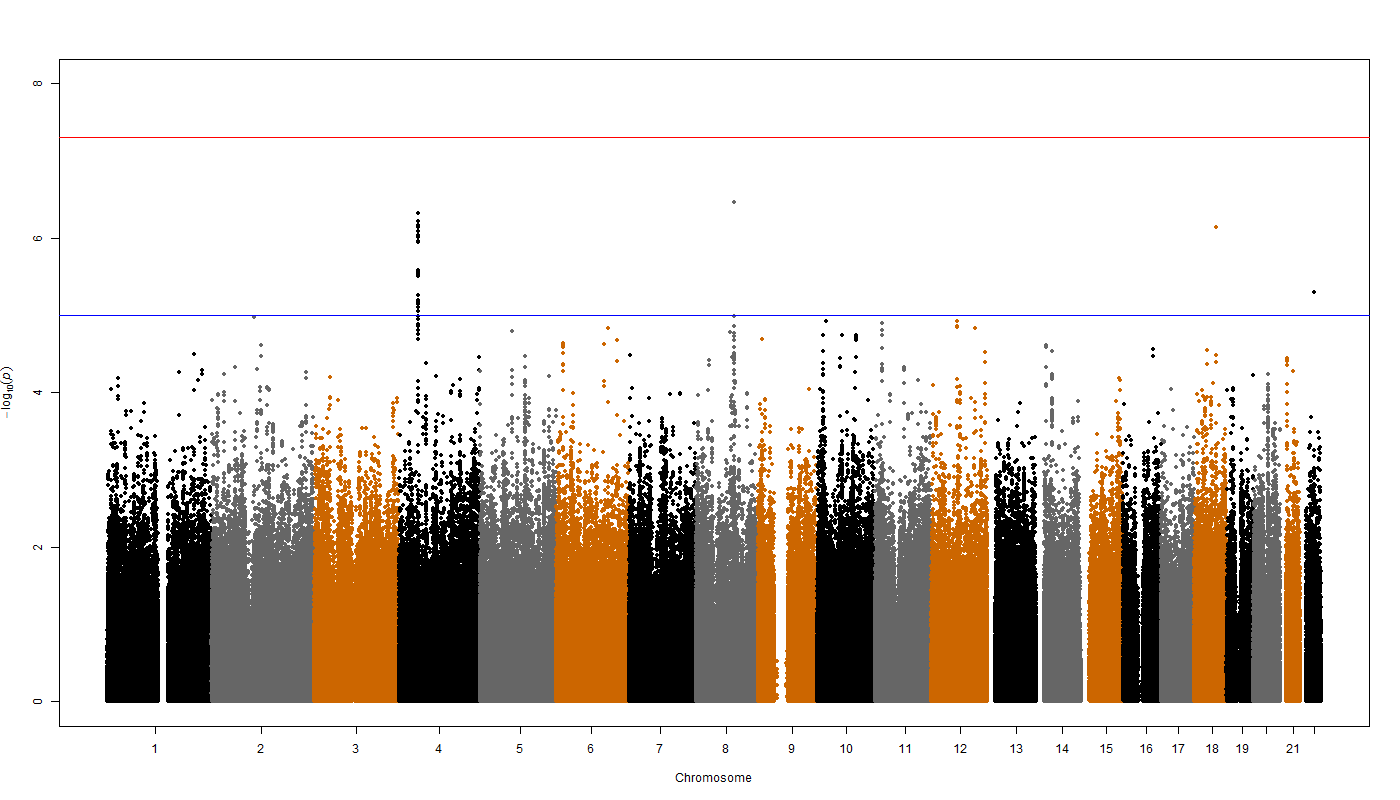


MGS


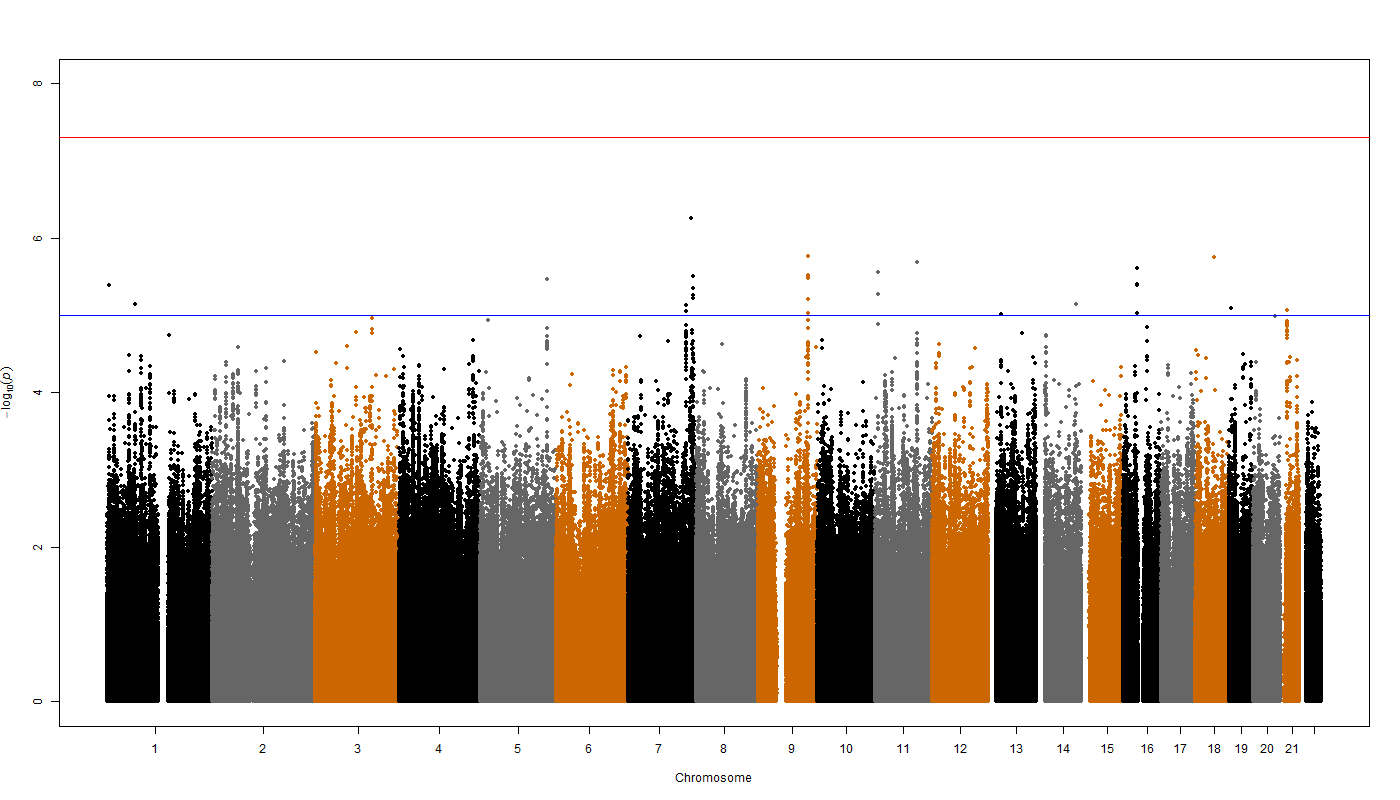


NBS


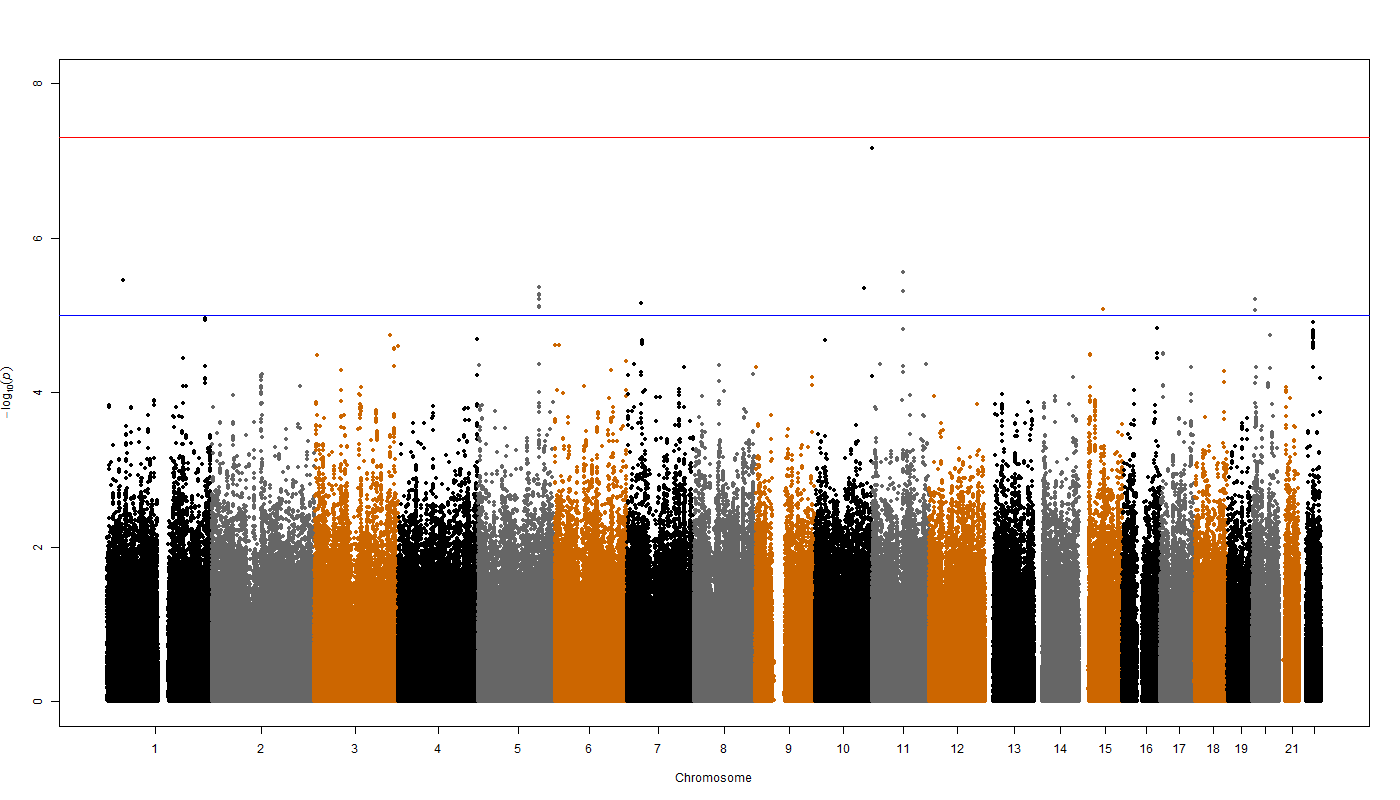


NESDA


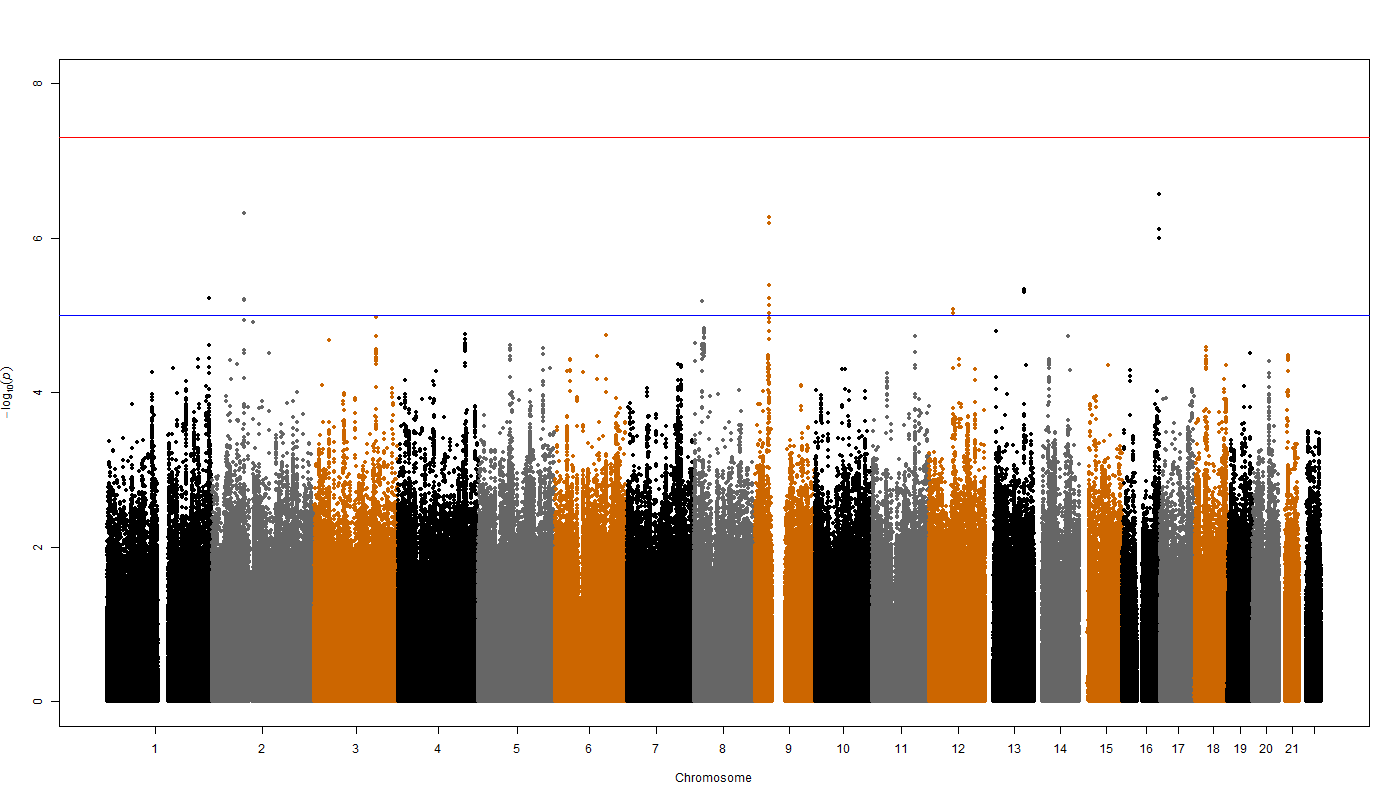


NTR


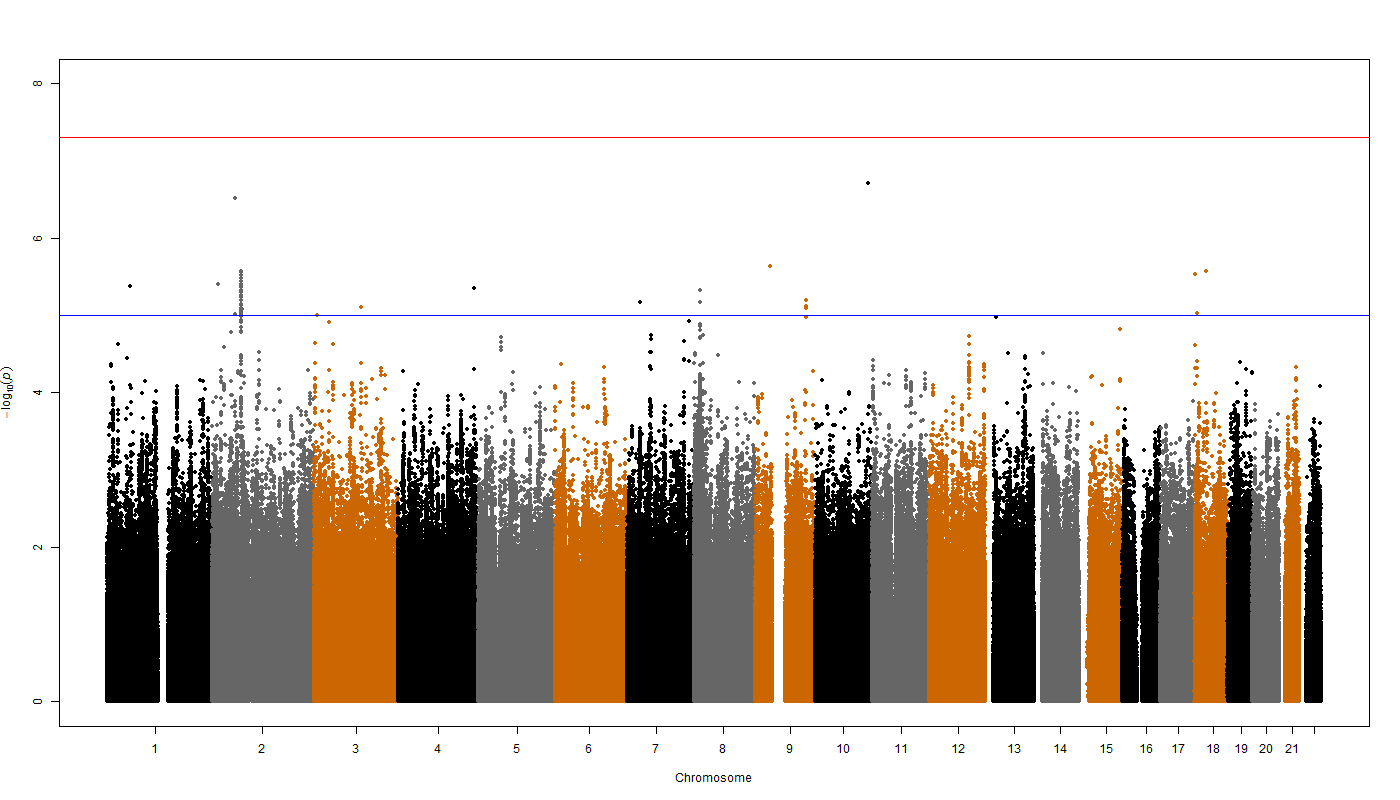


ORCADES


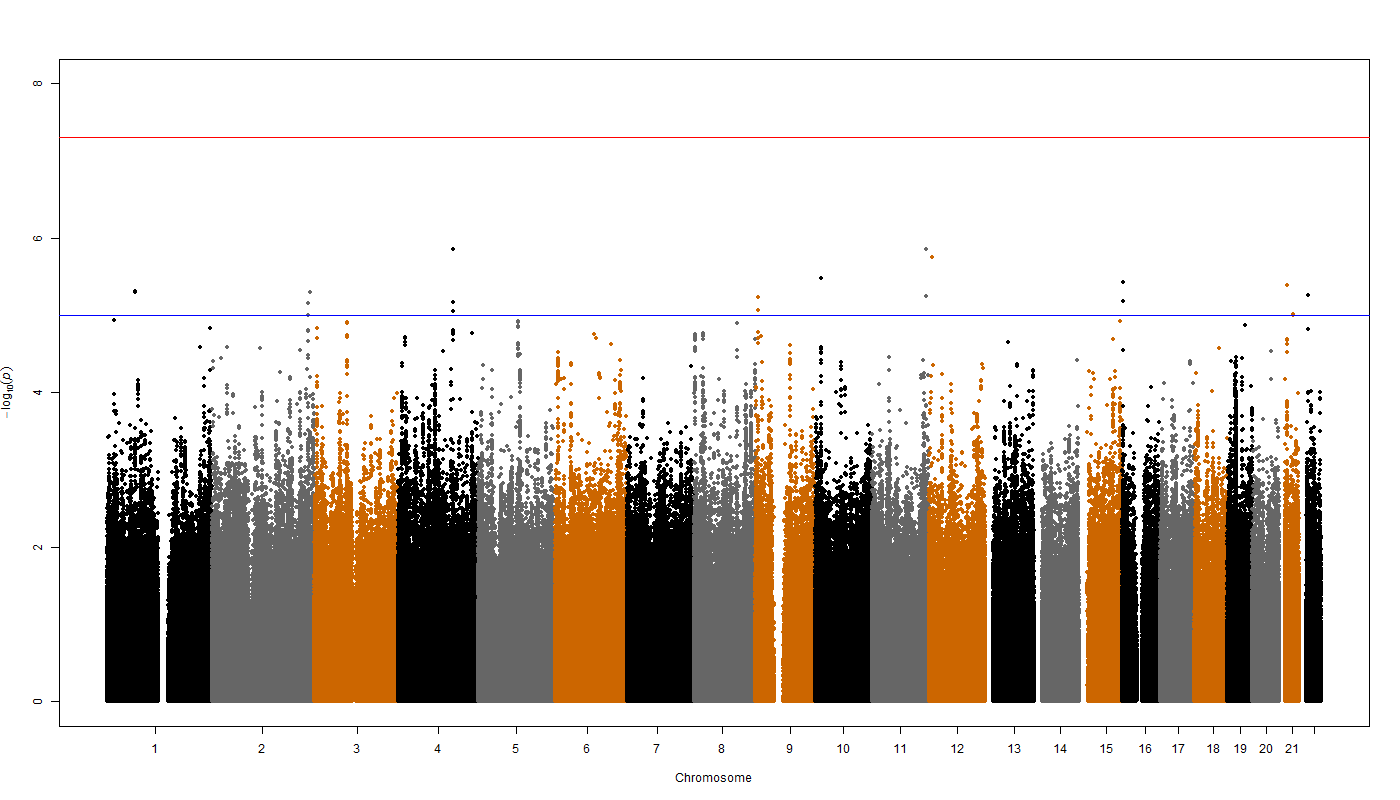


PAGES


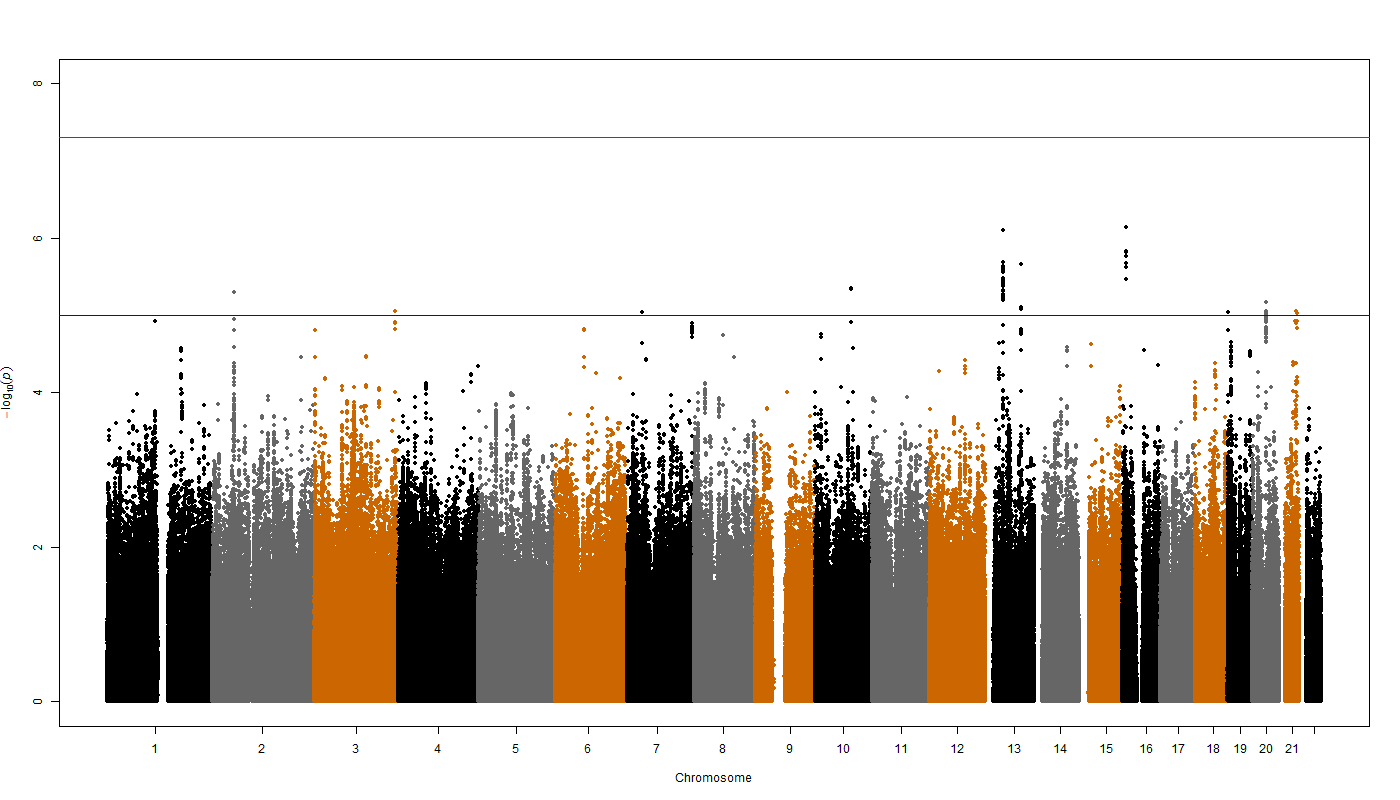


QIMR adolescents


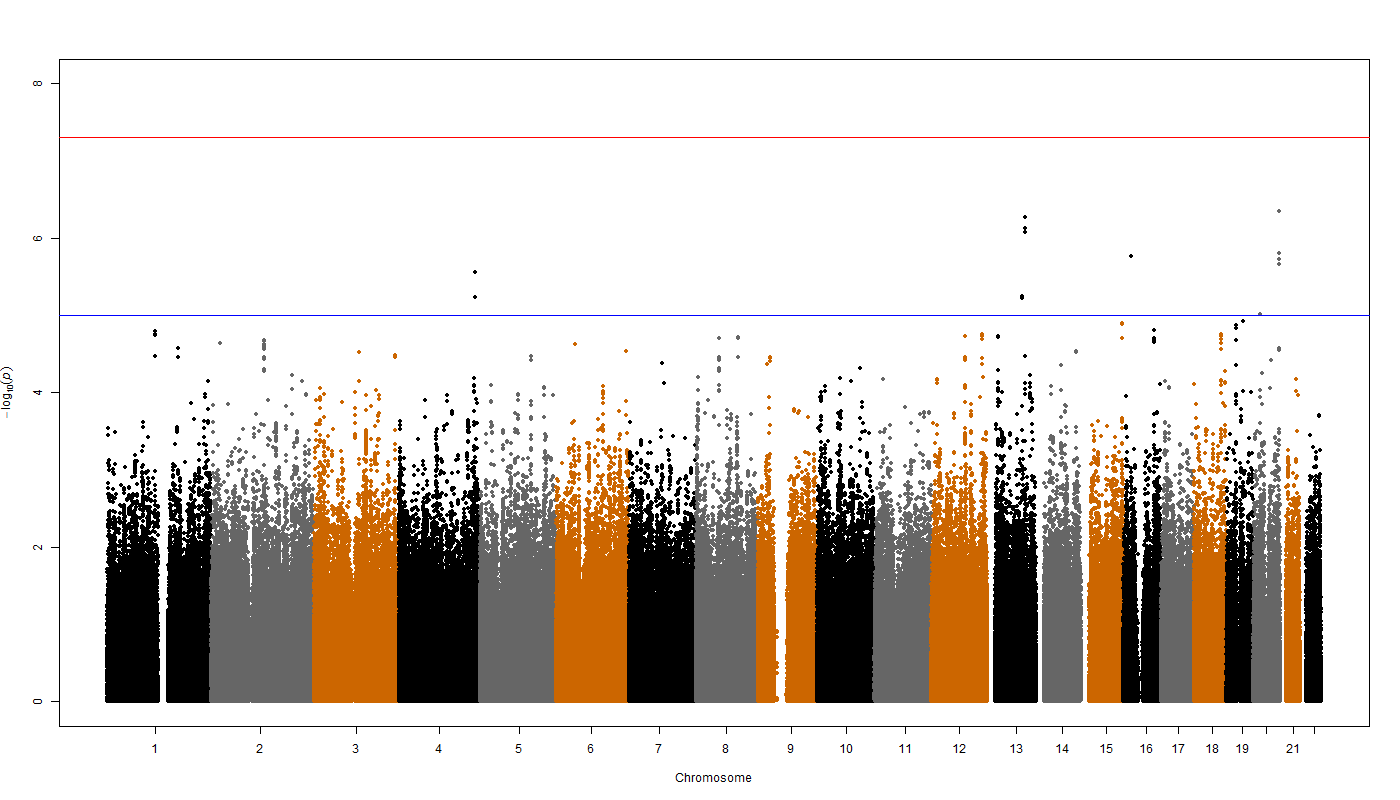


QIMR adults


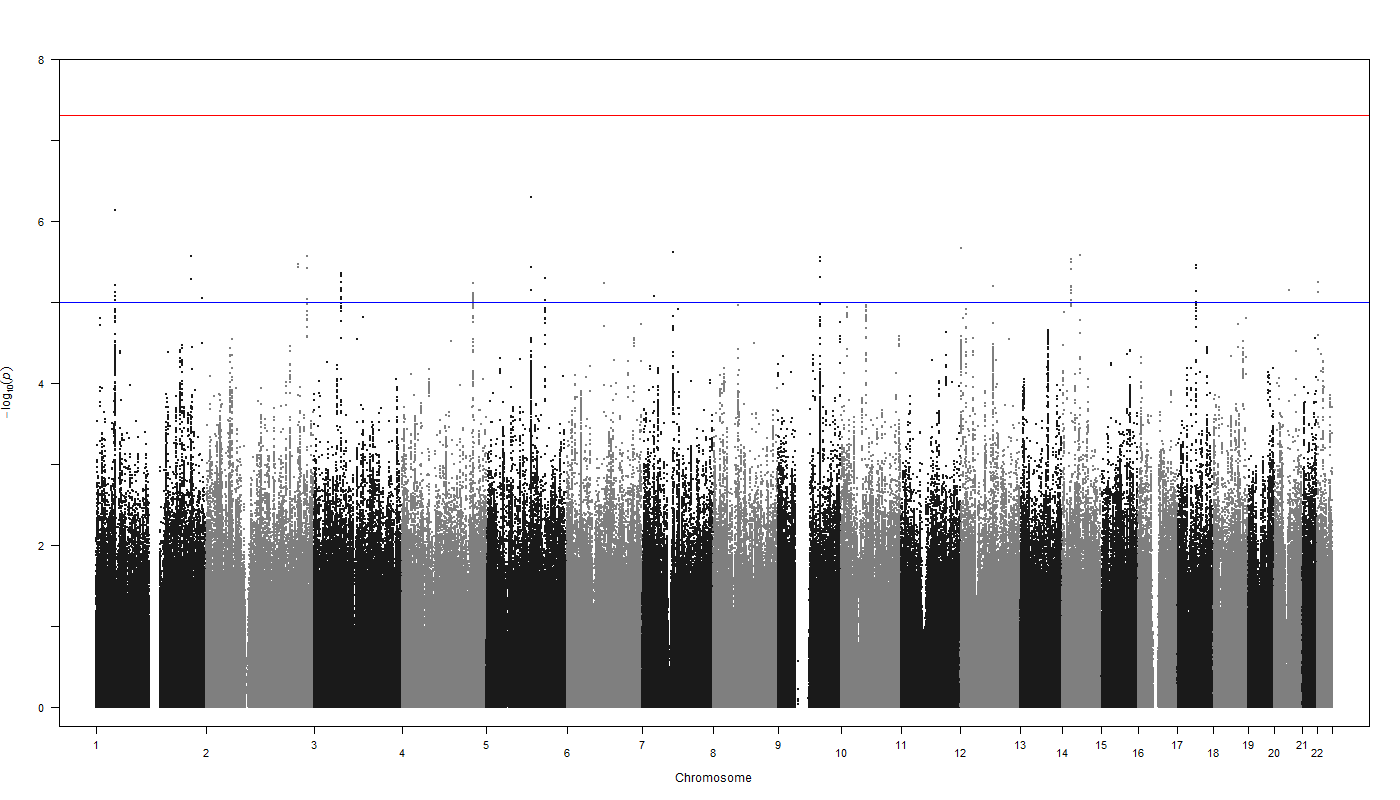


SAGE-COGA


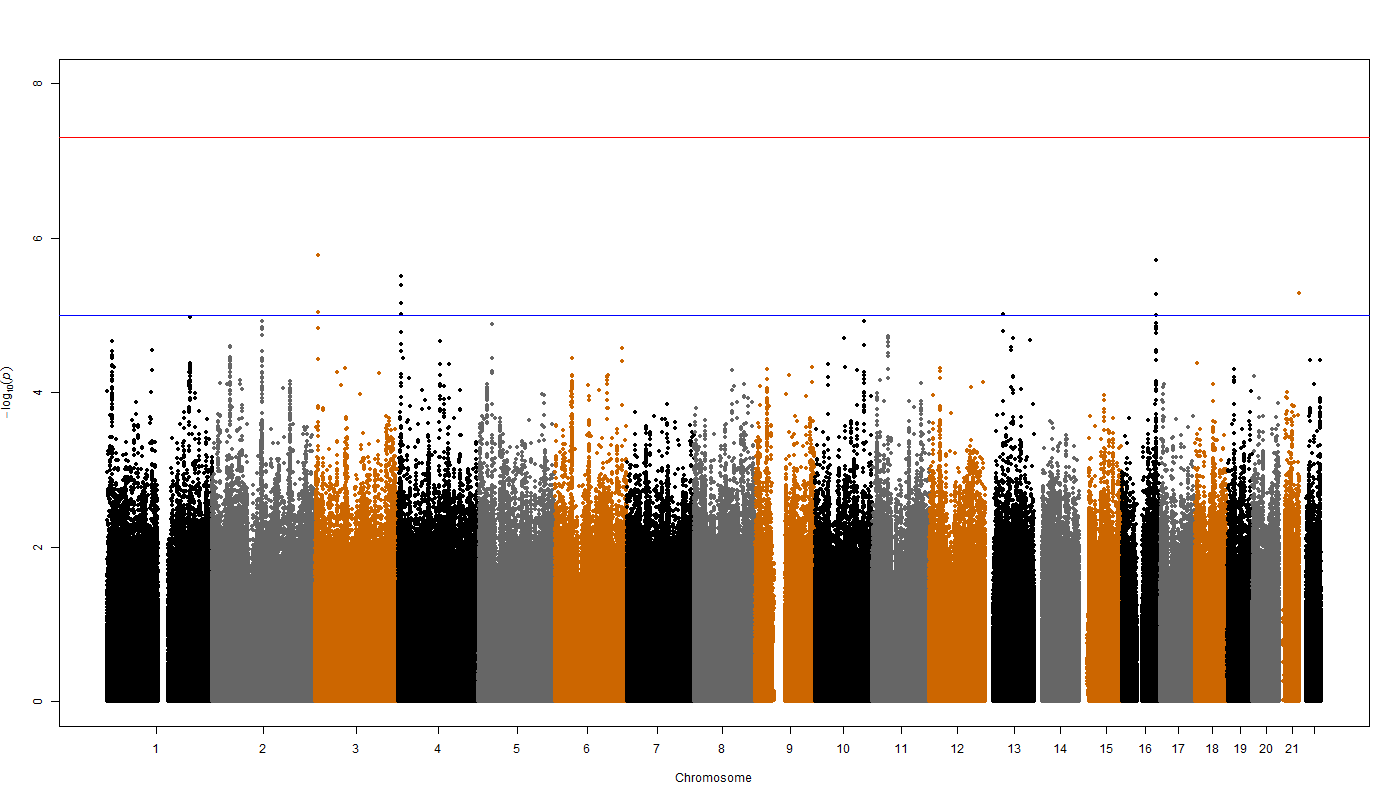


SAGE-COGEND


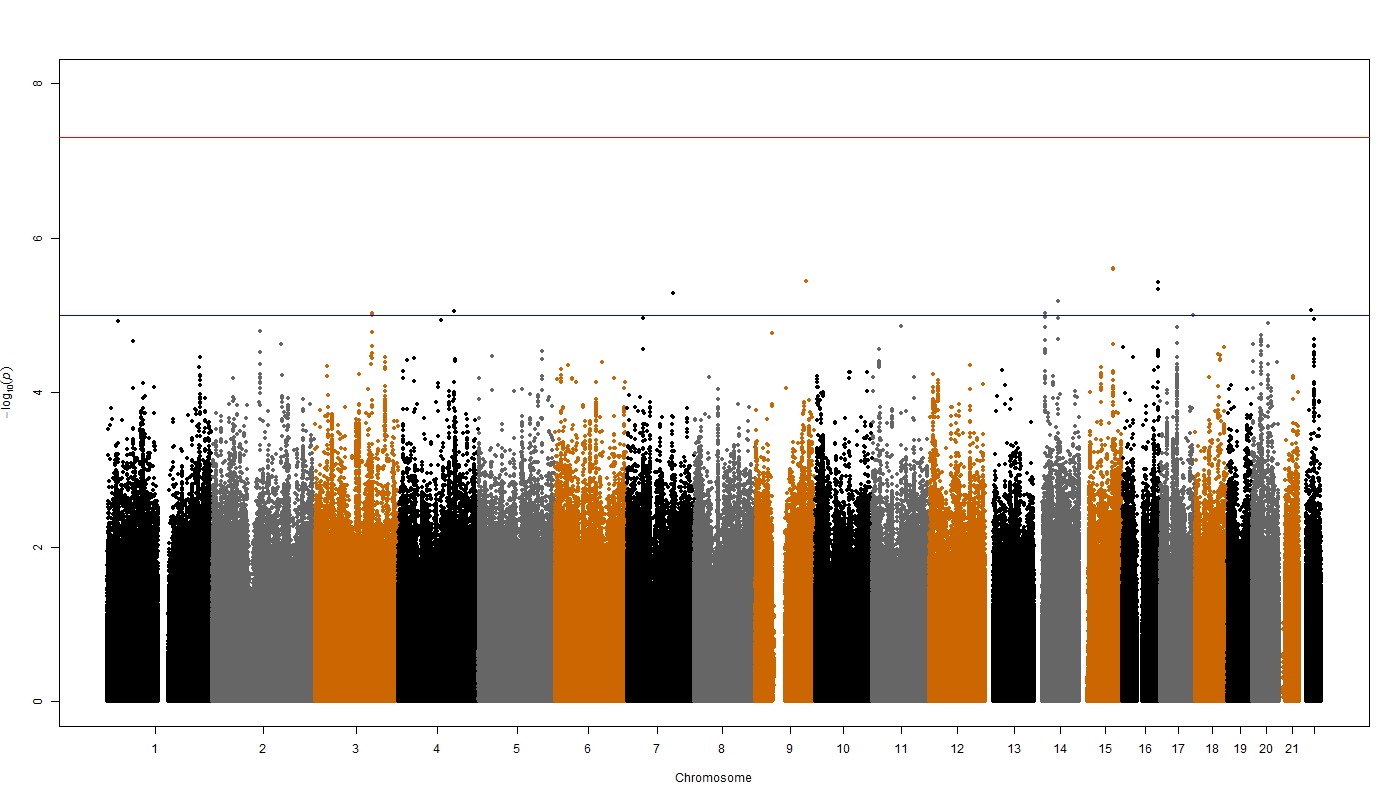


SardiNIA


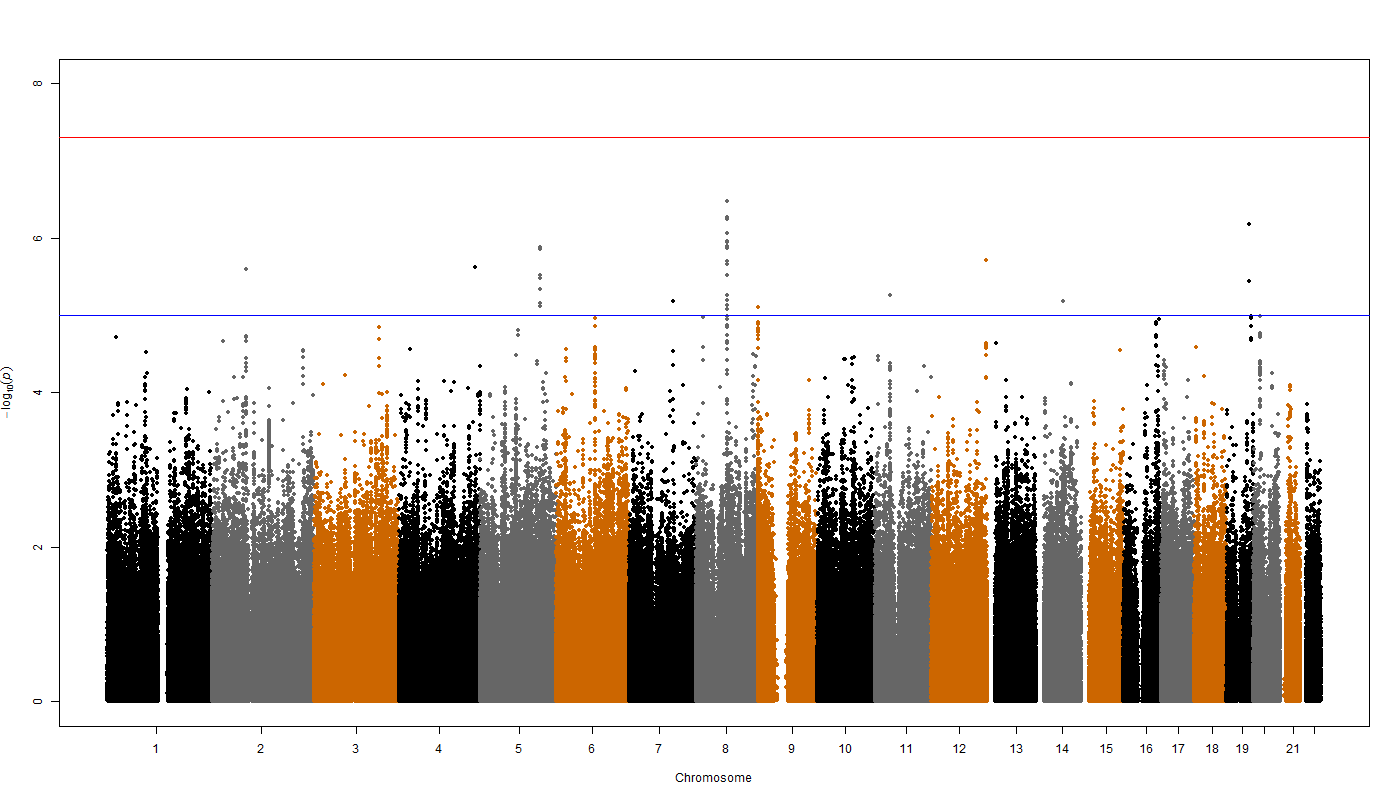


SHIP


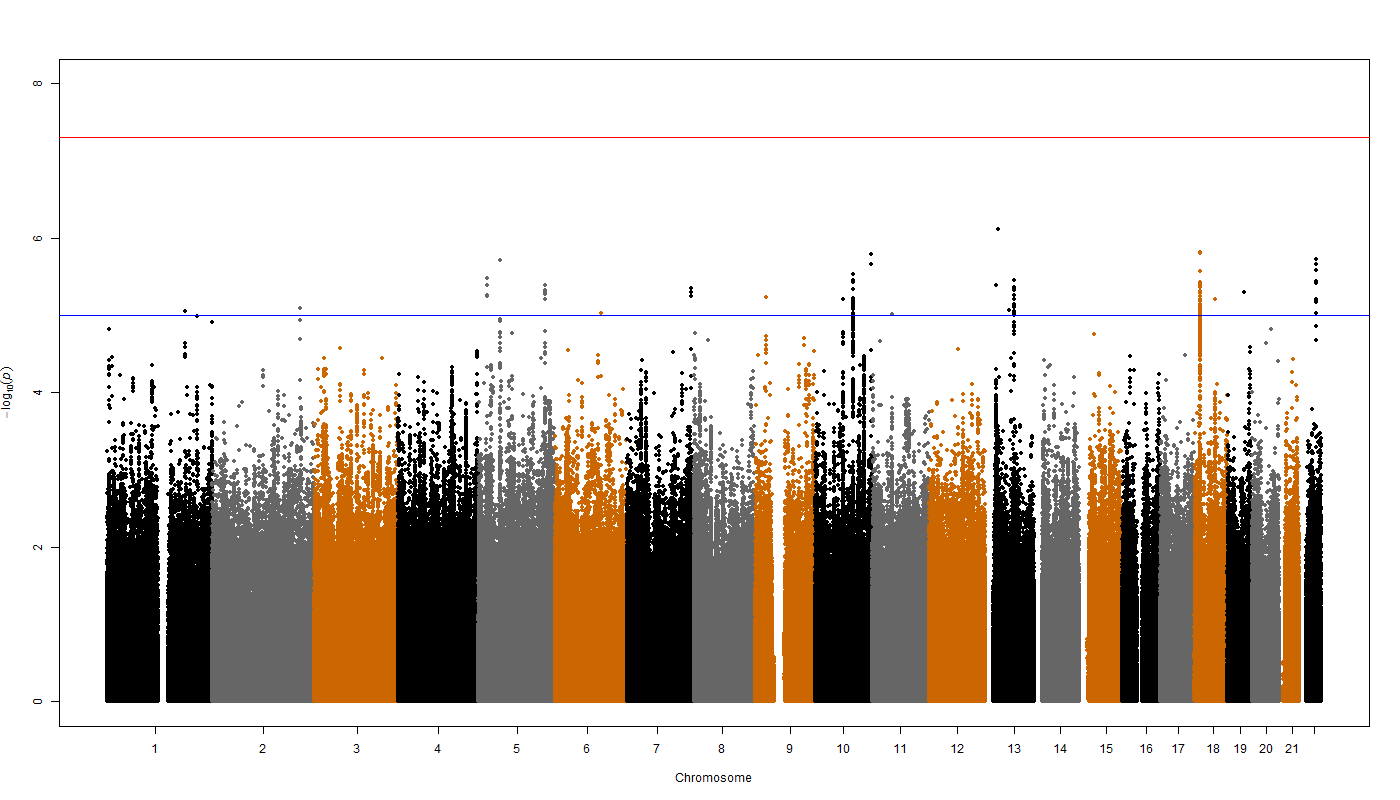


STR


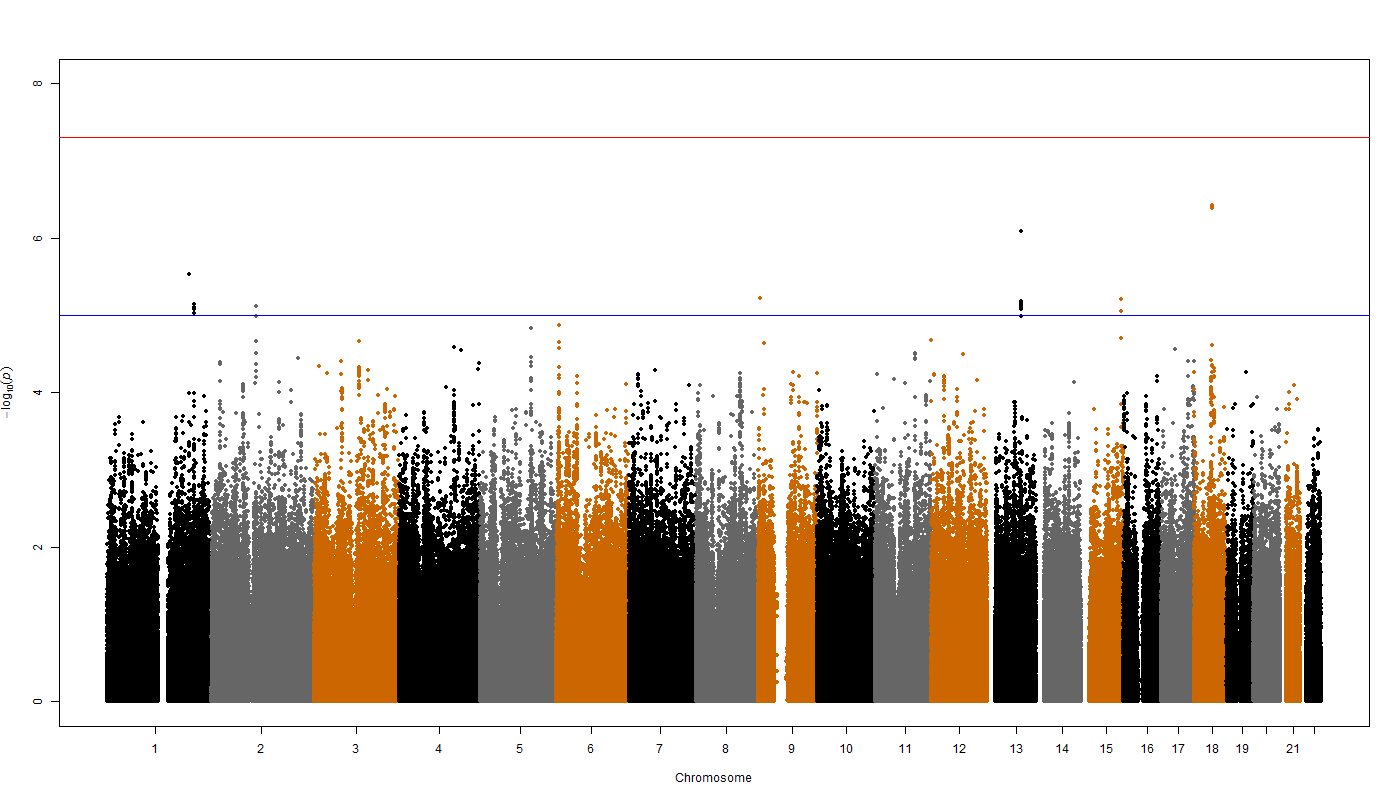


CROATIA-Vis


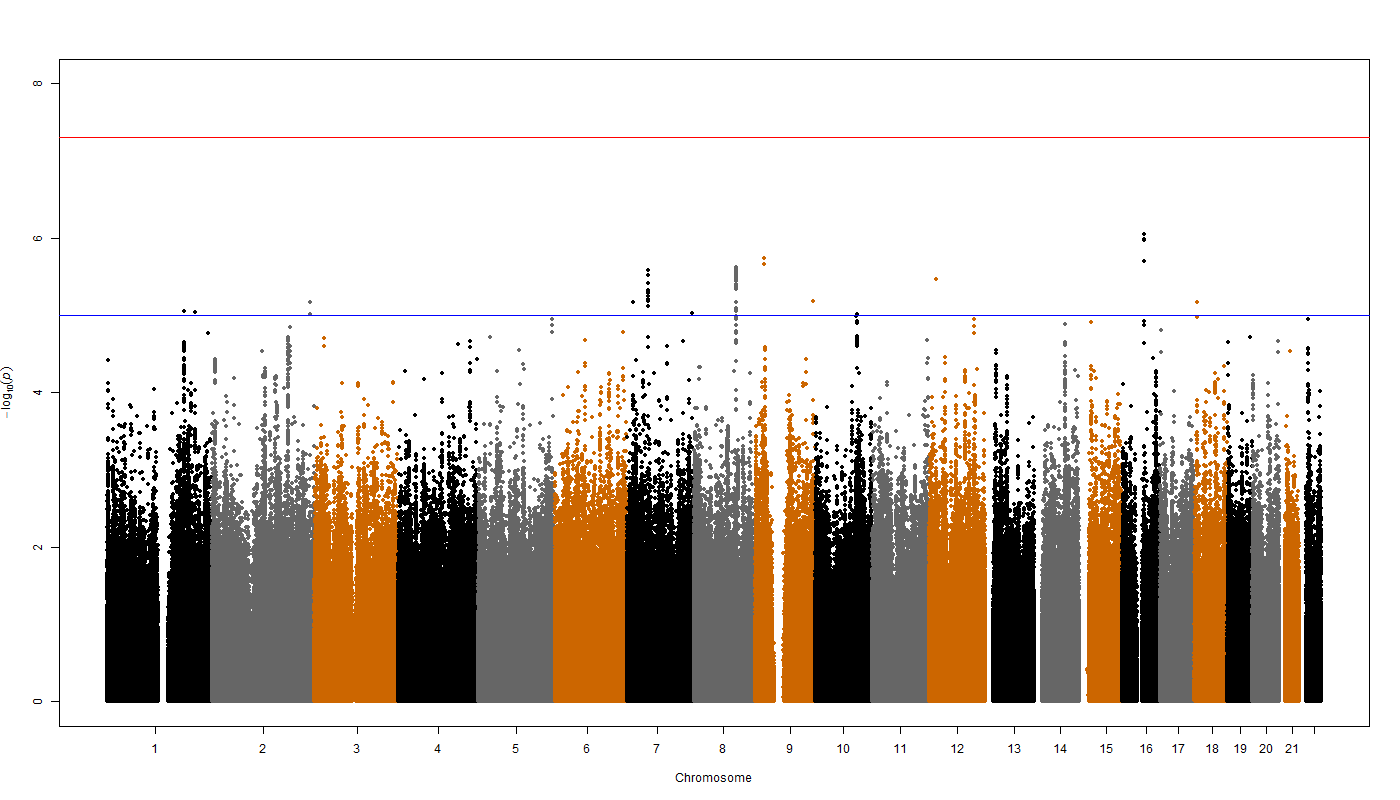


YFS


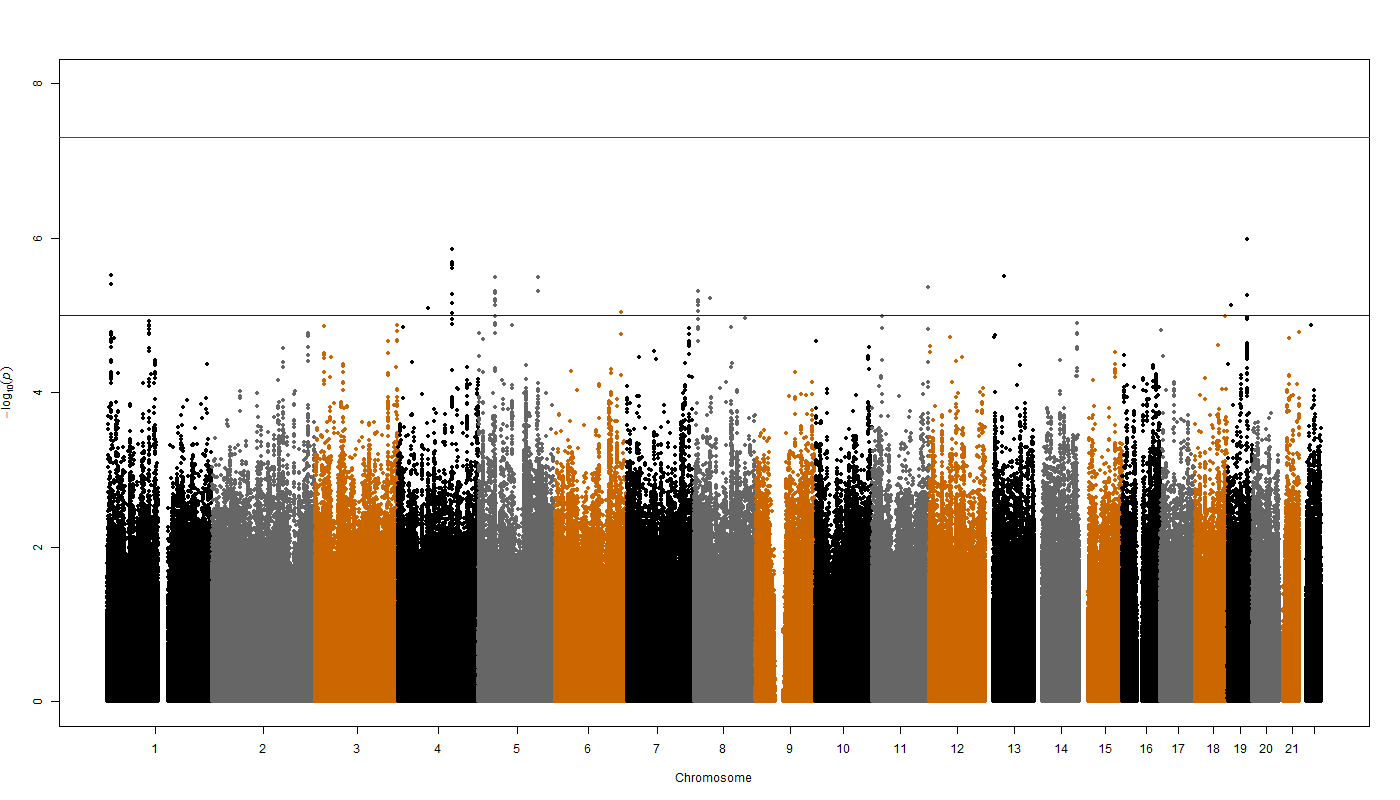


**Supplementary Figure 2.** Quantile-Quantile plots for Extraversion results in each cohort participating in the Genetic of Personality Consortium

ALSPAC


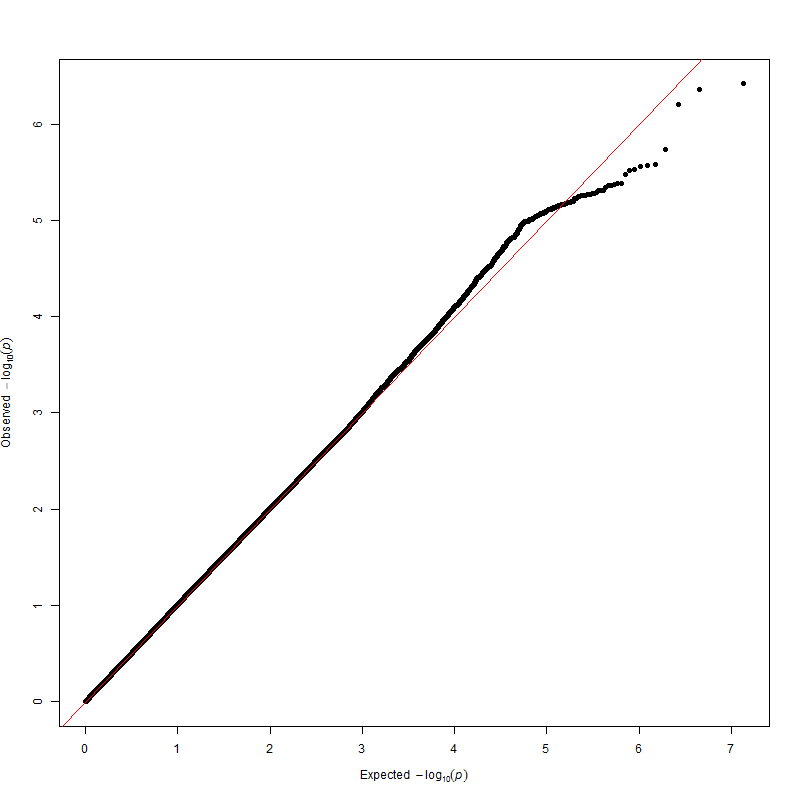


BLSA


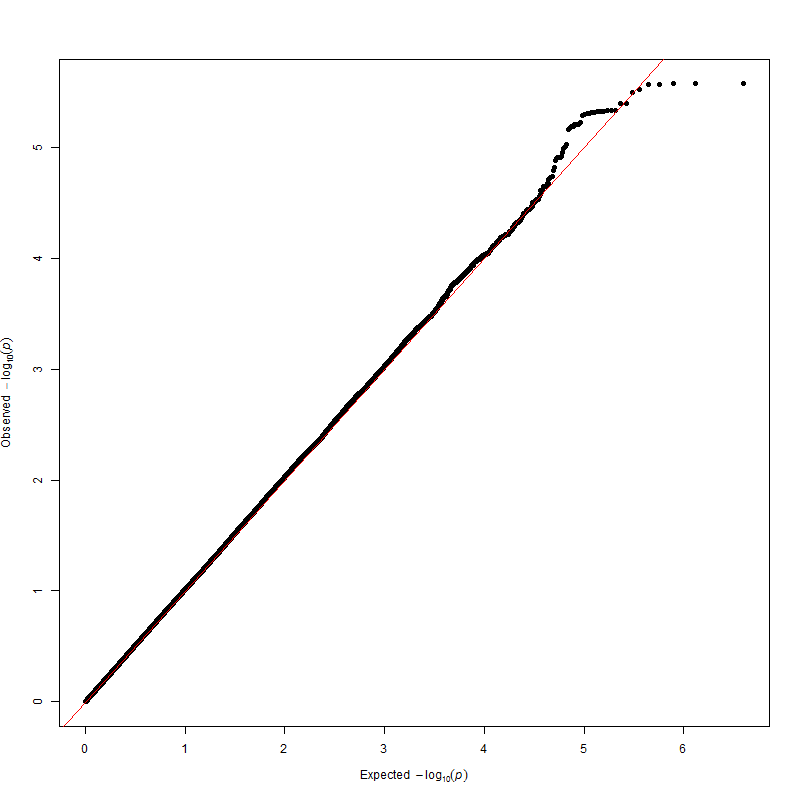


BRESCIA


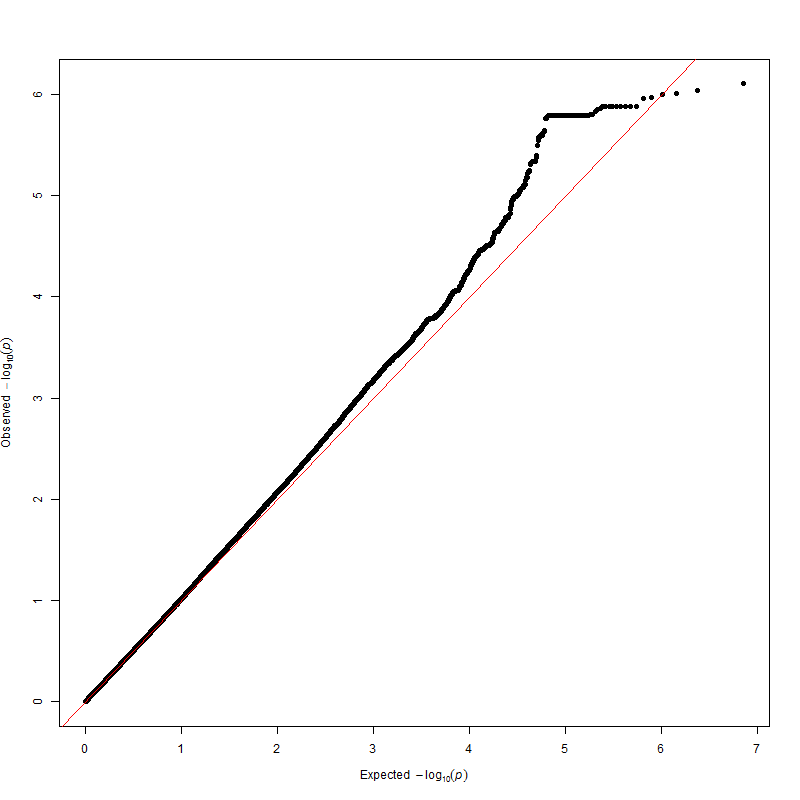


CHICAGO


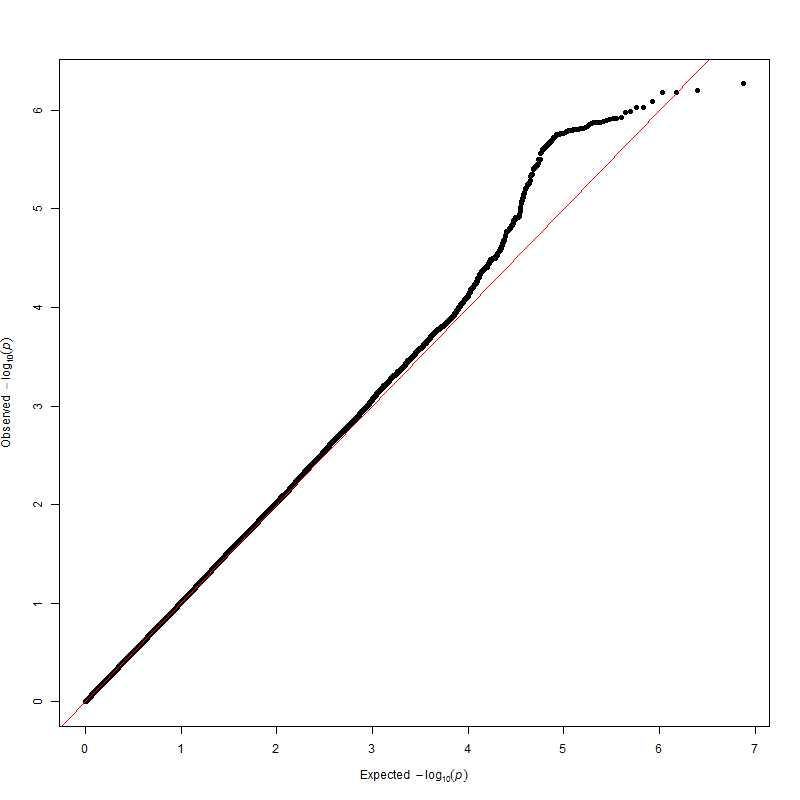


CILENTO


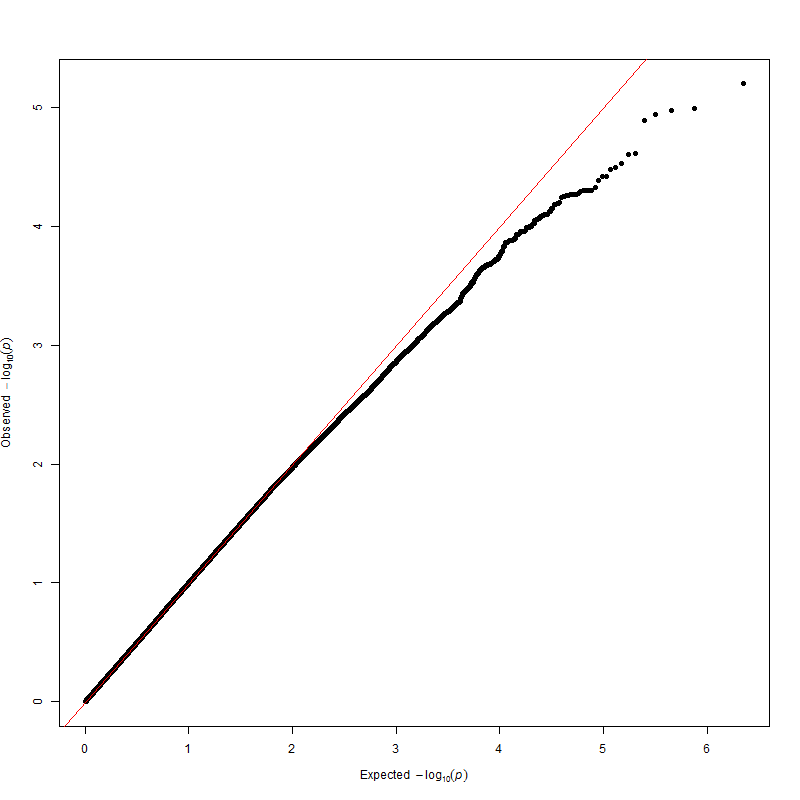


EGCUT


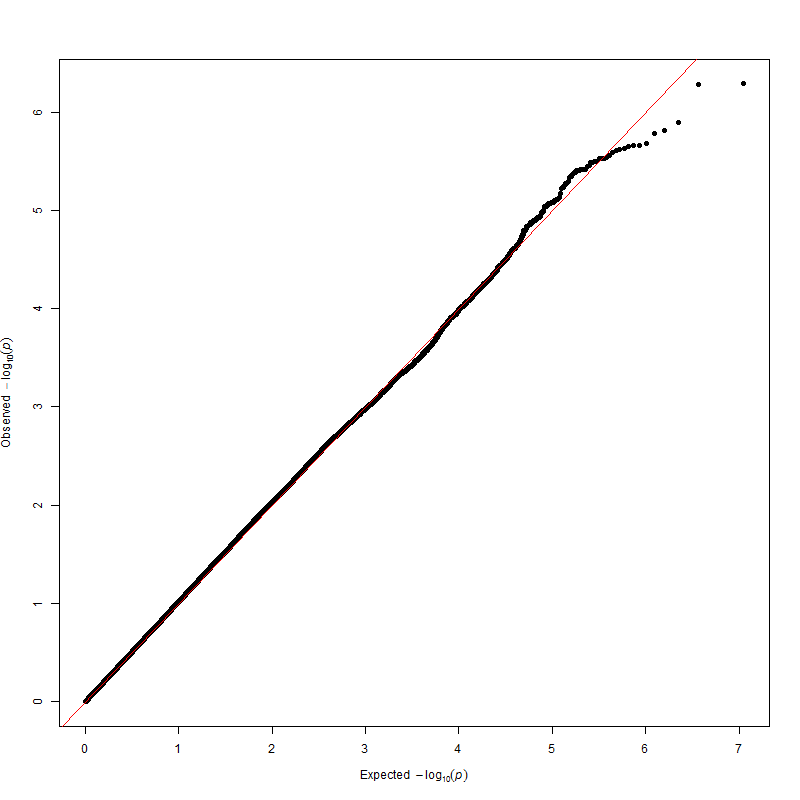


ERF


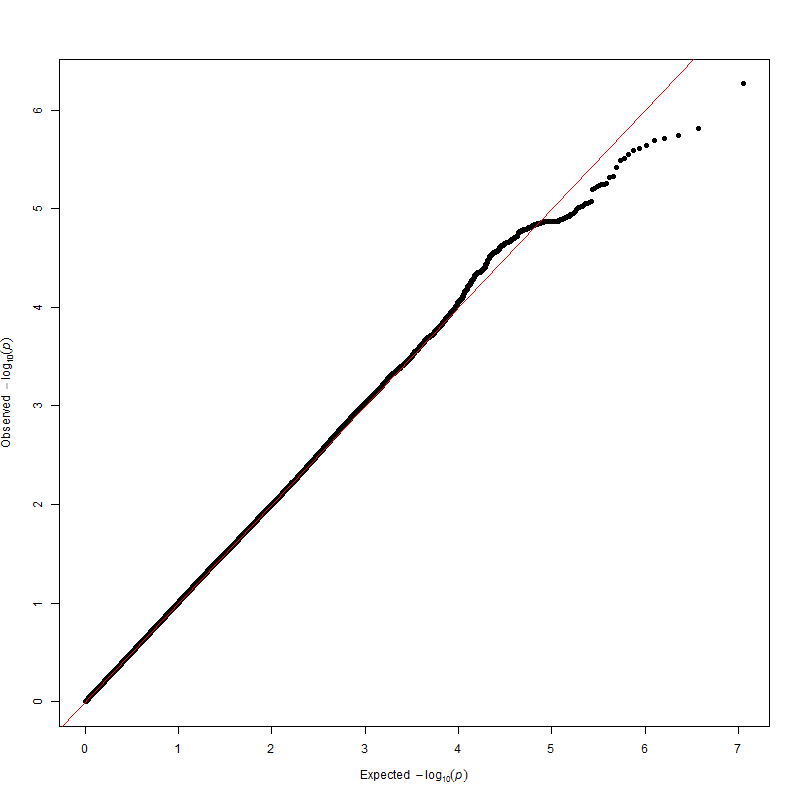


FTC EPI


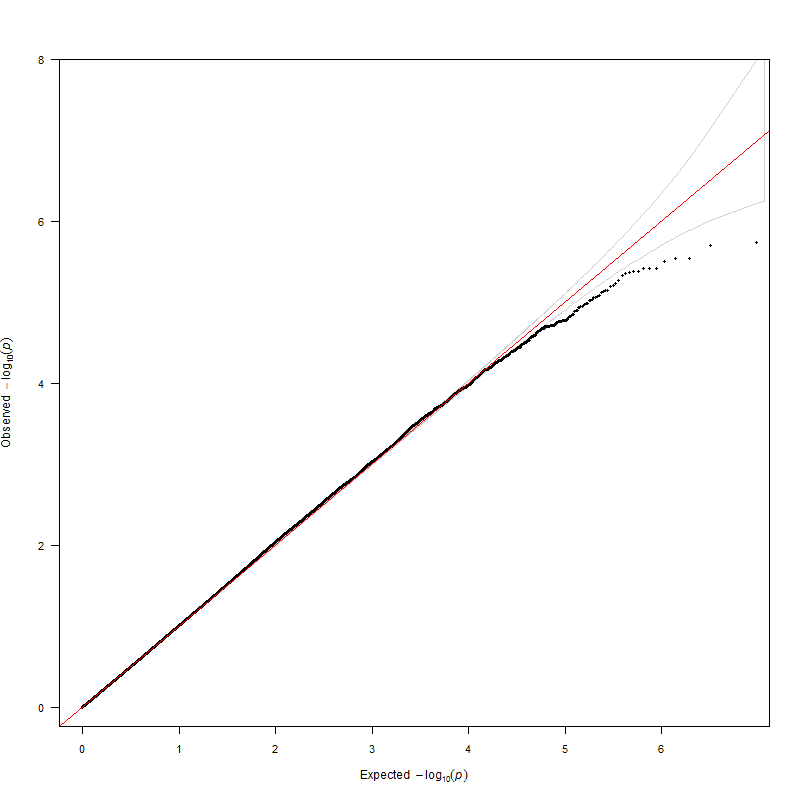


FTC NEO


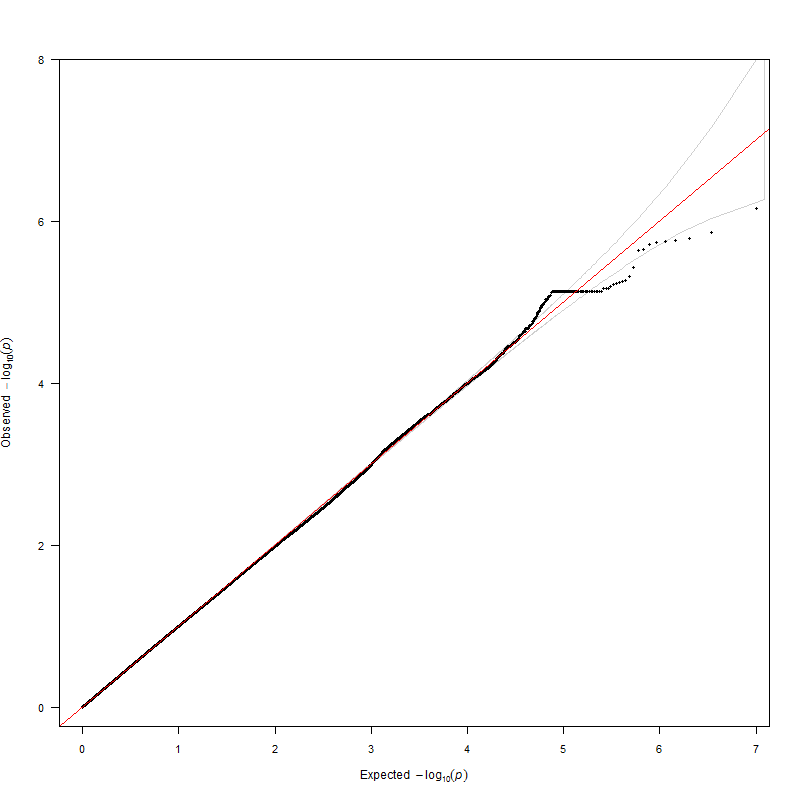


HBCS


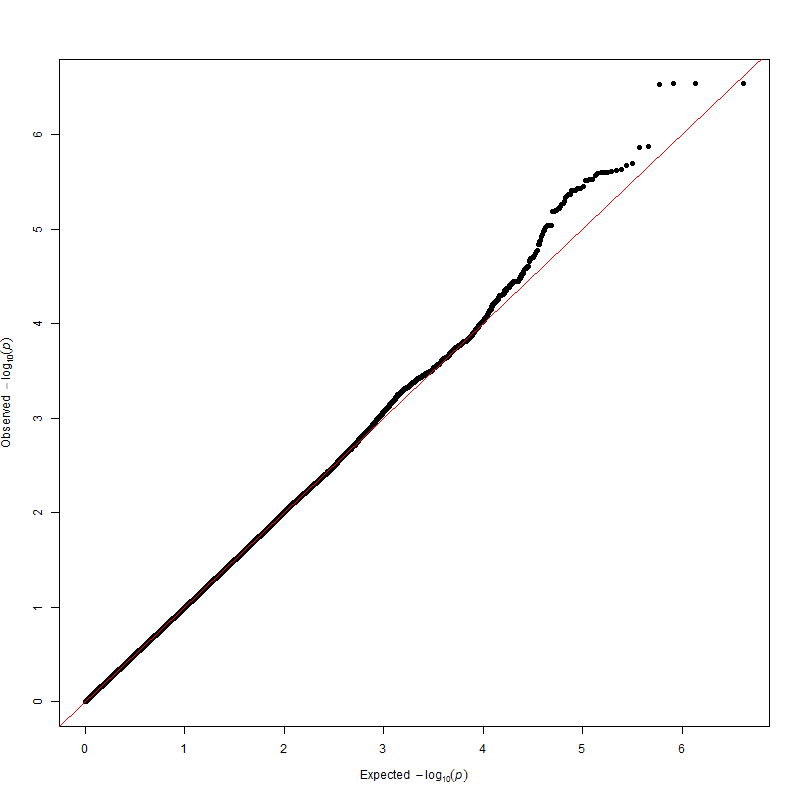


CROATIA-Korcula


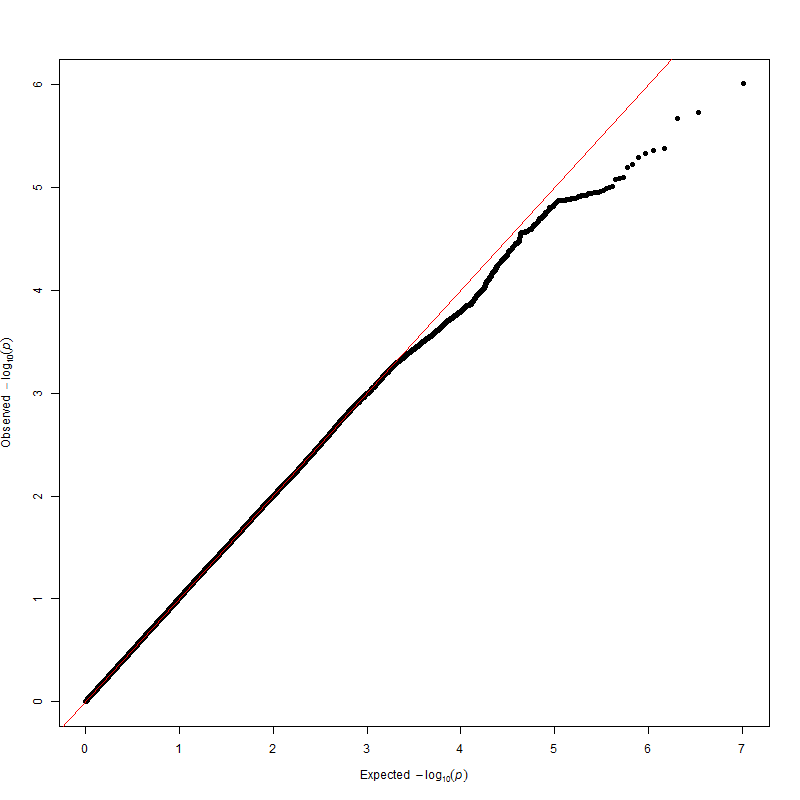


LBC1921


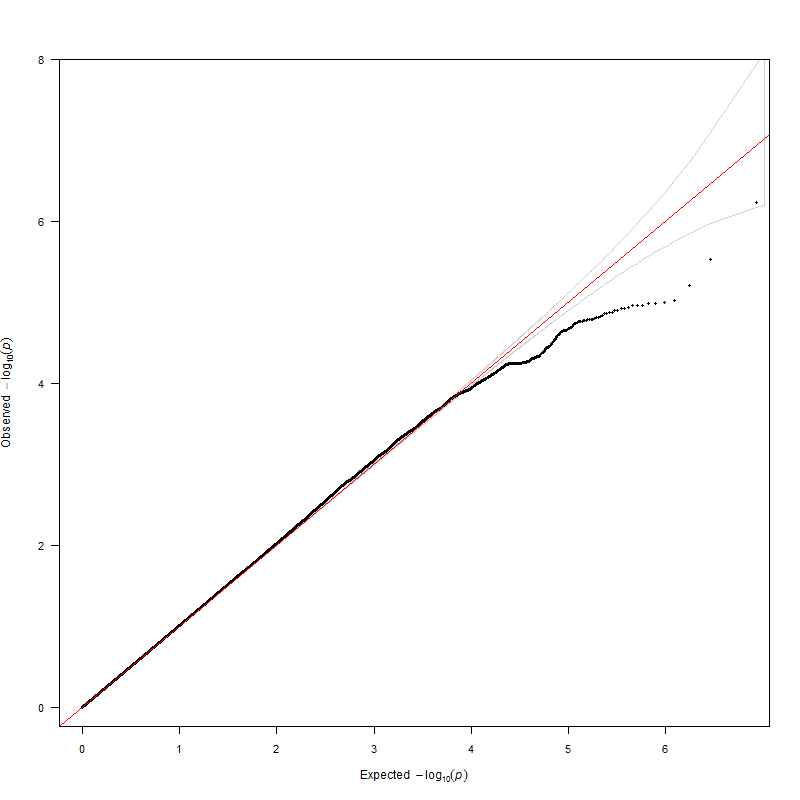


LBC1936 – Extraversion


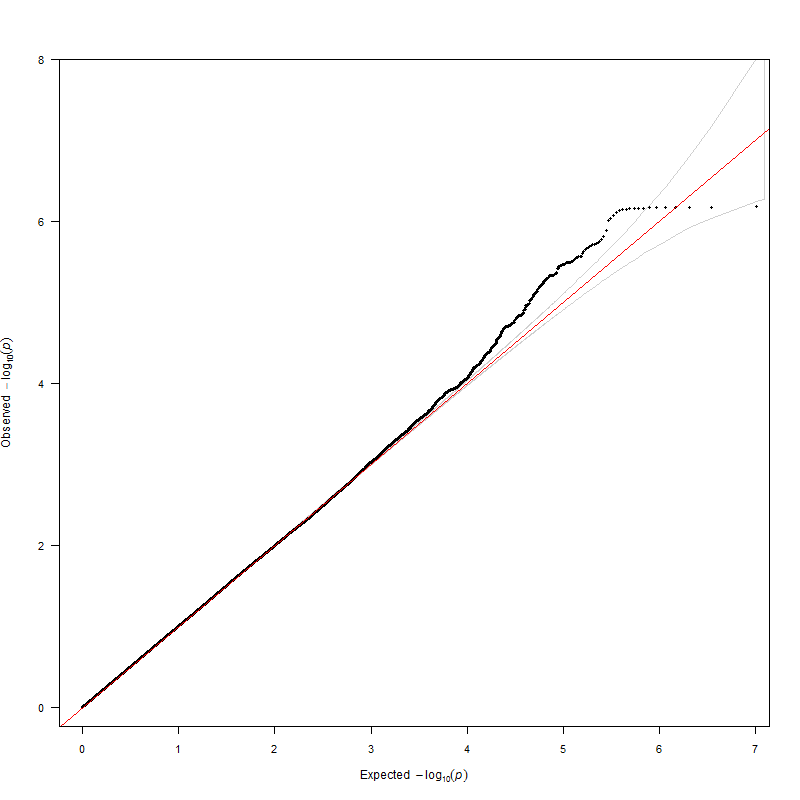


MCTFR


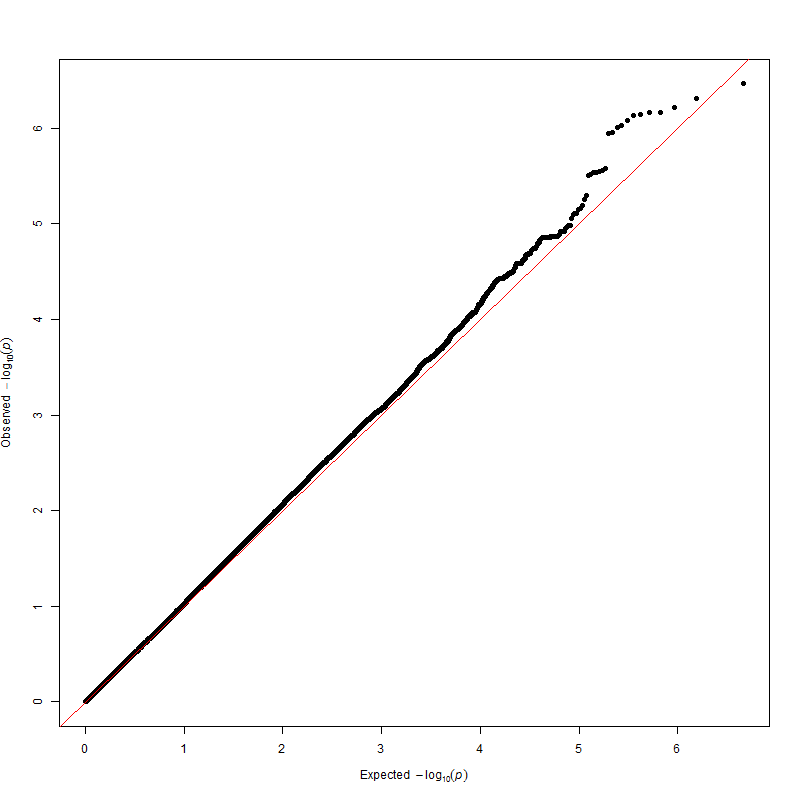


MGS


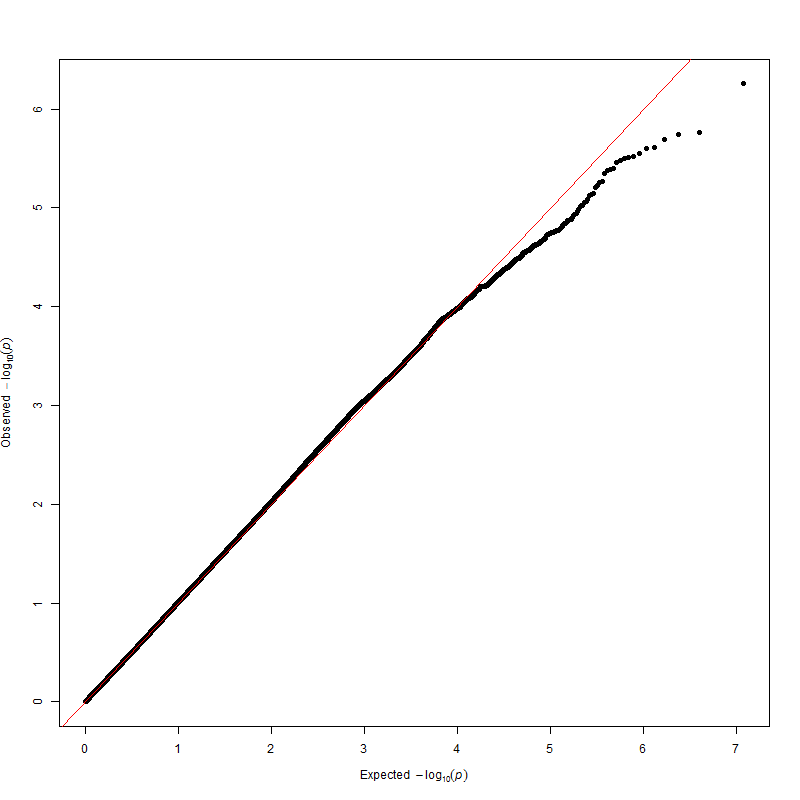


NBS


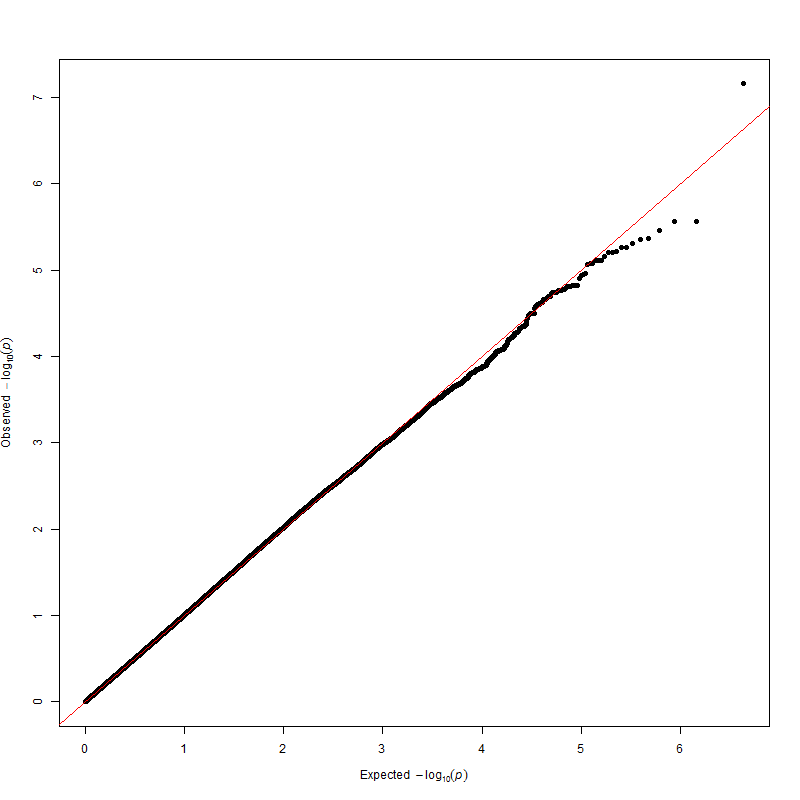


NESDA


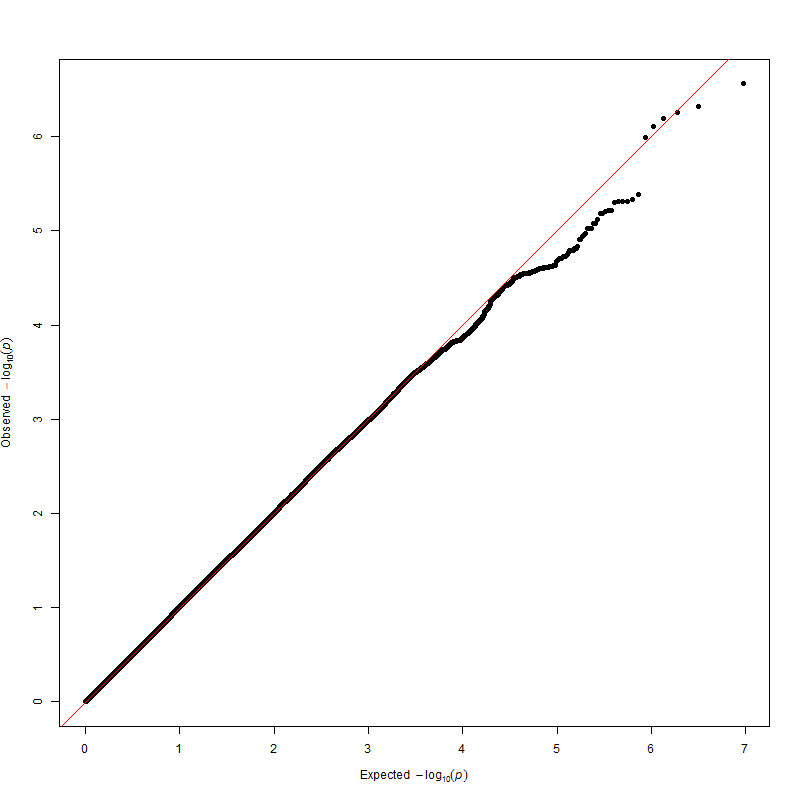


NTR


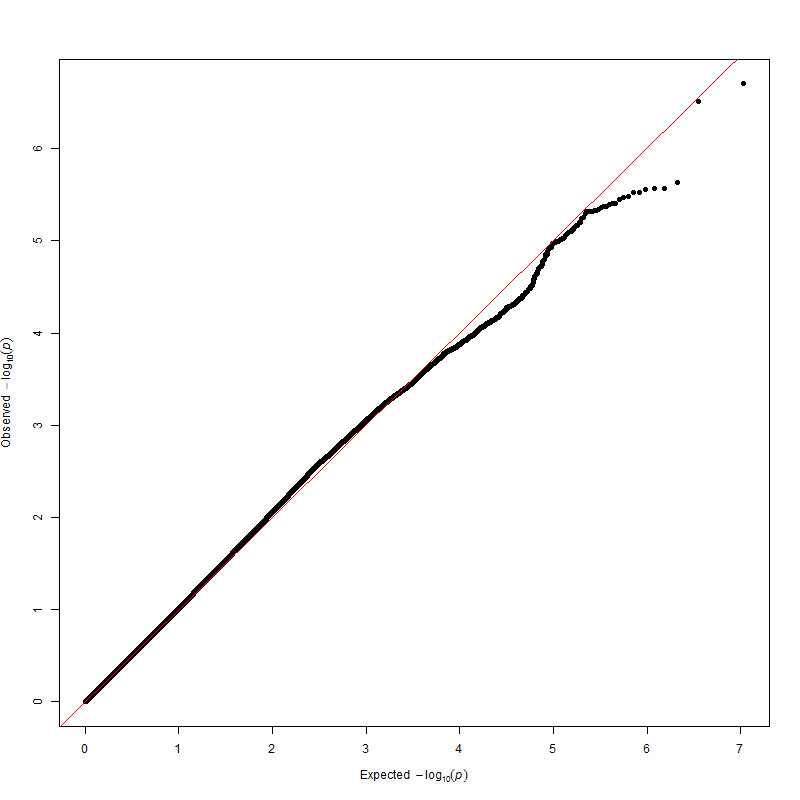


ORCADES


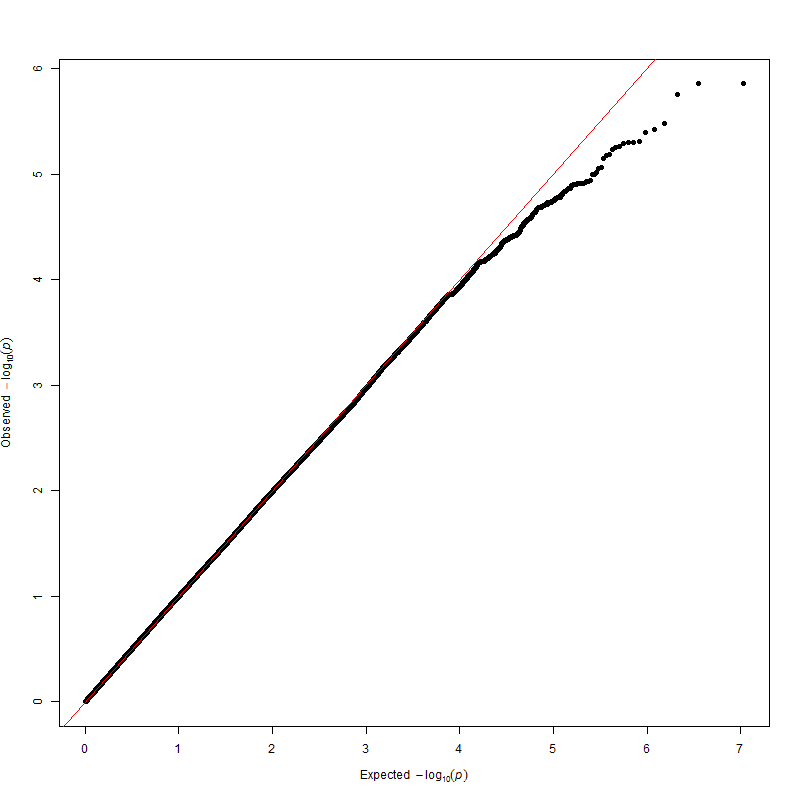


PAGES


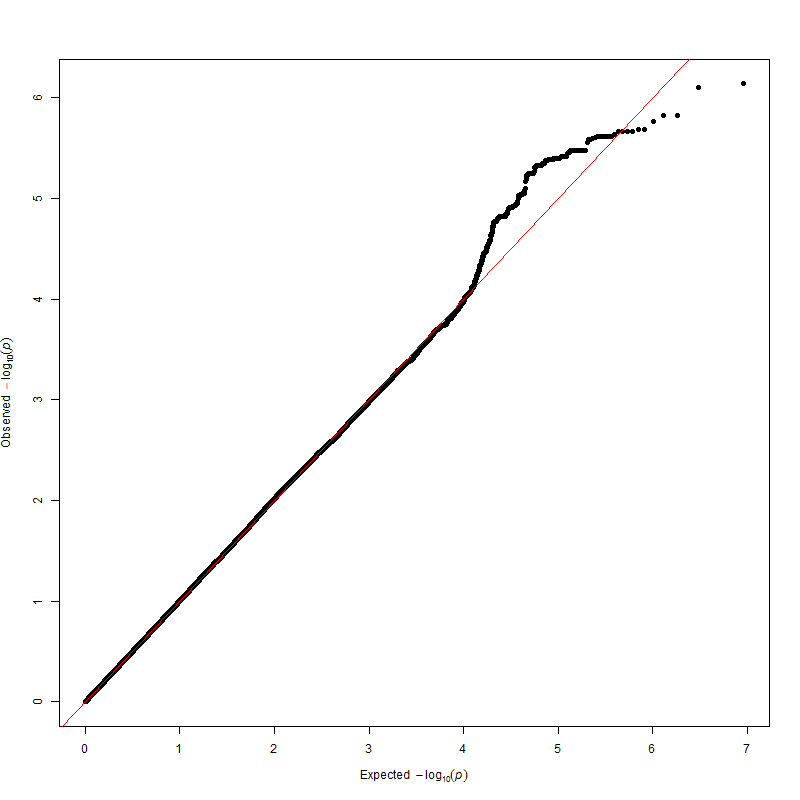


QIMR adolescents


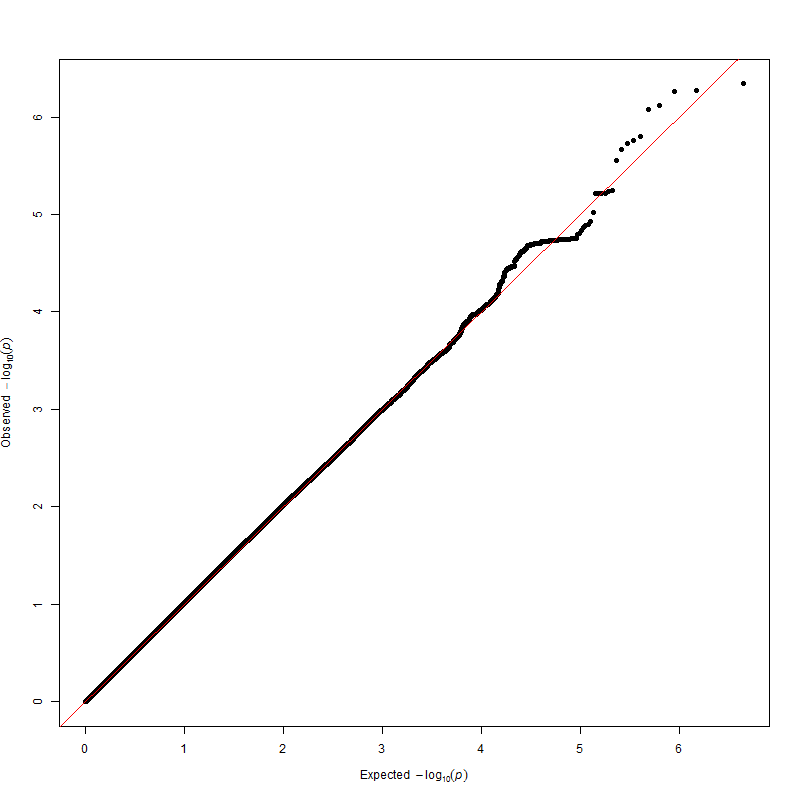


QIMR adults


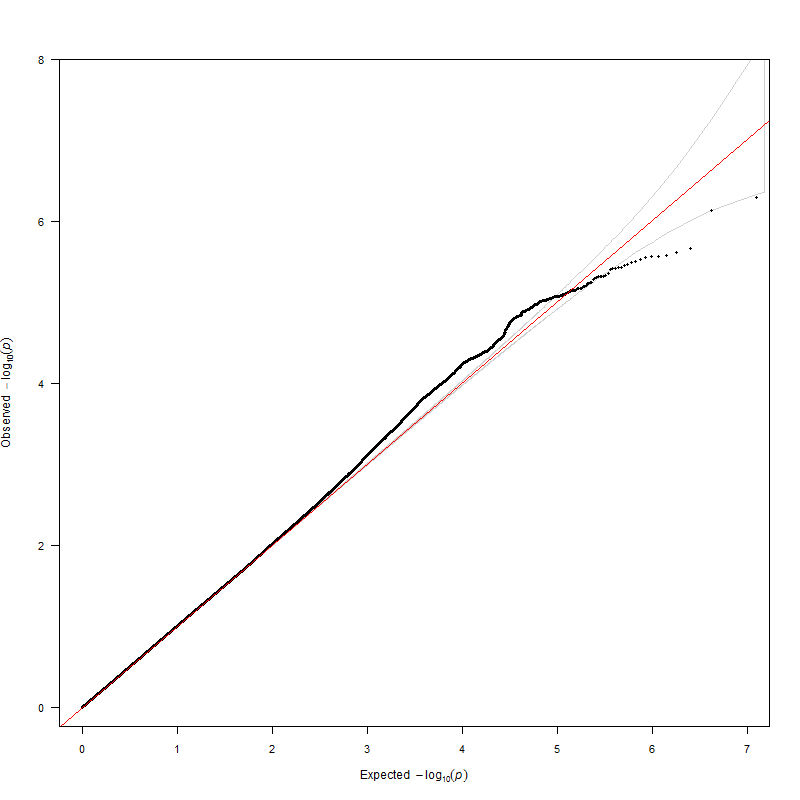


SAGE-COGA


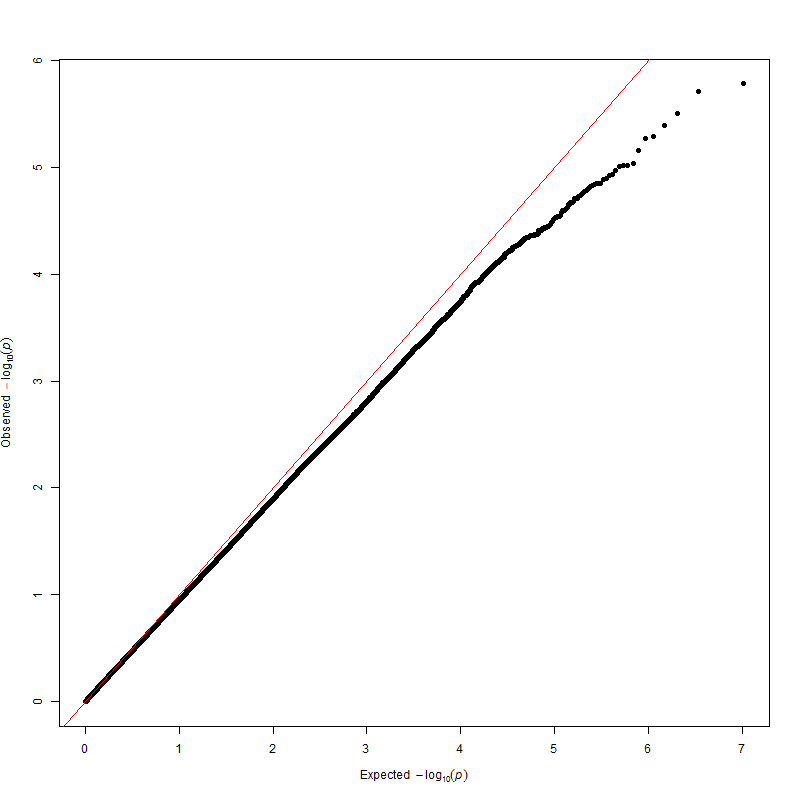


SAGE-COGEND


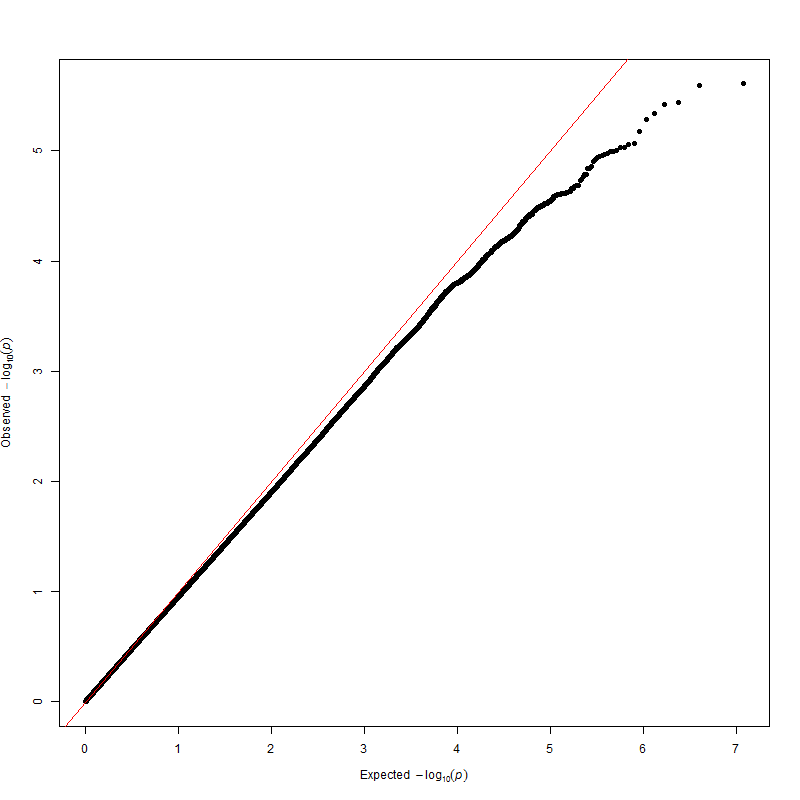


SardiNIA


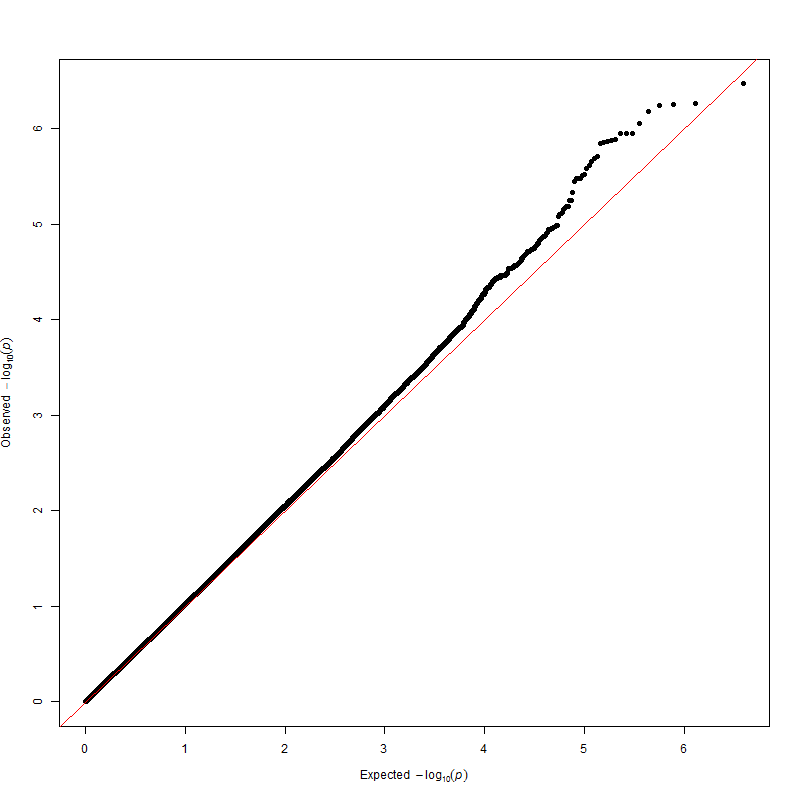


SHIP


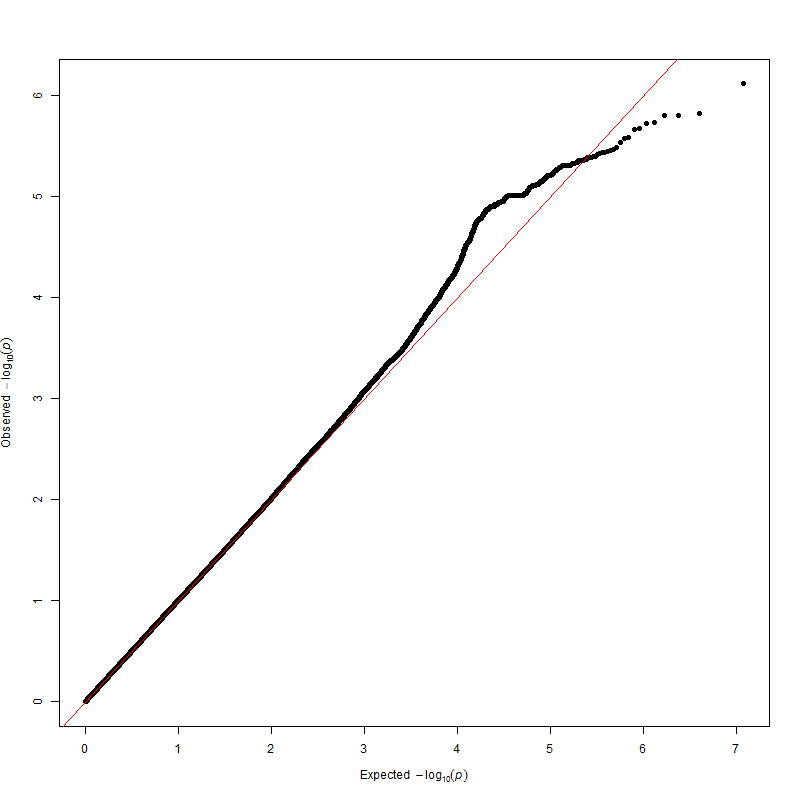


STR


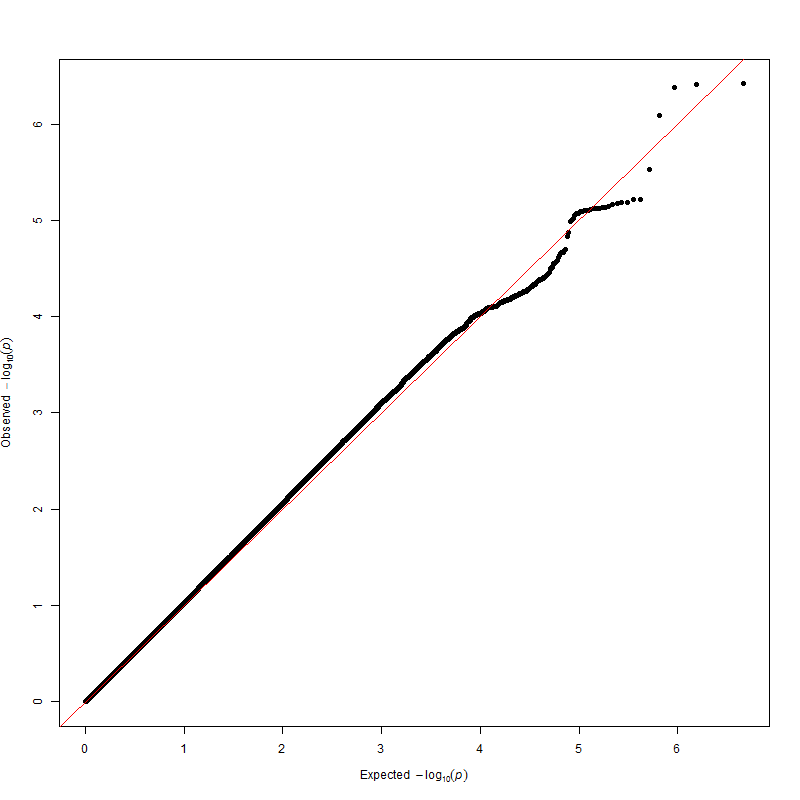


CROATIA-Vis


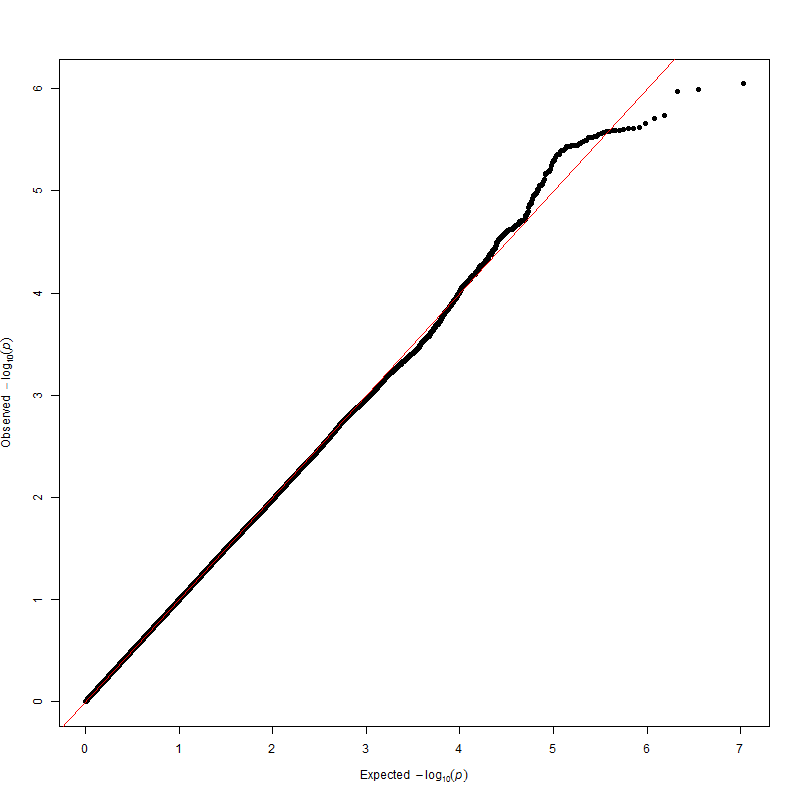


YFS


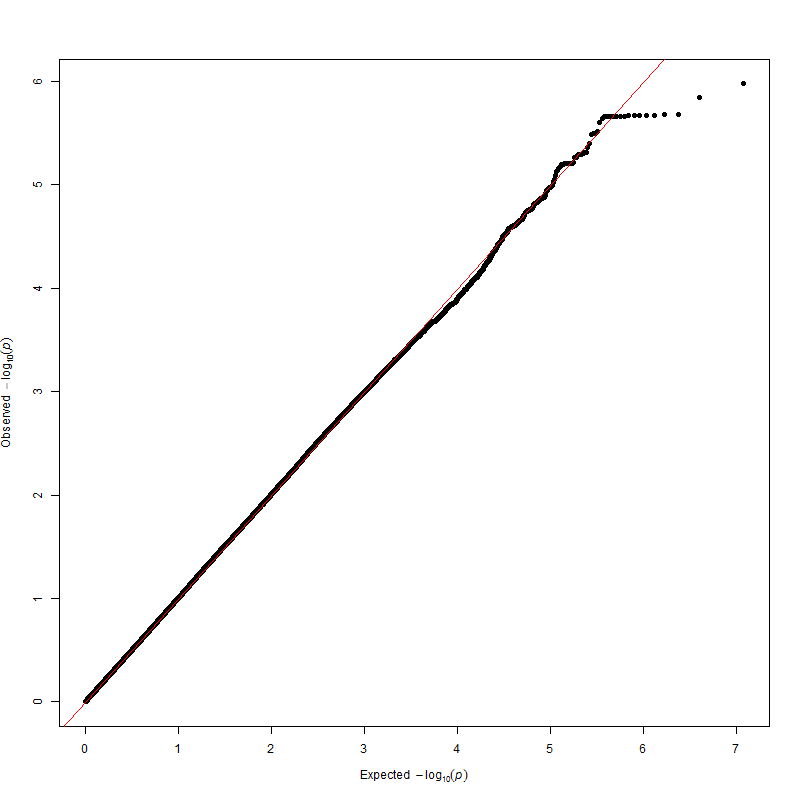


References

1. Boyd A, Golding J, Macleod J, Lawlor DA, Fraser A, Henderson J *et al.* Cohort Profile: The ‘Children of the 90s’—the index offspring of the Avon Longitudinal Study of Parents and Children. *International Journal of Epidemiology* 2013; **42**(1)**:** 111-127.

2. Terracciano A, McCrae RR, Brant LJ, Costa PT. Hierarchical linear modeling analyses of the NEO-PI-R scales in the Baltimore longitudinal study of aging. *Psychology and Aging* 2005; **20**(3)**:** 493-506.

3. Hart AM, Engelhardt BE, Wardle M, Sokoloff G, Stephens M, de Wit H *et al.* Genome-Wide Association Study of d-Amphetamine Response in Healthy Volunteers Identifies Putative Associations, Including Cadherin 13 (CDH13). *PLOS One* 2012; **7**(8)**:** e42646.

4. Colonna V, Nutile T, Astore M, Guardiola O, Antoniol G, Ciullo M *et al.* Campora: A young genetic isolate in South Italy. *Human Heredity* 2007; **64**(2)**:** 123-135.

5. Colonna V, Nutile T, Ferrucci RR, Fardella G, Aversano M, Barbujani G *et al.* Comparing population structure as inferred from genealogical versus genetic information. *European Journal of Human Genetics* 2009; **17**(12)**:** 1635-1641.

6. Metspalu A. The Estonian Genome Project. *Drug Development Research* 2004; **62**(2)**:** 97-101.

7. Pardo LM, MacKay I, Oostra B, van Duijn CM, Aulchenko YS. The effect of genetic drift in a young genetically isolated population. *Annals of Human Genetics* 2005; **69:** 288-295.

8. Barker DJP, Osmond C, Forsen TJ, Kajantie E, Eriksson JG. Trajectories of growth among children who have coronary events as adults. *New England Journal of Medicine* 2005; **353**(17)**:** 1802-1809.

9. Eriksson JG, Osmond C, Kajantie E, Forsen TJ, Barker DJP. Patterns of growth among children who later develop type 2 diabetes or its risk factors. *Diabetologia* 2006; **49**(12)**:** 2853-2858.

10. Raikkonen K, Pesonen AK, Heinonen K, Lahti J, Kajantie E, Forsen T *et al.* Infant growth and hostility in adult life. *Psychosomatic Medicine* 2008; **70**(3)**:** 306-313.

11. Polasek O, Marusic A, Rotim K, Hayward C, Vitart V, Huffman J *et al.* Genome-wide Association Study of Anthropometric Traits in Korcula Island, Croatia. *Croat Med J* 2009; **50**(1)**:** 7-16.

12. Deary IJ, Gow AJ, Pattie A, Starr JM. Cohort Profile: The Lothian Birth Cohorts of 1921 and 1936. *International Journal of Epidemiology* 2011; **Epub ahead of print**.

13. Deary IJ, Whiteman MC, Starr JM, Whalley LJ, Fox HC. The impact of childhood intelligence on later life: Following up the Scottish Mental Surveys of 1932 and 1947. *J Pers Soc Psychol* 2004; **86**(1)**:** 130-147.

14. Deary IJ, Gow AJ, Taylor MD, Corley J, Brett C, Wilson V *et al.* The Lothian Birth Cohort 1936: a study to examine influences on cognitive ageing from age 11 to age 70 and beyond. *BMC Geriatrics* 2007; **7:** 28.

15. Iacono WG, McGue M. Minnesota Twin Family Study. *Twin Research* 2002; **5**(5)**:** 482-487.

16. McGue M, Keyes M, Sharma A, Elkins I, Legrand L, Johnson W *et al.* The environments of adopted and non-adopted youth: Evidence on range restriction from the Sibling Interaction and Behavior Study (SIBS). *Behav Genet* 2007; **37**(3)**:** 449-462.

17. Kiemeney LA, Thorlacius S, Sulem P, Geller F, Aben KKH, Stacey SN *et al.* Sequence variant on 8q24 confers susceptibility to urinary bladder cancer. *Nat Genet* 2008; **40**(11)**:** 1307-1312.

18. Penninx BWJH, Beekman ATF, Smit JH, Zitman FG, Nolen WA, Spinhoven P *et al.* The Netherlands Study of Depression and Anxiety (NESDA): rationale, objectives and methods. *International Journal of Methods in Psychiatric Research* 2008; **17**(3)**:** 121-140.

19. Boomsma DI, Vink JM, van Beijsterveldt TC, de Geus EJ, Beem AL, Mulder EJ *et al.* Netherlands Twin Register: a focus on longitudinal research. *Twin Research* 2002; **5**(5)**:** 401-406.

20. Boomsma DI, de Geus EJC, Vink JM, Stubbe JH, Distel MA, Hottenga JJ *et al.* Netherlands Twin Register: From twins to twin families. *Twin Research and Human Genetics* 2006; **9**(6)**:** 849-857.

21. McQuillan R, Leutenegger AL, bdel-Rahman R, Franklin CS, Pericic M, Barac-Lauc L *et al.* Runs of homozygosity in European populations. *Am J Hum Genet* 2008; **83**(3)**:** 359-372.

22. Wright MJ, Martin NG. Brisbane adolescent twin study: Outline of study methods and research projects. *Australian Journal of Psychology* 2004; **56**(2)**:** 65-78.

23. Aitken JF, Green A, Eldridge A, Green L, Pfitzner J, Battistutta D *et al.* Comparability of Nevus Counts Between and Within Examiners, and Comparison with Computer Image-Analysis. *British Journal of Cancer* 1994; **69**(3)**:** 487-491.

24. Distel MA, Trull TJ, Derom CA, Thiery EW, Grimmer MA, Martin NG *et al.* Heritability of borderline personality disorder features is similar across three countries. *Psychol Med* 2008; **38**(9)**:** 1219-1229.

25. Medland SE, Nyholt DR, Painter JN, McEvoy BP, McRae AF, Zhu G *et al.* Common variants in the trichohyalin gene are associated with straight hair in Europeans. *Am J Hum Genet* 2009; **85**(5)**:** 750-755.

26. Pergadia ML, Agrawal A, Loukola A, Montgomery GW, Broms U, Saccone SF. Genetic linkage findings for DSM-IV nicotine withdrawal in two populations. *Am J Med Genet B* 2009; **150B:** 950-959.

27. Saccone SF, Pergadia ML, Loukola A, Broms U, Montgomery GW, Wang JC *et al.* Genetic linkage to chromosome 22q12 for a heavy-smoking quantitative trait in two independent samples. *Am J Hum Genet* 2007; **80**(5)**:** 856-866.

28. Heath AC, Jardine R, Eaves LJ, Martin NG. The genetic structure of personality I. Phenotypic factor structure of the EPQ in an Australian sample. *Personality and Individual Differences* 1988; **9**(1)**:** 59-67.

29. Slutske WS, Meier MH, Zhu G, Stratham DJ, Blaszczynski A, Martin NG. The Australian twin study of gambling (OZ-GAM): Rationale, sample description, predictors of participation, and a first look at sources of individual differences in gambling involvement. *Twin Research and Human Genetics* 2009; **12:** 63-78.

30. John U, Greiner B, Hensel E, Ludemann J, Piek M, Sauer S *et al.* Study of Health In Pomerania (SHIP): a health examination survey in an east German region: objectives and design. *Soz Praventivmed* 2001; **46**(3)**:** 186-194.

31. Volzke H, Alte D, Schmidt CO, Radke D, Lorbeer R, Friedrich N *et al.* Cohort profile: the study of health in Pomerania. *International Journal of Epidemiology* 2011; **40**(2)**:** 294-307.

32. Floderus-Myrhed B, Pedersen N, Rasmuson I. Assessment of heritability for personality, based on a short-form of the Eysenck Personality Inventory: a study of 12 898 twin pairs. *Behav Genet* 1980; **10:** 153-162.

33. Ivkovic V, Vitart V, Rudan I, Janicijevic B, Smolej-Narancic N, Skaric-Juric T *et al.* The Eysenck personality factors: Psychometric structure, reliability, heritability and phenotypic and genetic correlations with psychological distress in an isolated Croatian population. *Pers Indiv Differ* 2007; **42**(1)**:** 123-133.

34. Raitakari OT, Juonala M, Ronnemaa T, Keltikangas-Jarvinen L, Rasanen L, Pietikainen M *et al.* Cohort Profile: The Cardiovascular Risk in Young Finns Study. *International Journal of Epidemiology* 2008; **37**(6)**:** 1220-1226.

35. Smith BH, Campbell A, Linksted P, Fitzpatrick B, Jackson C, Kerr SM *et al.* Cohort Profile: Generation Scotland: Scottish Family Health Study (GS:SFHS). The study, its participants and their potential for genetic research on health and illness. *International Journal of Epidemiology* 2013; **42**(3)**:** 689-700.

36. Aulchenko YS, Ripke S, Isaacs A, van Duijn CM. GenABEL: an R library for genorne-wide association analysis. *Bioinformatics* 2007; **23**(10)**:** 1294-1296.

37. van den Berg SM, de Moor MH, McGue M, Pettersson E, Terracciano A, Verweij KJ *et al.* Harmonization of Neuroticism and Extraversion phenotypes across inventories and cohorts in the Genetics of Personality Consortium: an application of Item Response Theory. *Behav Genet* 2014; **44**(4)**:** 295-313.
